# The fruticose genera in the Ramalinaceae (Ascomycota, Lecanoromycetes): their diversity and evolutionary history

**DOI:** 10.3897/mycokeys.73.47287

**Published:** 2020-09-11

**Authors:** Richard Spjut, Antoine Simon, Martin Guissard, Nicolas Magain, Emmanuël Sérusiaux

**Affiliations:** 1 World Botanical Associates, PO Box 81145, Bakersfield, California 93380, USA World Botanical Associates Bakersfield, CA United States of America; 2 Evolution and Conservation Biology Unit, Sart Tilman B22, Quartier Vallée 1, chemin de la vallée 4, B-4000 Liège, Belgium Evolution and Conservation Biology Unit Liège Belgium

**Keywords:** Atacama, Baja California, Namib, *
Namibialina
*, *
Niebla
*, *
Ramalina
*, taxonomy, *
Vermilacinia
*, Vizcaíno deserts

## Abstract

We present phylogenetic analyses of the fruticose Ramalinaceae based on extensive collections from many parts of the world, with a special focus on the Vizcaíno deserts in north-western Mexico and the coastal desert in Namibia. We generate a four-locus DNA sequence dataset for accessions of *Ramalina* and two additional loci for *Niebla* and *Vermilacinia*. Four genera are strongly supported: the subcosmopolitan *Ramalina*, the new genus *Namibialina* endemic to SW Africa, and a duo formed by *Niebla* and *Vermilacinia*, endemic to the New World except the sorediate *V. zebrina* that disjunctly occurs in Namibia. The latter three genera are restricted to coastal desert and chaparral where vegetation depends on moisture from ocean fog. *Ramalina* is subcosmopolitan and much more diverse in its ecology.

We show that *Ramalina* and its sister genus *Namibialina* diverged from each other at c. 48 Myrs, whereas *Vermilacinia* and *Niebla* split at c. 30 Myrs. The phylogeny of the fruticose genera remains unresolved to their ancestral crustose genera.

Species delimitation within *Namibialina* and *Ramalina* is rather straightforward. The phylogeny and taxonomy of *Vermilacinia* are fully resolved, except for the two youngest clades of corticolous taxa, and support current taxonomy, including four new taxa described here. Secondary metabolite variation in *Niebla* generally coincides with major clades which are comprised of species complexes with still unresolved phylogenetic relationships. A micro-endemism pattern of allopatric species is strongly suspected for both genera, except for the corticolous taxa within *Vermilacinia*. Both *Niebla* and saxicolous *Vermilacinia* have chemotypes unique to species clades that are largely endemic to the Vizcaíno deserts.

The following new taxa are described: *Namibialina***gen. nov.** with *N. melanothrix* (**comb. nov.**) as type species, a single new species of *Ramalina* (*R. krogiae*) and four new species of *Vermilacinia* (*V. breviloba*, *V. lacunosa*, *V. pustulata* and *V. reticulata*). The new combination *V. granulans* is introduced. Two epithets are re-introduced for European *Ramalina* species: *R. crispans* (= *R. peruviana* auct. eur.) and *R. rosacea* (= *R. bourgeana* auct. p.p). A lectotype is designated for *Vermilacinia procera*. A key to saxicolous species of *Vermilacinia* is presented.

## Introduction

The genus *Ramalina* Ach. is one of the best known lichen genera, easily recognized and widely studied by scientists in various fields of research, including biomonitoring of environmental changes (Agnan et al. 2016; López Berdonces et al. 2016), evaluation of impacts of industrial activities to the environment (Domínguez-Morueco et al. 2015), biotoxicity (Anar et al. 2015), use in cancer therapy (Lee et al. 2016; Suh et al. 2017) and other human diseases (Kim et al. 2018; Furmanek et al. 2019), biotechnologies (Biosca et al. 2016), decontamination (Candan et al. 2017), and even in ethnological studies of rural human populations (Devkota et al. 2017).

Furthermore, the genus is at the cutting edge of research about the very nature of lichenization as several species [mostly *R. farinacea* (L.) Ach.] have been shown to host and use several strains or even species of their green algal partners within the same thallus (Casano et al. 2015; Catalá et al. 2016; Moya et al. 2017). It is also a model for physiological studies of the lichen as a distinct entity (Sanders and Tokamov 2015; Sanders and de los Ríos 2019) and of the physiological dimension of symbiosis (Guéra et al. 2016).

As the genus *Ramalina* is subcosmopolitan and easily detected, it is almost always included in any floristic account, usually with ecological and biogeographical data, of any area in the world (Gasparyan and Sipman 2016; Diederich et al. 2017; Paukov et al. 2017); several include conservation assessment (McMullin 2015; Sparrius et al. 2017). Species new to science continue to be described, including those from unexpected ecological niches, such as riverside rocks submitted to a continuous water spray (Gumboski et al. 2018).

Yet the evolutionary history of the genus is poorly known, with a positioning within the Lecanorales, suborder Sphaerophorinae in the Lecanoromycetes (Miadlikowska et al. 2014). The genus *Ramalina* is included in the well-supported Ramalinaceae s.l. together with well-known genera such as *Bacidia* De Not., *Bacidina* Vězda, *Biatora* Fr.: Fr., *Bilimbia* De Not., *Lecania* A. Massal., *Phyllopsora* Müll. Arg. and *Toninia* A. Massal. The Ramalinaceae s. str. (Sérusiaux et al. 2012) excludes *Bacidia*, *Bacidina*, *Byssolecania* Vain., the *Lecania chlorotiza* group and *Toninia* that are assigned to the Bacidiaceae. The remaining genera form a highly variable assemblage, a basal and poorly supported clade including *Megalaria grossa* (Pers. ex Nyl.) Haffellner and *Lopezaria versicolor* (Flot.) Kalb and Haffelner as sister to a much stronger supported clade, including *Cliostomum griffithii* (Sm.) Coppins and *Vermilacinia cephalota* (Tuck.) Spjut and Hale as sister to four accessions of *Ramalina* (Miadlikowska et al. 2014).

Bowler (1981) studied cortical anatomy features of the subfruticose or typically fruticose Ramalinaceae, relating his findings to chemical, chorological and ecological data. He empirically recognized seven genera: *Cenozosia* A. Massal. [type: *C. inanis* (Mont.) A. Massal.], *Dievernia* Choisy [type: *D. ramulicola* M. Choisy), *Fistulariella* Bowler and Rundel [type: *F. inflata* (Hook. F. and Taylor) Bowler and Rundel], *Niebla* Rundel and Bowler [type: *N. homalea* (Ach.) Rundel and Bowler], *Ramalina* Ach. [type: *R. fraxinea* (L.) Ach.], *Ramalinopsis* (Zahlbr.) Follm. and Huneck [type: *R. manii* (Zahlbr.) Follmann and Huneck] and *Trichoramalina* Rundel and Bowler [type: *T. crinita* (Tuck.) Rundel and Bowler]. Spjut (1995a) segregated the new genus *Vermilacinia* Spjut and Hale [type species: *V. combeoides* (Nyl.) Spjut and Hale] from *Niebla*.

Sérusiaux et al. (2010) provided the first assessment of the North American *Niebla* (sensu Rundel and Bowler 1978; Bowler and Marsh 2004) within an evolutionary context using molecular sequence data; however, sampling was limited to relatively few accessions of *Vermilacinia*, while the *Ramalina bourgeana* group in the Mediterranean and Macaronesian regions in Europe (Krog and Østhagen 1980; Bowler and Marsh 2004; Aptroot and Schumm 2008) were shown to be nested within *Ramalina*. Additionally, accessions representative of two other genera, *Dievernia* and *Fistulariella* (Bowler and Rundel 1977; Bowler 1981) were also resolved within *Ramalina* s.l.

More recently, using a 5-locus dataset, Kistenich et al. (2018) produced a comprehensive phylogeny of the family, including a larger set of tropical taxa (formerly assigned to *Crocynia* (Ach.) Massal., *Eschatogonia* Trev., *Krogia* Timdal, *Phyllopsora*, *Physcidia* Tuck. and others). They included accessions of the monotypic *Cenozosia* Massal. (endemic to the Atacama Desert in South America) and *Ramalinopsis* (Zahlbr.) Follm. and Huneck (endemic to the Hawaii archipelago), as well as both species assigned to *Trichoramalina* (*T. crinita*, endemic to the Pacific coast of California, USA and Baja California, Mexico and *T. melanothrix*, endemic to the coastal desert of Namibia and South Africa). They resolved *T. crinita* within *Ramalina* s.l. and *T. melanothrix* as sister to a strongly supported clade of “*Niebla homalea*” (= *Vermilacinia laevigata*) and “*Niebla combeoides*” (= *V. combeoides*), based on Bowler and Marsh (2004), which included *Vermilacinia*, exhibiting “extreme plasticity in morphological appearance”. Further, they showed that *Cenozosia* is the sister group to all fruticose genera and, finally, that *Ramalinopsis* and *Trichoramalina* can be reduced into synonymy with *Ramalina*.

## Objectives of this study

In this study, our first objective was to revisit the delimitation of the “fruticose” genera within the Ramalinaceae, their phylogenetical relationships and biogeography, with a special focus on *Niebla* and *Vermilacinia* sensu Spjut (1996), endemic to the coastal deserts alongs the Pacific coasts of the New World and the enigmatic species *Ramalina angulosa* and *R. melanothrix*, endemic to coastal deserts in SW Africa.

The second objective was to evaluate the diversity within *Niebla* and *Vermilacinia* with molecular data and statistical inferences in a phylogenetic context. We wish to compare the taxonomical treatment of both genera proposed by Spjut (1996) with our DNA sequence data. The rationale of this work was to give taxonomic weight to chemical characters and to delimit species within each “chemical group” by morphological patterns. Chemical characters have phytogeographical significance; for example, ß-depsidones-producing thalli in *Niebla* are almost all endemic to the Northern Vizcaíno Desert (NVD) and often terricolous, in contrast to depside-producing terricolous thalli occurring on San Nicolas Island. As a result, 42 species were recognized in *Niebla* and 18 in *Vermilacinia* (Spjut 1996). This taxonomical treatment was denied by Bowler and Marsh (2004) in the “Lichen Flora of the Greater Sonoran Desert Region”, without detailed evaluation and under the presumption that all morphological characters are highly plastic and unworthy of consideration in a taxonomical treatment.

Our third and last objective was to evaluate the phylogenetic variation within the genus *Ramalina* s. str., with an expanded sampling compared to the current available data (Sérusiaux et al. 2010) and with several species that are quite variable and that may unveil cryptic taxa. Such species include *R. breviuscula* Nyl., *R. fastigiata* (Pers.) Ach., *R. requienii* (De Not.) Jatta, *R. subfarinacea* (Nyl. ex Cromb.) Nyl. and *R. tingitana* Salzm. Indeed, two recent studies unveiled an impressive and unexpected phylogenetic variation: the well-known epiphytic species of the western coasts of North America *R. menziesii* strongly structured in well-delimited lineages (Sork and Werth 2014) and the puzzling *R. pollinaria* shown to be a three species complex (Gasparyan et al. 2017).

As indicated above, three geographical areas play a special role in the evolutionary history and the present range of the fruticose genera of the Ramalinaceae: (I) the coasts of California/USA and Baja California/Mexico; (II) the Atacama and Sechura deserts along the western coasts of South America and (III) the coasts of Namibia and the South-West of South Africa. These areas are briefly presented in Suppl. material 1, focusing on their biodiversity, especially for lichenized fungi and their recent climatic history. The botanical significance of each of these is briefly discussed. Suppl. material 1 further includes updates (with Spjut 1996 as the seminal reference) on the ecogeographical data and evolutionary interpretation for the genera *Niebla* and *Vermilacinia* in Baja California.

## Material and methods

### Sampling and identification of collections

**Figure 1.**
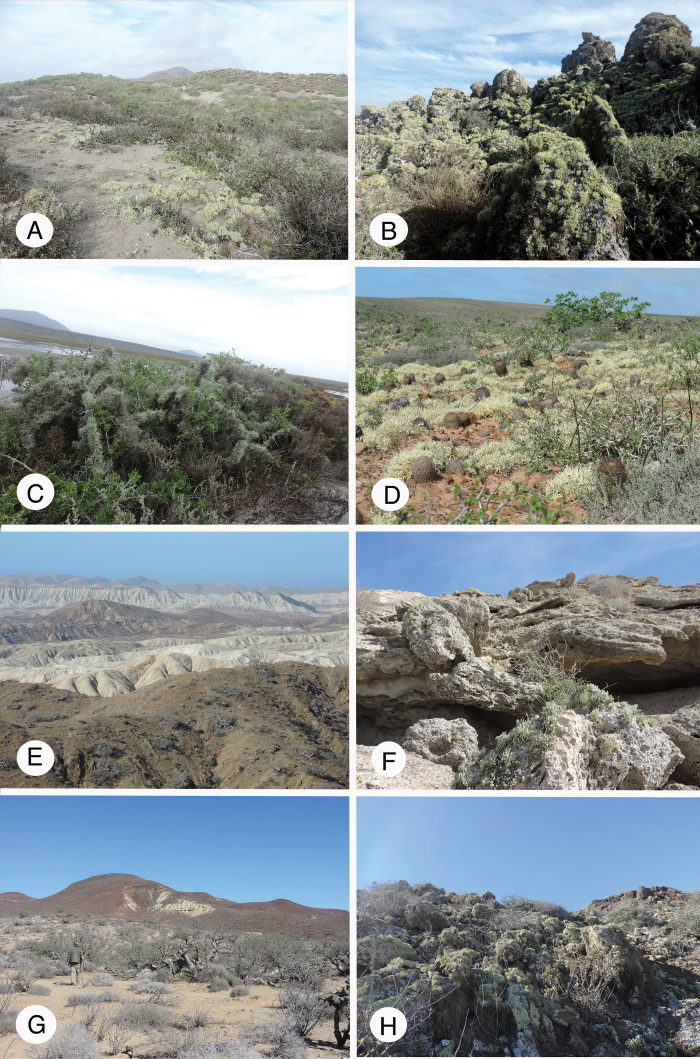
Landscapes of Baja California and Baja California Sur. **A** Arenicolous species of *Niebla* in sand dunes at San Quintín BC**B** saxicolous species of *Niebla* over dark volcanic rocks at San Quintín BC**C** twigs of *Lycium* covered with fruticose *Roccella* and *Vermilacinia* at San Quintín BC**D** Free-living species of *Niebla* on “red” rocky slopes at Punta Baja BC**E** landscapes of the peninsula de Vizcaíno **F** gypsicolous rocks near the sea at Bahia Ascunsion BCS with *Niebla lobulata***G** chaparral near Bahia Tortugas BCS **H** outcrops subjected to intermittent fog near Bahia Tortugas BCS. Photographs by E. Sérusiaux and R. Spjut.

**Figure 2.**
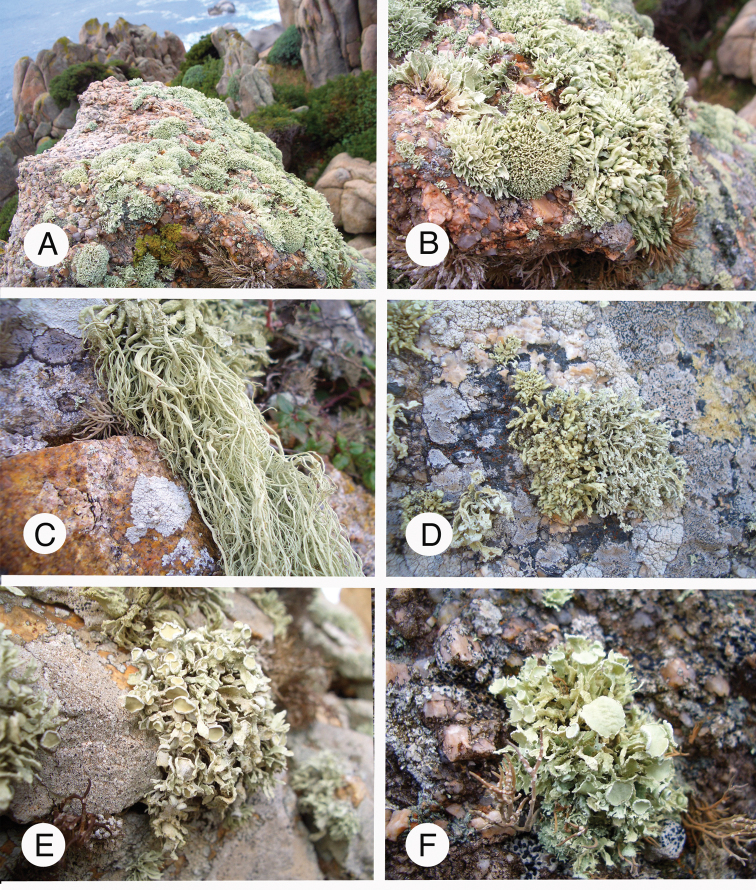
Species of *Ramalina* on rocky seashores in Italy/Sardinia **A** general view of species and habitat **B** from left to right: *R. tingitana*, *R. breviuscula* and *R. cribrosa***C***R. implexa***D** from left to right: *R. clementeana* and *R. requienii***E***R. tingitana***F***R. inaequalis*. Photographs by M. Guissard and E. Sérusiaux.

**Figure 3.**
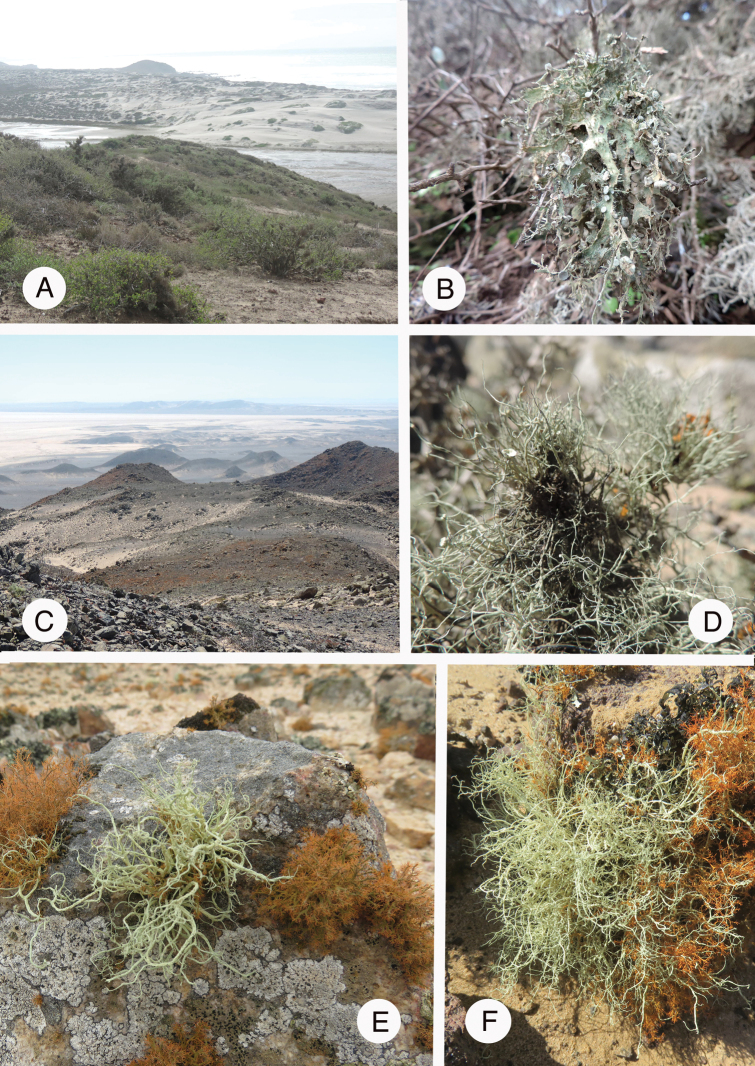
**A** Landscape in Baja California, San Quintín where *Ramalina crinita* thrives **B***R. crinita*; **C** Landscape of the Skeleton Coast Park in Namibia where *Namibialina* “*angulosa*” and *N. melanothrix* thrive **D***N. melanothrix***E–F***N.* “*angulosa* 1” (**E**) and *N.* “*angulosa* 2” (**F**). Photographs by E. Sérusiaux.

Almost all collections used for this study were gathered by the authors during several field trips, especially to Mexico/Baja California and Baja California Sur (Figs 1, 3), USA/California and Namibia/coastal desert (Fig. 3). Material was further collected in France, including Corsica, Italy/Sardinia (Fig. 2), the Canary Islands, Madeira, including Porto Santo, the Azores, Armenia, Norway, Rwanda, Switzerland and Taiwan. We further added samples collected by other workers, *inter alia*, P. van den Boom in the Cabo Verde archipelago and T. Raus and H. Sipman in Greece.

During the field trip to Baja California and Baja California Sur, fruticose Ramalinaceae were sampled from 31 localities, treated as 11 broader collection areas (Suppl. material 6: Table S6), along the Pacific coast of North America, starting at Cabo Colonet, south of Ensenada (Mexico/Baja California: 31°04'24"N, 116°12'28"W) to Guerrero Negro, then southwest to Bahía Asunción (Mexico/Baja California Sur: 27°08'18"N, 114°17'45"W) and northwest to Punta Eugenia (Figs 1, 5). Each locality was carefully sampled and, for all material collected, the ITS region has been sequenced. A set of recent collections from the coasts of California (USA) assembled in 2017 was also included. However, no recent collections of *Niebla* from the islands off the coasts of California and Baja California were available; three endemic species occur on these islands, two on San Nicolas Island (*N. dactylifera* and *N. ramosissima*) and a third (*N. sorediata*) on Isla Guadalupe and San Clemente Island (type locality); *N. dilatata* and *N. isidiosa*, previously reported endemic to Isla Guadalupe, have since been discovered on the Baja peninsula. For *Vermilacinia*, the same protocol was adopted, except for the *V. leopardina* group (here referred to as “black-banded species group” that includes *V. howei*, *V. nylanderi*, *V. tigrina* and *V. zebrina*) whose variation needs further study. An accession of the sorediate *V. zebrina* from the Atlantic coast of Namibia has been included (Wirth 2010a, b).

Unfortunately, we were unable to include material of *Vermilacinia* from South America. Several species of *Vermilacinia* occur in the Atacama desert (Peru and Chile): the terricolous *V. ceruchis*, which is related to North American saxicolous species and corticolous *V. flaccescens*, “*Niebla*” *granulans* (Sipman 2011), *V. leonis*, *V. leopardina*, “*Niebla*” *nashii* (Sipman 2011) and *V. tigrina* (also terricolous). *Vermilacinia cephalota* was reported by Sipman (2011), but we consider this to be possibly *V. leonis*. However, Spjut (BRY loan) identified V. aff. acicularis, V. aff. robusta, *V. procera* and *V. varicosa*, reportedly collected in the Atacama Desert, May 2017; these specimens were not included in the molecular sampling.

During the field trip to Namibia, we sampled *Ramalina* s.l. from the coastal desert, between Swakopmund (22°20.389'S, 014°26.446'E) and Cape Cross (21°39.319'S, 013°59.550'E) where the so-called “lichen fields” are well-developed and partly protected. The sampling was extensive, but restricted to a short portion (ca. 120 km long) of the coast enjoying fog and thus providing appropriate ecological conditions for lichen communities, that spread from southern Angola down to the Cape of Good Hope in South Africa.

**Figure 4.**
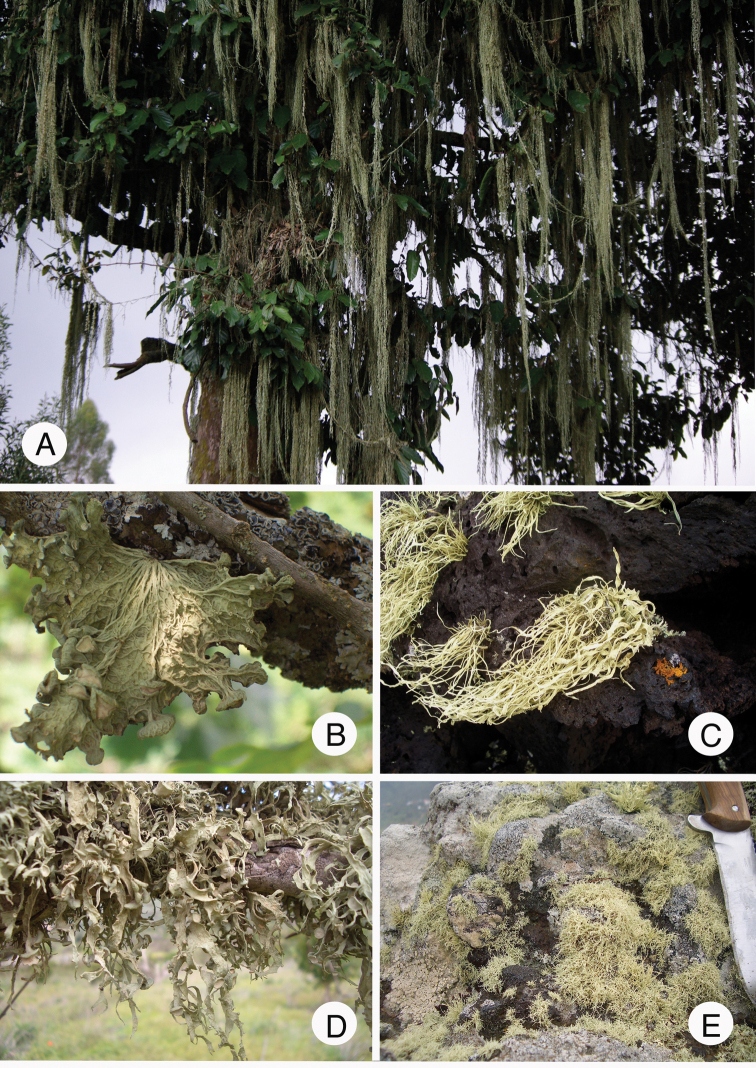
Several species of *Ramalina***A***R. hoehneliana*, hanging down the branches of a large *Strombosia scheffleri* in Gishwati forest (Rwanda) **B***R. sinensis* (Armenia) **C***R. azorica* (Azores, Pico) **D***R. huei* (Canary Is., Tenerife) **E***R. nodosa* (Canary Is., Tenerife). Photographs by E. Sérusiaux.

We included most accessions used by Sérusiaux et al. (2010), including material from USA/California and Mexico/Baja California. We added accessions of species expected to be resolved at the base of the *Ramalina* s. str. Tree [e.g. *R. fraxinea*, *R. hoehneliana*, *R. polymorpha* and *R. sinensis* (Fig. 4)]. For the latter species, assumed to be subcosmopolitan that consistently resolved at the base of all other accessions of *Ramalina*, we added accessions from Asia, Europe and North America. Both species of “*Trichoramalina*” (*T. crinita* and *T. melanothrix*; Fig. 3) were included along with the sequences provided by Kistenich et al. (2018). We assessed several variable species of *Ramalina* from different geographical areas: *R. breviuscula* (including from type locality), *R. fastigiata*, *R. requienii*, *R. subfarinacea* and *R. tingitana*. We further focused on saxicolous species thriving along sea-shores of islands in the Western Mediterranean region [Italy: Sardinia, France: Corsica (Fig. 2) and Spain: Cabo del Gata].

**Figure 5.**
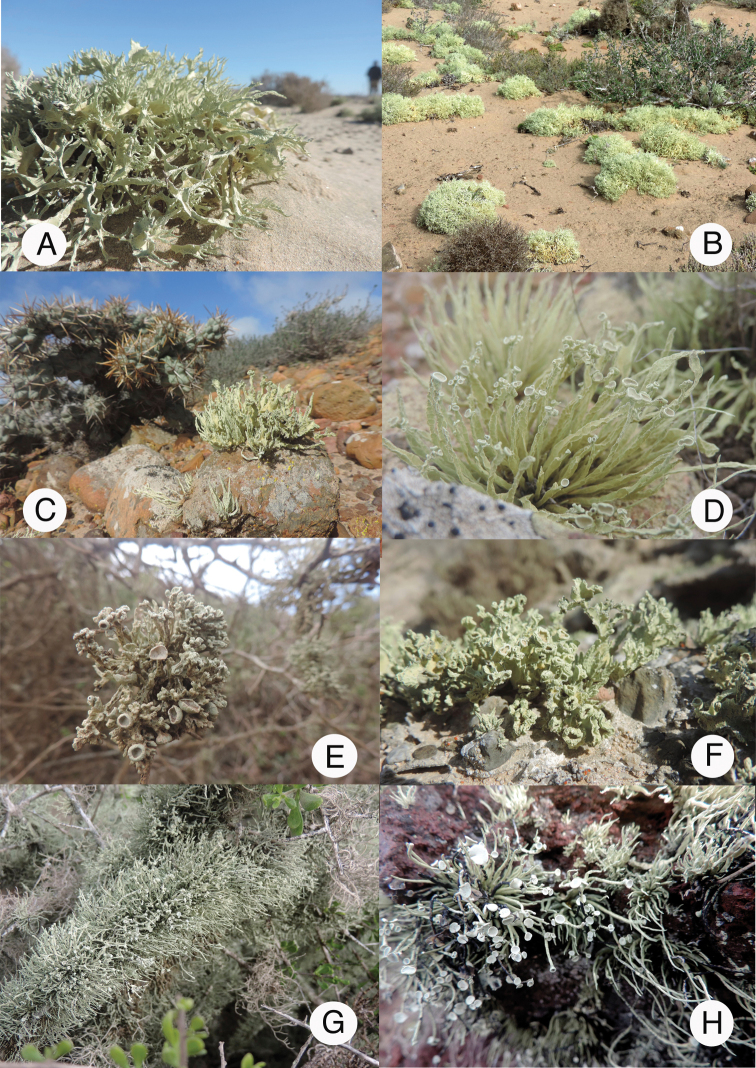
Several species of *Niebla* and *Vermilacinia*. Identifications based on Spjut (1996)**A***Niebla limicola* on sand dunes at Guerrero Negro BCS **B***N. marinii* at Santo Domingo BC**C***N. podetiaforma* at Punta Baja BC**D***N. siphonoloba* at San Antonio BC**E***Vermilacinia cerebra* at San Antonio BC**F***Niebla lobulata* at Bahía de Tortugas BCS **G***Vermilacinia leopardina* on branches at San Quintín BC**H***V. procera* on rocks at San Quintín BC. Photographs by E. Sérusiaux and R. Spjut.

Finally, we added two accessions retrieved from GenBank, *R. complanata* and *Vermilacinia cephalota* (both collected in the USA) for which the four targeted loci were available; they were included in the phylogenetic synthetic analysis of the Lecanoromycetes by Miadlikowska et al. (2014) and are here used as references to check consistency.

Identification of collections was performed with the following support: Spjut (1996) for the collections of *Niebla* and *Vermilacinia* from USA and Mexico; the accession of *Vermilacinia* from Africa/Namibia was also assessed following Wirth (2010a, b); Krog and Østhagen (1980) and Aptroot and Schumm (2008) for the material gathered in Macaronesia (archipelagos of the Canary Islands, Madeira, Porto Santo and the Azores), Krog and James (1977) and the French translation of the *Ramalina* chapter of Clauzade and Roux (1985) provided by G. Duclaux for material from continental Europe and Armenia; and Swinscow and Krog (1988) for collections from Rwanda (Africa).

In order to assess validly published epithets appropriate for several species, type collections and related material were examined at the Natural History Museum in London (BM), the “Museum National d’Histoire Naturelle” in Paris (PC), the “Institut Botànic de Barcelona” (BC) and the Herbarium of the University of Barcelona (BCN).

Specimens at the University of Liège (Liège, Belgium) were studied using standard microscopic techniques. Morphological descriptions are based on observations using a Leica S4E dissecting microscope (Leica Microsystems GmbH, Wetzlar, Germany) and a Nikon Eclipse 80i compound microscope (Nikon Corporation, Tokyo, Japan). Thin-layer chromatography (TLC) was carried out following Orange et al. (2010) using solvents B (*Niebla*), C and G. Specimens were deposited at LG; specimens gathered in Mexico/Baja California and Baja California Sur in 2016 were deposited in BCMEX, LG and the private herbarium of World Botanical Associates (WBA); those sampled in USA/California were deposited in LG and WBA.

### DNA extraction and loci amplification

For the overall analysis of the generic delimitation of the target genera (*Niebla*, *Ramalina* s.l. and *Vermilacinia*), we included two representatives of the Psoraceae (*Protoblastenia calva* and *Psora rubiformis*) and one representative of the Tephromelataceae (*Tephromela atra*) as outgroups, following Miadlikowska et al. (2014) and Kistenich et al. (2018). We further included representatives of the Bacidiaceae (*Bacidia schweinitzii*, “*B. sorediata*”, *Bacidina arnoldiana*, *Biatora vernalis* and *Thalloidema sedifolia*), as well as *Lopezaria versicolor* and *Megalaria grossa*. Finally, we included selected representatives of *Phyllopsora* s.l. in order to include all lineages featuring this thallus type in the dataset (*Eschatagonia prolifera*, *Parallospora leucophyllina*, *Phyllopsora breviuscula*, *P. gossypina*, *P. chlorophaea* and “*P. borbonica*”) following the results of Kistenich et al. (2018). These provide calibration points for the time calibration of our phylogenetic tree.

Two datasets were analyzed independently for this study:

– Matrix 1: a four-locus dataset (ITS-LSU-RPB1-RPB2) comprising a selection of accessions for *Niebla* (37 out of 101) and *Vermilacinia* (19 out of 46) and all representatives (9) of *Ramalina angulosa* and *R. melanothrix* (these two species are assigned to the new genus *Namibialina*) and *Ramalina*, except for one accession of *R. rosacea* and all accessions of *R. sarahae* (102 out of 112). Accessions included in Matrix 1 are marked with X in the first column of Suppl. material 3: Table S3.

– Matrix 2: a six-locus dataset (ITS-LSU-RPB1-RPB2-GDP-EF-1α) comprising all representatives of *Niebla* and *Vermilacinia* (147 specimens), with *R. farinacea* and *R. tingitana* as outgroup.

For further information regarding the sequences generated and used in this study, see Suppl. material 3: Table S3.

### Molecular phylogenetic analyses

Multiple sequence alignments were performed with MAFFT using the auto option (Katoh et al. 2002; Katoh et al. 2009) as implemented in Geneious 10.0.7 (Biomatters Ltd., Auckland, New Zealand) and were carefully checked by eye and manually adjusted. For each dataset, ambiguous alignment sites in the ITS, LSU, and EF-1α markers were excluded using the GBLOCKS server 0.91b http://molevol.cmima.csic.es/castresana/Gblocks_server.html, with settings allowed to produce the least stringent selection (Castresana 2000). Intronic regions within RPB1 and RPB2 were manually removed when present. The GAPDH locus was analyzed with all sites included. Prior to concatenation and for each dataset, single-locus phylogenetic trees were produced for each marker via RAxML-HPC2 8.2.3 (Stamatakis 2006; Stamatakis et al. 2008) on the CIPRES portal (Miller et al. 2010; http://www.phylo.org) using the rapid hill-climbing algorithm and bootstrapping with 1000 pseudo-replicates under a GTR+G model of evolution. Inspection of gene tree incongruence was performed using compat.py (Kauff and Lutzoni 2002). Significant conflict was detected only within *Ramalina* pointing to a variable position of two lineages (*R. clementeana* and the *R. fastigiata* gr.). The matrices were nevertheless concatenated and the phylogenetic analysis applied to these data, because the phylogenetic placement of these two groups was not the focal point of this study.

PartitionFinder 2 (Lanfear 2016) was used to determine the best partitioning schemes and nucleotide substitution models for the subsequent analyses. For Matrix 1, eight initial subsets were considered (ITS; LSU; RPB1 1^st^, 2^nd^, 3^rd^ codon positions; RPB2 1^st^, 2^nd^, 3^rd^ codon positions). Seven additional subsets were considered for Matrix 2 (GAPDH 1^st^, 2^nd^, 3^rd^ codon positions; EF-1α 1^st^, 2^nd^, 3^rd^ codon positions and introns). PartitionFinder 2 was run with the default configuration settings (branchlengths = linked, model_selection = BIC, search = greedy); for the ML analyses, the GTR+G model was the only one allowed.

### Evolutionary tree for all fruticose Ramalinaceae and age calibration

We ran an analysis on Matrix 1 running BEAST2 v.2.6.1 (Bouckaert et al. 2014) as implemented on the CIPRES portal. We unlinked substitution models but linked clocks and trees across loci. Models of sequence evolution for each subset were determined by the Bayesian Information Criterion (BIC; Schwarz 1978) in jModelTest 2.1.6 (Darriba et al. 2012) on the CIPRES Science Gateway (ITS: TrNef+I+G; LSU: TrN+I+G; RPB1: K80+I+G; RPB2: TrNef+I+G). A lognormal relaxed clock was implemented. Two fossil priors with lognormal prior distributions were set to calibrate the tree: one on the monophyly of *Phyllopsora* with an age of [(5% quantile –) median (–95% quantile)] (16.4-)17.8(-19.3) Myrs following Rikkinen and Poinar (2008) and the other on the monophyly of Ramalinaceae with an age of (116-)126(-137) Myrs following Rivas Plata (2011). The tree was generated with a Yule model. Trees were sampled every 1000^th^ generation. Convergence was assessed using Tracer v.1.7.1 (Rambaut et al. 2018). The run was initially set up to 500 million generations, but since all ESS values were superior to 200 and stationarity appeared to be reached, the analysis was stopped after 399 600 000 generations. The first 129 600 000 generations were discarded as burn-in, then one tree every 20 000^th^ generation was selected, resulting in a sample of 13 500 trees which was used to generate a final consensus tree with TreeAnnotator.

### Evolutionary tree for the genera *Niebla* and *Vermilacinia*

A maximum likelihood analysis was performed on Matrix 2 with RAxML 8.2.3 on the CIPRES portal using the rapid hill-climbing algorithm and bootstrapping with 1000 pseudoreplicates under a GTR+G model of evolution for each partitioned subset. We provide the bootstrap results obtained with the method recently developed by Lemoine et al. (2018). Indeed, phylogenies, based on hundreds of taxa, tend to have low supports with Felsentein’s method (Felsenstein 1985), based on resampling and replications; the new version of the phylogenetic boostrap introduced by Lemoine et al. (2018) is designed to address this matter without inducing falsely supported branches. The results of the ML analyses were visualized with the R package ggtree (Yu et al. 2012).

### Species delimitation analyses within *Niebla* and *Vermilacinia*

Species delimitation was inferred from molecular data following four methods: ABGD (Puillandre et al. 2012), PTP (Zhang et al. 2013), BPP (Rannala and Yang 2003) and STACEY (Jones 2015). Each genus (*Niebla* and *Vermilacinia*) was treated individually (these lineages were isolated into separate input files) and without outgroups, except for the STACEY analysis. For BPP, the genus *Niebla* was further divided into two subgroups [Clades “depsides” and “β-depsidones”] in order to make the analysis less computationally-intensive.

A first species delimitation was performed using the ITS dataset only (extracted from Matrix 2), following the ABGD method (Puillandre et al. 2012). This method uses short DNA sequences to assign organisms into species. The automatic procedure is based on the barcode gap, which can be observed whenever the divergence amongst organisms belonging to the same species is smaller than divergence amongst organisms from different species. Based on the input data, the method uses a range of priors to infer from the data a model-based one-sided confidence limit for intraspecific divergence, then detects the barcode gap as the first significant gap beyond this limit and uses it to partition the data. Inference of the limit and gap detection are then recursively applied to previously obtained groups to get finer partitions until there is no further partitioning. The ABGD analyses were carried out on the two genera separately (*Niebla* and *Vermilacinia*) on the ABGD website (https://bioinfo.mnhn.fr/abi/public/abgd/abgdweb.html). Default parameters were chosen using Kimura (K80) genetic distances for each analysis and testing for different relative gap width values (X = 0.1, 0.5, 1.0, 1.5). Ultimately, the following partitions were selected: 17 groups of *Niebla* (P = 2.78e-03; X = 0.1) and 16 groups of *Vermilacinia* (P= 2.78e-03; X = 0.5).

The second method uses the so-called Poisson tree processes model (PTP; Zhang et al. 2013). It is a coalescent-based species delimitation method, which utilizes the number of substitutions to infer putative species boundaries on the trees built via RAxML (Matrix 2). While this method is usually intended for delimiting species in single-locus phylogenies, this programme may also be used on concatenated gene trees when there are no strong conflicts between gene trees (Luo et al. 2018; Bustamante et al. 2019). We chose to use it here on our concatenated tree obtained from Matrix 2 in order to objectively assign each specimen into input candidate species for the subsequent BPP (Bayesian Posterior Probability) analysis. Additionally, PTP was run on the corresponding single-locus ITS trees and the obtained results are included in Fig. 7. All analyses were run on the PTP web server (https://species.h-its.org/ptp/) using the default parameters and 500,000 MCMC (Markov Chain Monte Carlo) generations.

The third method uses a Bayesian MCMC implementation of the MultiSpecies Coaslescent model, which allows both species delimitation and species tree inference (Rannala and Yang 2003; Yang and Rannala 2010, 2014, 2017). This method is known as BPP and the analyses were run for a total of 500,000 MCMC generations, thinning set to 100. The delimitation followed the result of the ML solution. The multi-locus coalescent-based BPP v. 3.3 analysis was performed using the above mentioned ML trees as guide trees (i.e. analysis A10). The divergence time parameter tau (τ) was estimated using the root height of the guide trees and was set to G(2, 60) (corresponding to distribution mean of 0.03). The population size parameter theta (θ) gamma prior was tested for different values: G(4, 1000), G(8, 1000) and G(12, 1000). The matrix was analyzed using the reversible-jump Markov Chain Monte Carlo (rjMCMC) algorithms implemented in the programme BPP, sampling every generation for a total of 5,000,000 generations, with a burn-in period of 500,000. We used the posterior mean (P = 0.5) as the probability threshold above which input groups are considered heterospecific. Each BPP analysis was carried out twice to confirm the consistency between runs.

The fourth method is the STACEY package, implemented in BEAST2 (Bouckaert et al. 2014; Jones 2015): it does not require a priori assignment of each individual accession to a putative species and, further, is a Bayesian method. Indeed, although it is sensitive to the choice of priors, it can explore the entire space of species trees including evolutionary processes causing discordance, such as ILS (Incomplete Lineage Sorting). The STACEY approach has been successfully applied to species delimitation in several lichen groups (Boluda et al. 2019; Gerlach et al. 2019; Mark et al. 2019; Lutsak et al. 2020) and seems to avoid the overestimation of the number of potential species usually encountered with BPP (Edwards et al. 2016).

We ran a *BEAST analysis as implemented in BEAST2 v. 2.6.1 with the STACEY module enabled. Substitution models were determined using jModeltest as above. Substitution models were TN93+G for EF and TN93+I+G for GDP. Exponential relaxed clocks were used. The analysis was run for 500 million generations, sampling every 10 000^th^ generation. We discarded the first 9 960 000 generations as burn-in and kept a tree every 25 000^th^ generation, resulting in a sample of 19 600 trees which was used to generate a consensus species tree with TreeAnnotator. The graphical display of the STACEY matrix was generated using R version 3.2.1 (R core team 2015). Visual display of trees with categorical columns was generated using R package ggtree (Yu et al. 2017).

### Evolutionary tree for *Ramalina* s.l.

This tree encompasses all accessions of *Ramalina* (except for one accession of *R. rosacea* and all accessions of *R. sarahae*) and those assigned to the new genus *Namibialina* and is based on accessions included in Matrix 1. Therefore, the tree is a subset of the analysis performed on Matrix 1; all calibrations and parameters are identical.

## Results

Altogether, we generated DNA sequence data for a total of 283 specimens of the Ramalinaceae sensu lato (Suppl. material 3: Table S3). The concatenated Matrices 1 and 2 consisted of 3,312 positions (ITS: 410 bp; LSU: 1063 bp; RPB1: 1011 bp; RPB2: 828 bp) and 4,466 positions (ITS: 457 bp; LSU: 1,262 bp; RPB1: 1,011 bp; RPB2: 828 bp; GAPDH: 531 bp; EF-1α: 377 bp), respectively.

### Evolutionary tree for all fruticose Ramalinaceae and age calibration (Fig. 6)

A single strongly supported branch sustains all accessions of fruticose taxa in the Ramalinaceae. Strong support is detected for the delimitation of two lineages for the fruticose genera: (1) *Ramalina* as sister to a strongly delimited group endemic to the coastal desert in SW Africa assigned to the new genus *Namibialina*, the relationship between the two genera being strongly supported; (2) two genera (*Niebla* and *Vermilacinia*), endemic to coastal deserts along the Pacific coast in the New World, strongly supported together and, further, sister to three species of *Cliostomum*, including the type species (*C. corrugatum*), but with weak support. Both accessions of the crustose *Cliostomum griffithii* form a lineage sister to the strongly supported clade, including all other lineages studied: *Cliostomum* s. str., *Namibialina*, *Niebla*, *Ramalina* and *Vermilacinia*. Therefore, the genus *Cliostomum*, as delimited by Ekman (1997), is polyphyletic; and emergence of fruticose taxa within the Ramalinaceae is not a unique event.

**Figure 6.**
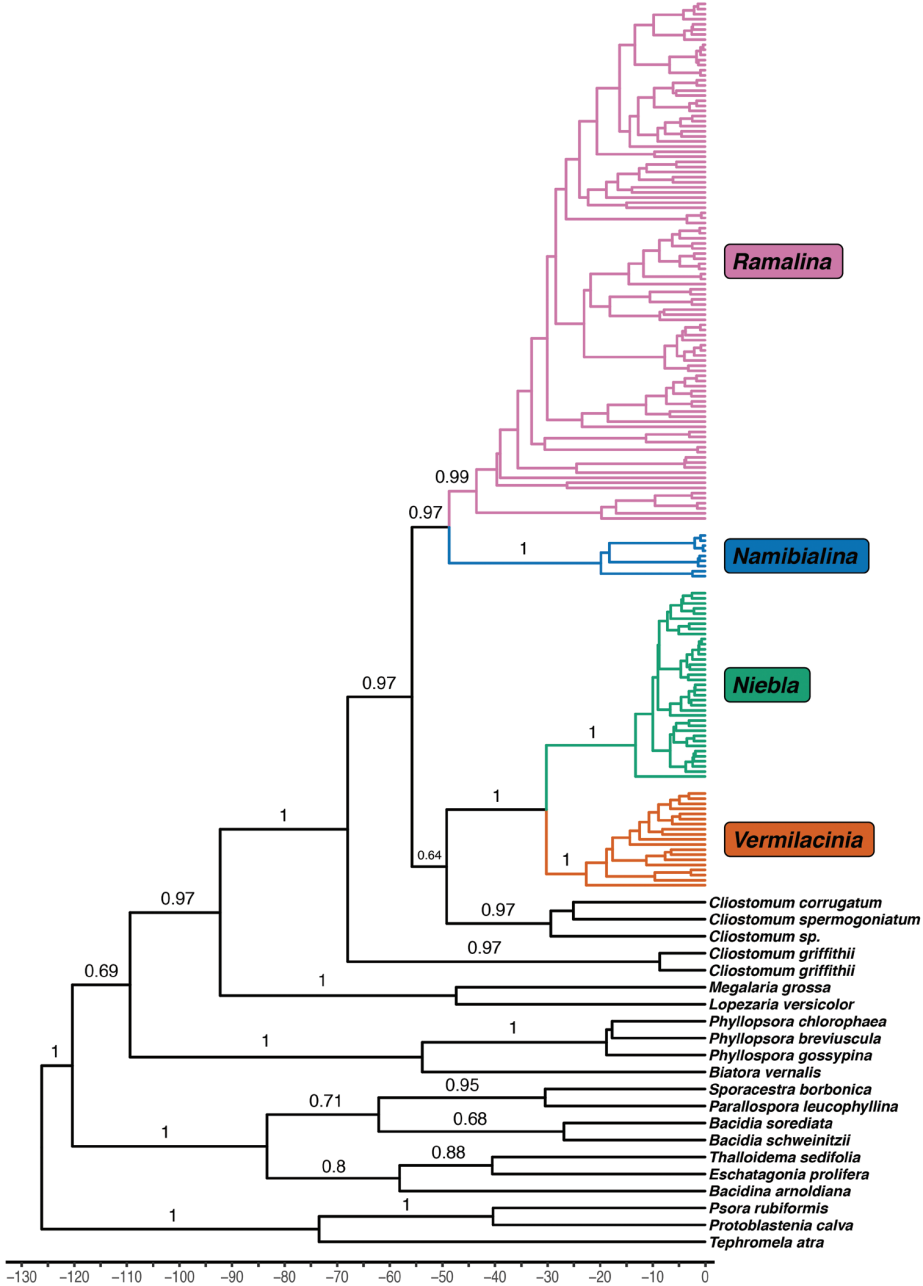
Time-calibrated phylogenetic tree generated by the BEAST2 analysis on four loci (Matrix 1), for the four genera of fruticose Ramalinaceae studied and their crustose sister genus *Cliostomum*: *Namibialina*, *Niebla*, *Ramalina* and *Vermilacinia*. Values above branches represent the posterior probabilities of support.

The time calibration, based on a fossil of *Phyllopsora*, yielded results (Suppl. material 4: Table S4) consistent with several studies for the time calibration of the Lecanoromycetes, closest to the *Lecanora* crown (sensu Prieto and Wedin 2013: table 3; Beimforde et al. 2014; Samarakoon et al. 2016; Huang et al. 2019).

The mean divergence time of the clade including all accessions of the fruticose Ramalinaceae plus their crustose sister taxon (*Cliostomum* s. str., including the genus type *C. corrugatum*) is 55.53 Myrs [95% highest posterior density (HPD) interval: 40.23–72.29] at the boundary between the Paleocene and the Eocene.

The emergence of the duo *Ramalina* + *Namibialina* is dated at 48.45 Myrs (HDP: 35.13–63.66) at the middle of Eocene. The duo *Niebla* + *Vermilacinia* diverged from one another at 30.05 Myrs (HDP: 17.27–43.11) during the Oligocene period; this date is almost identical to the diversification within *Cliostomum* s. str. Interestingly, *Vermilacinia* diversified starting at the beginning of the Miocene, 22.47 Myrs (HDP: 3.44–32.06), whereas *Niebla* began later, mid-Miocene, at 13.14 Myrs (HDP: 7.05–21.05). *Namibialina* diversified at 19.71 Myrs (HDP: 8.47–32.75) almost at the same time as the diversification within the basal species of *Ramalina*, *R. sinensis*.

### Evolutionary tree for the genera *Niebla* and *Vermilacinia* and species delimitation

**Figure 7.**
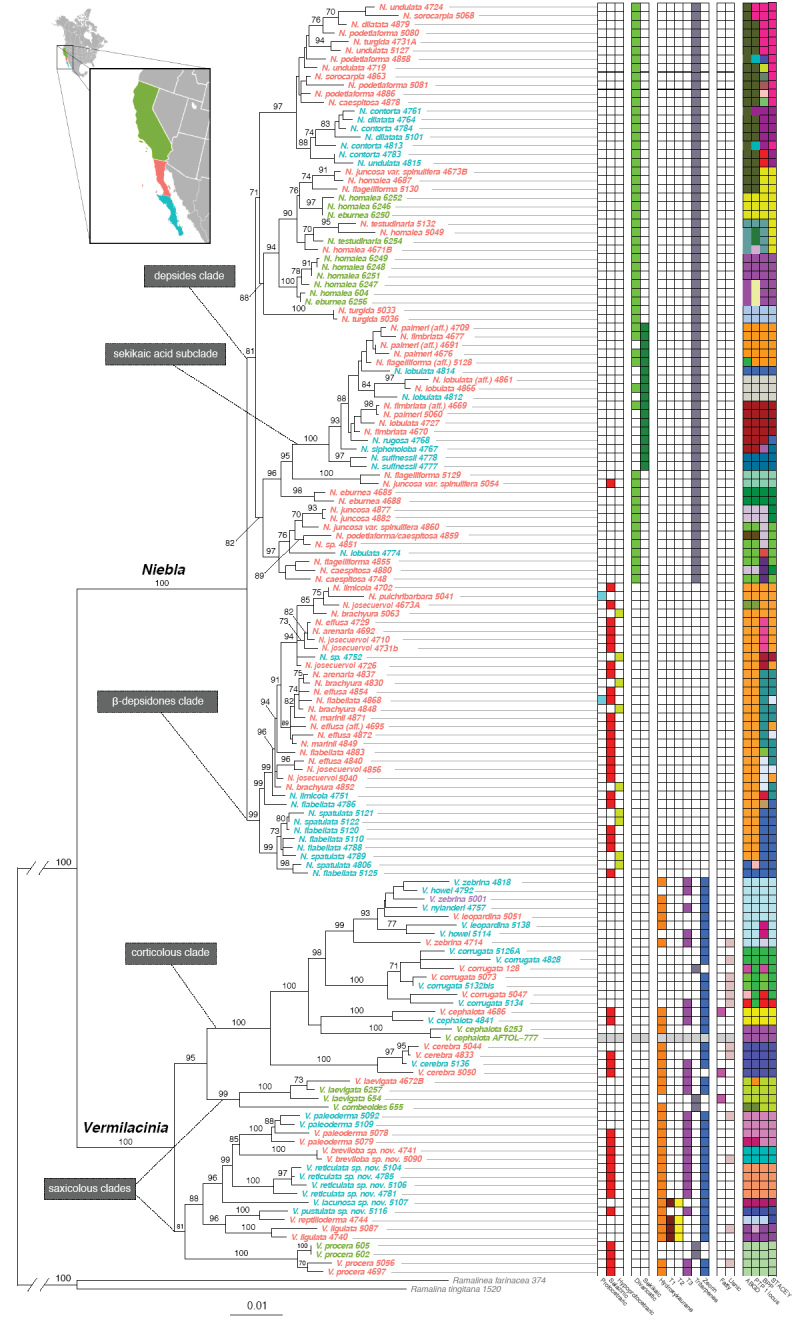
Evolutionary tree for the genera *Niebla* and *Vermilacinia*, produced with the 6-locus matrix (Matrix 2) and using RAxML. Support value for branches follow Lemoine et al. (2018). Epithets in colour following insert: green = collected in USA/California; pink = collected in Mexico/Baja California; blue = collected in Mexico/Baja California Sur. Table on right side provides further information for all accessions: column 1-3: ß-depsidones (protocetraric acid, salazinic acid, hypoprotocetraric acid); column 4-5: depsides (divaricatic acid, sekikaic acid); column 6-11: [-]-16α-hydroxykaurane, triterpenes T1, T2, T3, unidentified triterpenes, zeorin; column 12-13: fatty acid, usnic acid; greyish colour through columns 1-13 for one accession (*V. cephalota*) means that no data are available; column 14–17: results of species delimitation methods: 14 = ABGD; 15 = PTP on 1 locus; 16 = BPP; 17 = STACEY.

Results of the species delimitation methods are summarized in Fig. 7 and in Suppl. material 3: Table S3.

The two genera *Niebla* and *Vermilacinia*, as circumscribed by Spjut (1995a, 1996), are strongly supported. *Vermilacinia* is divided into two strongly supported groups (Fig. 7): (1) all saxicolous or terricolous species, except for two, *V. laevigata* and *V. combeoides* and (2) the sister clade includes these two saxicolous species that form a supported group, sister to a widely distributed corticolous group in the New World and Namibia.

**Figure 8.**
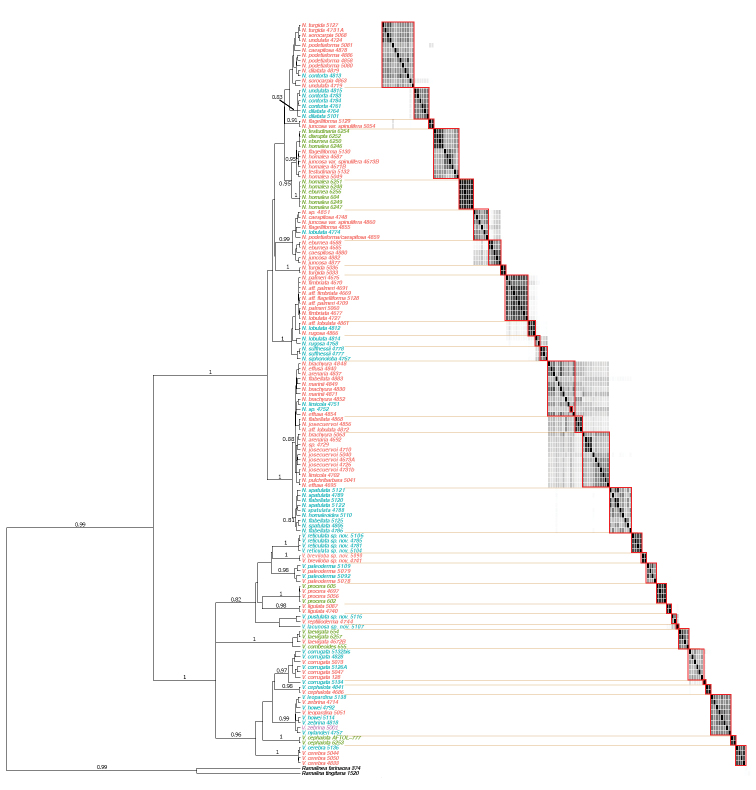
Evolutionary tree for the genera *Niebla* and *Vermilacinia*, produced with the 6-locus matrix and using *BEAST (Matrix 2). Values above branches represent posterior probabilities of support. Epithets in colour following insert: green = collected in USA/California; pink = collected in Mexico/Baja California; blue = collected in Mexico/Baja California Sur. Similarity matrix from the STACEY analysis on the right. Each rectangle represents posterior probability (white = 0, black = 1) of pairs of specimens to belong to the same species. Shades of grey represent intermediate values. Rectangles delimited by red lines represent the species delimitation with a 0.3 cut-off.

The genus *Niebla* is divided into two clades (Fig. 7). One is strongly supported by the absence of triterpenes and defined by the presence of medullary ß-depsidones; the other of depside species with triterpenes is less clearly defined and can be interpreted as one polytomy of at least five strongly supported clades (BS > 90%), four producing divaricatic acid and a fifth with basal species producing the same acid with a terminal, strongly supported node of sekikaic acid species with or without divaricatic acid.

The branching within the “ß-depsidones” clade does not discriminate amongst the three secondary medullary metabolites (protocetraric, salazinic and hypoprotocetraric acids).

Following Spjut’s identification key (1996), nine ß-depsidone species were identified by secondary metabolites followed by morphology. None is supported even though 9 putative species are recognized by the BPP analysis. Other delimitation methods recognized fewer putative species: STACEY five species, PTP four species and the ABGD three species.

The “depsides” clades are also very diverse with little match between the identification following Spjut (1996) and the phylogeny-based statistical species delimitation methods. Within these “depsides” clades, the BPP method recognized 24 putative species, the ABGD method 22, PTP 16 and STACEY 13.

In order to evaluate the evolutionary scenario that might be hidden under such discrepancies, we built three data tables for accessions of *Niebla* (Suppl. material 5: Table S5, Suppl. material 6: Table S6, and Suppl. material 7: Table S7): the first one compares the number of accessions in the dataset with the number of species delimited by BPP and STACEY; the other two with the number of species as recognized by BPP and STACEY within each locality. These data demonstrate: (a) no inclusive pattern can be detected with the first dataset (Suppl. material 5: Table S5), as the data spread from a complete match between the classical identification and the BPP and STACEY methods (as shown with the unique example *N. turgida*: two accessions are identified as a single species by the three methods) to the complete reverse (as shown by *N. lobulata*, where four accessions, recognized as that single species by the classical method, are recognized as four different taxa by the BPP and STACEY methods); Spjut (1996) reported differences in spore length and metabolites for *N. lobulata* collections in the Southern Vizcaíno Desert (SVD) and in the Northern Vizcaíno Desert (NVD); (b) the number of species as recognized by BPP (33 in our dataset) and STACEY (18) confirm a highly diverse genus as shown by Spjut (1996), but the number of species per locality ranges (Suppl. material 6: Table S6) from one to eight. The number of localities where a species is found is very low: with BPP data, mostly between one and three, a single case with four and another with five, over a total of 12 localities sampled; with STACEY data, also mostly between one and three, with two cases with four and a single case with six.

The phylogenetic tree for *Vermilacinia* (Fig. 7) is fully resolved at most nodes if one adopts a 90% BS value as reference and at all nodes if one adopts a > 80% BS value. The tree is divided into two main clades: one includes only saxicolous species with an almost perfect match between the ITS-barcode ABGD method and the other three more sophisaticated methods for species delimitation. This clade includes four species recognized as new in this work. These are monophyletic and supported lineages that cannot be assigned to any of the species included in Spjut (1996).

The second clade has two branches: one with the saxicolous *V. laevigata*, not distinguished by the BPP and STACEY methods from the related *V. combeoides* and the other one with all accessions of epiphytic species, including the populations found in SW Africa. A single species, as circumscribed by Spjut (1996), is recovered by our statiscal analyses: *V. cerebra*, representing the sister group to all other species. The other supported clades and statistical analyses demonstrate a complex situation: *V. cephalota*, one of two sorediate species in the Northern Hemisphere, is resolved into two different species, whereas the *V. corrugata* and the *V. howei-leopardina-nylanderi-zebrina* clades are unresolved: the BPP analysis recognized four species within *V. corrugata* and only three in the former assemblage; the STACEY analysis recognized two species in the *V. corrugata* lineage and only one in the *V. howei-leopardina-nylanderi-zebrina* one. Fifteen collections of *Vermilacinia* from Namibia all have the same ITS, including a single unique substitution in ITS2. The *Vermilacinia corrugata* clade is comprised of cryptic species, although Spjut (1996) described a morphological variation that might be segregated in further study; the type is in southern Baja California Sur where we did not collect.

### Evolutionary tree for the genera *Namibialina* and *Ramalina* (Fig. 9)

**Figure 9.**
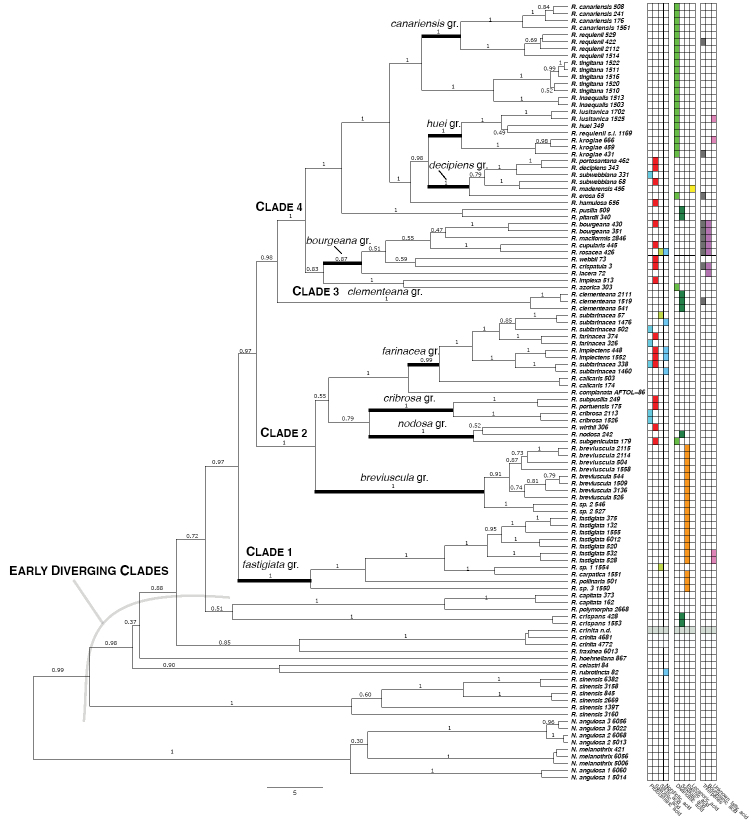
Evolutionary tree for the genera *Namibialina* and *Ramalina* (subset of Matrix 1). The tree is a close-up of Figure 6. Values above branches represent the posterior probabilities of support. Table on right side provides further information for all accessions: column 1-4: ß-depsidones (protocetraric acid, salazinic acid, stictic acid, norstictic acid); column 5-8: depsides (divaricatic acid, sekikaic acid, evernic acid, lecanoric acid); column 9: triterpenes; column 10: bourgeanic acid; column 11: unknown fatty acid; greyish colour through columns 1-11 for two accessions (*R. complanata* and *R. crinita*) means that no data are available.

A paraphyletic assemblage of ten species forms the base of the phylogenetic tree of *Ramalina* (Fig. 9: “early diverging clades”). This includes all seven species that lack medullary compounds (*R. capitata*, *R. celastri*, *R. crinita*, *R. fraxinea*, *R. hoehneliana*, *R. polymorpha* and *R. sinensis*) and two that produce a ß-depsidone or a depside, *R. rubrotincta* and *R. crispans*, respectively. Neither group forms a supported clade. A more extensive sampling for the basal *R. sinensis*, which is geographically structured, revealed an impressive diversification, dated at 19 Myrs.

Following these early diverging clades, is a strongly supported clade resolved in four assemblages, all strongly supported. However, significant incongruence amongst the four loci has been detected at this level, affecting the topological position of two lineages, the *R. clementeana* one with a single species and the *R. fastigiata* lineage with *R. carpatica*, *R.* sp. 1 and the eponym species. This matter remains to be resolved.

Apart from the paraphyletic early diverging assemblage, the tree here produced is divided into four main clades. The first (Fig. 9) includes the *fastigiata* gr.; the second includes the *brevisucula* gr., the *nodosa* gr., the *cribrosa* gr. and the *farinacea* gr.; the 3^rd^ includes only *R. clementeana*, while the fourth is much more diverse, including, *inter alia*, the *bourgeana*-, the *decipiens*, the *huei*- and the *canariensis* groups.

## Discussion

### Phylogeny of fruticose genera in the Ramalinaceae

The phylogenetic tree, here produced for the fruticose taxa within the Ramalinaceae, is strongly supported and rejects their monophyly. Indeed, both lineages that support fruticose genera are nested within accessions referred to the crustose genus *Cliostomum* s.l.: *C. griffithii* is sister to all other lineages and *Cliostomum* s. str. (including the type species *C. corrugatum*) is sister to the lineage *Niebla* + *Vermilacinia* with poor support. Thus, both strongly supported lineages comprising fruticose taxa are sister groups to *Cliostomum* s. str., forming an unresolved strongly supported group of three lineages.

One lineage contains two genera (*Niebla* + *Vermilacinia*) with all species but one restricted to coastal deserts of the New World subjected to oceanic fog and the other is divided into two genera, one (*Namibialina*) only with species with the same ecological requirements, but with a disjunct distribution (SW Africa) and the other (*Ramalina*) widely distributed throughout the world, with a basal species (*R. sinensis*) that is widespread throughout the Northern Hemisphere. It further includes, *inter alia*, at least two clades (the *R. bourgeana* and the *R. decipiens* gr.; Krog and Østhagen 1980; Sérusiaux et al. 2010; Pérez-Ortega et al. 2019) also associated with coastal arid habitats subjected to ocean fog, but again with a disjunct distribution as they thrive off the coasts of North Africa, mainly in the Canary Islands and Madeira archipelago.

Kistenich et al. (2018) further positioned a unique genus and species (*Cenozosia inanis*), endemic to the Atacama Desert in South America, as sister to *Cliostomum* and the other fruticose taxa now recognized in the Ramalinacae. We could not support this hypothesis with our dataset; only a rather small LSU sequence is available for that species that could complement the loci used in this study and its inclusion in our analysis resolved it at the base of the *Niebla* + *Vermilacinia* clade in an unsupported position (tree not shown). *Cenozosia inanis* was described as an epiphyte (“in ramulis dejectis prope …” Montagne 1842) and briefly presented by Bowler (1981) as follows: “The monotypic genus *Cenozosia* is a fistulose radiant of the South American *N. ceruchis* line. The thallus is either hollow or very loosely filled with medullary hyphae […]. The anatomy of the cortex is the same as that of the *N. ceruchis* aggregate […]”. The “*N. ceruchis* line”, a corticolous species in the subgenus Cylindricaria, is more appropriately referred to as the *Vermilacinia tigrina* clade. In addition, Spjut (1996) noted under *V. flaccescens* that *Cenozosia inanis* is clearly distinct for its perforated cortex and chondroid strands that crisscross the medulla. The phylogenetic position of this unique species requires further study and we expect it to be resolved at the base of the clade formed by *Vermilacinia* and *Niebla*.

### Time calibration and biogeographical patterns

The divergence between the *Ramalina* + *Namibialina* clade (RN) and the *Cliostomum* + *Vermilacinia* + *Niebla* clade (CVN) occurred before or at the beginning of the Eocene Climatic Optimum (55–50 Myrs) which was the warmest period during the Cenozoic (Zachos et al. 2001, 2008; Seton et al. 2012; Mudelsee et al. 2014). At the beginning of the subsequent climatic deterioration, the Long Term Eocene Cooling (LTEC, ca. 48 Myrs), fruticose taxa emerged:

– the RN clade evolved into two fruticose genera, *Ramalina* and *Namibialina*. Starting at c. 43 Myrs, *Ramalina* rapidly spread throughout the world, colonizing a wide range of habitats from saxicolous sea-shores to trunks and tiny branches in boreal, temperate and tropical forests. Its sister genus, *Namibialina*, radiated under more specialized ecological conditions in the coastal deserts of SW Africa, starting much later at c. 19–20 Myrs. Its diversification is thus much older than the full establishment of the cold-water upwelling system of the Benguela Current in the Late Miocene (10–7 Myrs; Heinrich et al. 2011; Rommerskirchen et al. 2011; Jung et al. 2014). The first-diverging species of *Ramalina* (*R. sinensis*) also started to diversify at that time.

– the poorly supported CVN clade divided in two taxa, one crustose (*Cliostomum* s. str.) and the other diverging at the mid Oligocene (ca. 30 Myrs) and splitting into two lineages of fruticose taxa. Therefore, the divergence of the duo *Vermilacinia* + *Niebla* is hardly younger than the establishment in northern Chile of the ecological conditions required (Oligocene to Middle Eocene; Rundel et al. 1991; Dunai et al. 2005; Le Roux 2012a, b; Gutiérrez et al. 2013; Koračin et al. 2014; Rundel et al. 2016). *Vermilacinia* diversified at 22 Myrs, that is before the Mid Miocene Climatic Optimum (MMOC) and *Niebla* started to diversify at 13 Myrs for *Niebla*, that is after the MMOC.

A similar geographical pattern is observed in three other lineages of lichenized fungi that have the same ecology, occurring on coastal rocks in fog deserts. These are: (a) the Redonographoideae which further includes two corticolous species (Lücking et al. 2013; Rivas Plata et al. 2013; Miranda-González et al. 2020), a small group of two genera and seven species (*Gymnographopsis* C.W. Dodge: three species, one in South Africa, one in Chile and one in Mexico; *Redonographa* Lücking et al.: four species, North and South America, Galapagos); (b) the genus *Santessonia* Hale and Vobis (Caliciaceae) in the Namib desert (three species: Sérusiaux and Wessels 1984) were assumed to form a monophyletic group with another set of three species from the Atacama Desert (Follmann 2006), but no molecular data are available; (c) the sister group formed in the Arthoniales by the monotypic *Combea* De Not. [*C. mollusca* (Ach.) Nyl.] endemic to the Namib and the monotypic *Dolichocarpus*R. Sant. (*D. chilensis*R. Sant.), endemic to the Atacama Desert (Ertz and Tehler 2011).

When the fruticose genera in the Ramalinaceae diverged c. 48 Myrs into the RN and CVN clades, the breakdown of Gondwana was almost complete. The Antarctic current had cooled the Antarctic continent to where all vegetation disappeared under immense glaciers (Anderson et al. 2011; Bohoyo et al. 2019). With the phylogenetic and biogeographical data available, a Southern Hemisphere origin for both clades can be argued, either in South Africa or in Patagonia and the closest Antarctica peninsula (N-W Antarctica). The only similar biogeographic pattern that we could detect in angiosperms is the Tecophilaeceae, a small family of eight genera and 27 species in the Asparagales, mainly occurring in arid ecosystems and with a disjunct distribution in California, Chile and southern and tropical mainland Africa (Buerki et al. 2013). The biogeographical scenario, inferred from phylogenetic data, assigned the most recent common ancestor (MRCA) of the family as “widespread between South America and tropical Africa” and an origin in the late Cretaceous. Empirically, we can argue that this timing and biogeographical scenario fit the data for the fruticose genera in the Ramalinaceae.

### Phylogenies of the genera *Niebla* and *Vermilacinia* and species delimitation

The number of *Niebla* species recognized by the most sophisticated BPP and STACEY statistical methods at each locality is very low. A methodology bias can influence those data, as all ITS barcodes detected at each locality could not be included in the 6-locus dataset, because of poor amplification of several loci. Nevertheless, the hypothesis of a micro-endemism pattern of allopatric species cannot be ruled out and variation in space and time of fog conditions may provide support for this scenario. Indeed, their restricted geographical range and their radiation at c. 22 Myrs for *Vermilacinia* and at ca. 13 Myrs for *Niebla* clearly point to the paramount importance of climate change since the Miocene (Spjut 1996; Cerling et al. 1997; Herbert et al. 2016).

Pacific coastal fog relates to seasonal high/low pressure areas that impact the strength of an inversion layer and temperature of the California Current (for the Northern Hemisphere) or the Chile-Peru Current (for the Southern Hemisphere), location of upwelling water caused by wind and Coriolis force diverting water away from shore (“Ekman transport”) and topography, including offshore continental slope and shelf width (Zaytsev et al. 2003). As surface water moves away from the coast, colder water upwells, chilling the overlying humid marine air to saturation – creating fog. Land heated during day above sea temperatures lowers pressure causing fog to drift landward. Seasonal high pressure intensifies fog against the windward side of coastal ranges. The result is that fog is patchy, varying in its intensity and its flow inland (Rundel et al. 1991; Cartapanis et al. 2011). Further, several studies could highlight the instability (in surface affected and continuity) of fog over the last millions of years (Ortiz et al. 1997; Heusser 1998; Herbert et al. 2001; Snyder et al. 2003; Addison et al. 2018). We can therefore postulate that intermittence of fog, both in time and area affected, may provide a sufficient driving force for active speciation. Retreat of fog along a mountainous coastline with various bays and inlets would create localized habitats for *Niebla* and *Vermilacinia*, such as what we see today along the Pacific Coast. During times when fog becomes more continuous along the coast, the previously isolated populations also expand and come into contact.

However, several alternative patterns can substantiate or dispute the hypothesis of micro-endemism such as incomplete lineage sorting or hybridization (Buschbom and Mueller 2005; Stewart et al. 2014) as recently suggested for the genus *Rhizoplaca* Zopf (Keuler et al. 2020). However, micro-endemism within a single radiation has been demonstrated for a lineage of *Sticta* in the MIOI (Madagascar and Indian Ocean Islands; Simon et al. 2018) and might represent a widespread pattern in lichenized fungi, as strongly suggested in several other studies (Sérusiaux et al. 2011; Lücking et al. 2014; Dal Forno et al. 2017; Lücking et al. 2017b).

### Insights into the species diversity of *Niebla* and *Vermilacinia*

Despite the incongruence of morphological species with phylogenetic reconstructions in *Niebla*, geographical lineages are apparent such as the *Niebla homalea* group (Fig. 7), characterized by a relatively thick cortex with a solid medulla, occurring largely in the California Floristic Province in contrast to lineages with a relatively thinner cortex and more fistulose medulla, occurring in the NVD. Additionally, distinct lineages for *Niebla homalea* in Northern California might relate to movement of the Pacific Plate relative to the North American Plate during the past 2 Myrs. A similar pattern is detected in *Ramalina menziesii*: the oldest of geographically defined lineages for this species ranging from the Pacific Northwest to the Vizcaíno deserts, was recognized as a unique lineage in the Vizcaíno deserts, having been isolated for perhaps 1–2 Myrs (Sork and Werth 2014).

None of the ß-depsidones-producing species as Spjut (1996) delimited by morphological, chemical and ecological characters could be recognized. The main reason for this discrepancy is that Spjut (1996) applied the ß-depsidone metabolite as a discriminant character: protocetraric acid for *N. pulchribarbara*, hypoprotocetraric acid for *N. brachyura* and *N. spatulata* and salazinic acid for the six other species (*N. arenaria*, *N. effusa*, *N. flabellata*, *N. josecuervoi*, *N. limicola* and *N. marinii*) and *N. homaleoides* for thalli lacking ß-depsidones. All are related by the absence of triterpenes. Examples of the discrepancy between the work of Spjut (1996) and the species delimitation produced here with molecular data are (a) *N. brachyura* represented by four accessions that resolved into three different species by the BPP method and (b) the basal clade with only *N. flabellata* or *N. spatulata*, recognized as a single species by BPP, but as two by Spjut (1996) who used the two different metabolites to distinguish these two species (salazinic acid for *N. flabellata* and hypoprotocetraric acid for *N. spatulata*). This example is mainly applicable to collections from the SVD where thalli of the two depsidone chemotypes consistently occurred together at many sites, whereas the type specimen for *N. flabellata* (Spjut and Marin 9073H5, US) was collected in close association with the divaricatic acid-producing *N. caespitosa* (Spjut and Marin 9073C, US) in the southern NVD and the two species could only be distinguished by chemistry. *Niebla flabellata* is thus supported in the NVD by chemistry from similar morphs (*N. caespitosa* with divaricatic acid and *N. lobulata* with sekikaic acid), but not by morphology from the related ß-depsidone species. Thus, these species are still unresolved from a phylogenetic perspective.

The taxonomy proposed by Spjut (1996) relies on the combination of chemical and morphological characters. Although his *Niebla* taxonomy is not corroborated by molecular inferences and statistical speciation methods, that of saxicolous *Vermilacinia* is corroborated and this likely is to be found in South American species.

Spjut (1996) recognized two subgenera within *Vermilacinia*: subgenus Vermilacinia with the saxicolous *V. combeoides* as type species and subgenus Cylindricaria with the corticolous *V. corrugata* as type species. These two subgenera are recovered and can be confirmed if the saxicolous *V. combeoides* and *V. laevigata* are treated separately from the corticolous species all included in the subgenus Vermilacinia. This would require recognizing a third subgenus for the majority of saxicolous species – the alternative option would be not to recognize any subgenera.

Although future inclusion of data for species occurring in South America may bring in new structure for the *Vermilacinia* phylogenetic tree, it is nevertheless interesting to highlight that, for the Northern Hemisphere Pacific coasts, the corticolous habitat is a more recent autapomorphy. Contrarily to saxicolous species whose species delimitation is resolved, the terminal and, thus, most recent corticolous *Vermilacinia* are taxonomically problematic. Only the two oldest clades are fully resolved: *V. cerebra* is resolved as a monophyletic group and the sorediate populations are resolved into two different species (*V. cephalota* for populations from USA/California and a yet undescribed species for those from Baja California). All others are resolved into two strongly supported clades: (1) one without any black bands can be attributed to *V. corrugata*, a species with a corrugated cortical surface that is deficient in terpenes except zeorin and occasionally T3 (Spjut 1996); it occurs in the Channnel Islands and abundantly on shrubs in Baja California for nearly the entire length of the peninsula along the fringe of the coastal fog – to at least the vicinity of Isla Magdalena in Baja California Sur; (2) the second has black bands or irregular spots, around the lobes, the pycnidia or the apothecial discs; Spjut (1996) recognized six species in that morphologically (including production of punctiform soralia) and chemically variable group: *V. howei*, *V. leopardina*, *V. nylanderi*, *V. zebrina* plus *V. leonis* and *V. tigrina* (not represented in our dataset). Three species are delimited by the BPP analysis and a single one by STACEY. There is hardly any matching between the species delimited by Spjut (1996) in the “black bands” lineage. The accession from Namibia is not recognized as a different species, although an autapomorphic substitution in ITS2 is easily detected; we can therefore assume that this disjunct population is the result of a single, very recent colonization event. As for *Niebla*, more samples and data are needed to resolve the taxonomy of this genus.

As for the saxicolous and terricolous species of *Vermilacinia*, the taxonomical weight given to chemistry by Spjut (1996) for the recognition of species could find support in our dataset. The TLC data of the four accessions of *V. combeoides*, *V. laevigata* and *V. procera* from USA/California included in Sérusiaux et al. (2010) are “triterpenes” without any other information; one can thus consider that this statement is coherent with the typical assemblage for those species: zeorin, T3 and [-]-16α-hydroxykaurane. Indeed, this “T3 triterpene assemblage” is present throughout the clade and represents the plesiomorphic chemical assemblage, as is also the case for the related saxicolous species in South America. The T1 + T2 assemblage, with zeorin and [-]-16α-hydroxykaurane, is detected in two lineages, one with the newly described *V. lacunosa* and the second with *V. ligulata* and *V. reptilioderma*. Therefore, species with the T1 + T2 assemblage include *V. lacunosa* and *V. rosei* in the SVD and *V. johncassadyi* and *V. ligulata* in the NVD extending to just north of Punta Canoas on Mesa Camacho; only *V. reptilioderma*, the last T1 + T2 species, being present throughout that range. *Vermilacinia johncassadyi* and *V. rosei* also occur on the nearby island Cedros. All these species have a restricted range and we can confidently assume a micro-endemism pattern. Finally, the newly described *Vermilacinia lacunosa* is unique amongst the saxicolous *Vermilacinia* in the Northern Hemisphere for containing methyl 3,5-dichlorolecanorate (= tumidulin), otherwise known in the two South American species *V. flaccescens* and *V. granulans*. *Vermilacinia lacunosa* was recovered as a unique lineage within the phylogenetic tree, which reinforces the taxonomic significance of the secondary metabolites in the genus.

Spjut (1996) reported two saxicolous species to reach Tierra del Fuego in Argentina (*V. ceruchis* and *V. combeoides*). He now suspects that E. Tuckerman (1817–1886) mounted California specimens of *V. combeoides* on the same herbarium sheet close by specimens reportedly collected from Callao, Peru and Coquimbo, Chile for making comparisons. Spjut (1996) treated the Peru and Chile saxicolous specimens as variants of the terricolous *V. ceruchis*; however, based on our study of saxicolous *Vermilacinia* employing molecular data, they almost certainly are distinct from *V. ceruchis* and, therefore, we refer to them as V. aff. ceruchis and V. aff. combeoides; the former differs in having inflated branches with many lateral apothecia along a branch, the latter differs by the subterminal apothecia instead of strictly terminal apothecia. Vermilacinia cf. flaccescens has also been reported from Patagonia (Yang et al. 2018).

Delimiting species boundaries and establishing robust taxonomies for these two genera are challenging tasks that need more samples and data and more sophisticated techniques for species delimitation (Altermann et al. 2014; Magain et al. 2017; Grewe et al. 2018).

### Phylogeny of the genus *Namibialina* (Fig. 9)

Two typical species occurring in the coastal deserts of SW Africa are here assigned to the new genus *Namibialina*: “*Ramalina*” *melanothrix* recovered as a single taxon and “*Ramalina angulosa*” (Wirth 2010a, b), recovered as a paraphyletic assemblage of three species. Although there are no clear-cut morphological, anatomical or chemical autapomorphies, we choose to recognize a new genus, sister to *Ramalina*, as its divergence time (from *Ramalina*) is dated at c. 48 Myrs, almost simultaneously to the emergence of the MRCA (Most Recent Common Ancestor) of the CVN clade comprising three genera, clearly different from one another on morphological and chemical characters.

The taxonomical status of *Ramalina melanothrix* is straightforward, while that of *R. angulosa* is not. First, the type material was not available for study and its nomenclature is confusing (see § Taxonomy and Nomenclature). Further, we suspect this morphotype is widespread from the coastal desert of Namibia (of which only a short portion ca. 120 km long was sampled; see above under Material and methods) down to the Cape area where the type was collected. A much higher diversity is expected as three supported and sympatric species are easily detected in our material, differing from one another by the branching pattern including capillary cilia, the cortex surface, several characters associated with the cartilaginous strands under the cortex and production or not of apothecia.

### Phylogeny of the genus *Ramalina* (Fig. 9)

**Figure 10.**
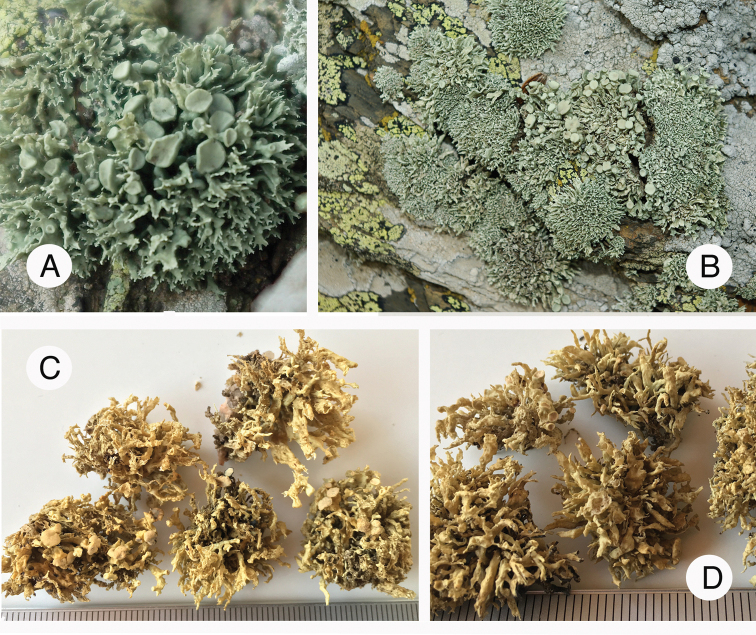
*Ramalina breviuscula* aggr **A, B***R. breviuscula*, type locality in France, eastern Pyrenees (photographs taken in the field) **C, D***Ramalina* sp. 2 (France, Corsica). Scale: 1 mm (**C, D**). Photographs by E. Sérusiaux.

**Figure 11.**
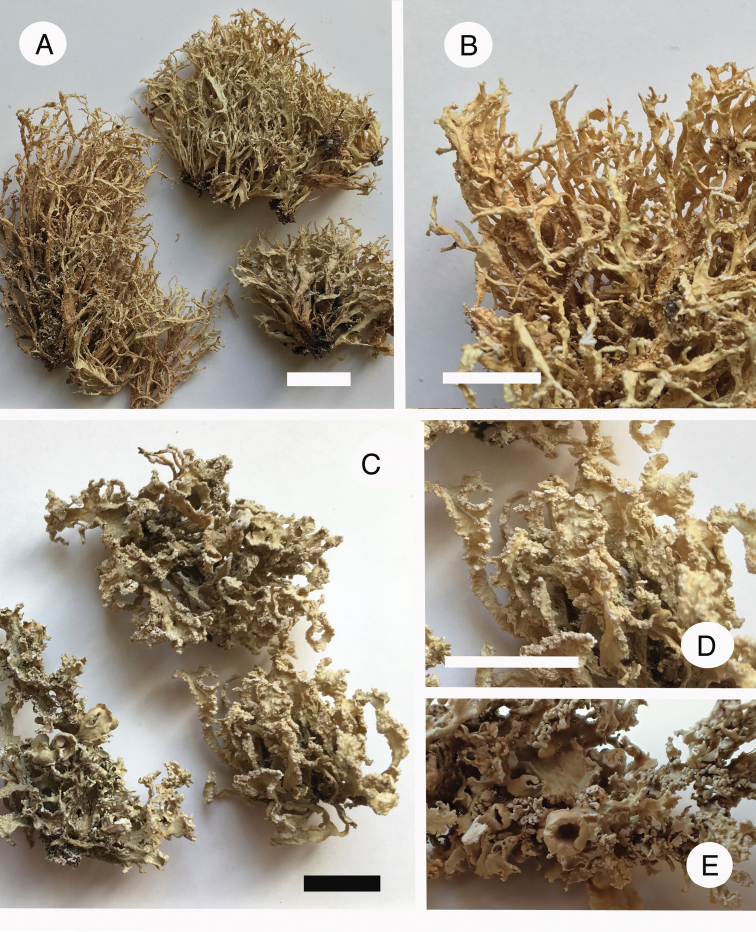
Species in *Ramalina***A, B***R. krogiae* (holotype) **C–E***R. requienii* (France, Corsica). Scale: 1 cm (**A–D**); scale in **E** identical to **D**. Photographs by E. Sérusiaux.

The subcosmopolitan genus *Ramalina* exhibits an impressive thallus variation as it ranges from luxuriant pendulous thalli up to several metres long, hanging down from branches of tall trees (such as in *R. hoehneliana* and *R. menziesii*) to unattached and almost pulverulent thalli hidden in rock crevices. Further, the thallus branches are very diverse as they can be terete or markedly compressed and applanate, hollow or solid, usually with a chondroid tissue and lax medulla; with or without fenestrations, reticulate ridges, pseudocyphellae, laminal striae, tiny lateral hooked fibrils, cilia, soralia; pycnidia with a black ostiole or not. The number of species is expected to be 230 (Lücking et al. 2017a) and 50 are represented in our study, including one here newly described and three that cannot be assigned to a validly published epithet (Figs 2, 6, 9–11).

All species lacking secondary metabolites, except for usnic acid in the cortex, are resolved at the base of the tree. However, these species do not form a single lineage and are intermingled in paraphyletic lineages with two species (*R. crispans* and *R. rubrotincta*) that do produce secondary metabolites.

Although the sampling of species included in this study may not be representative of the variation throughout the genus, chemistry-based branches supporting several species are numerous with, *inter alia*: (1) two clades characterized by the production of ß-depsidones: the *farinacea* and the *cribrosa* clade, with one anomaly in *R. subgeniculata* whose thalli produce divaricatic acid, the ß-depsidone salazinic acid being restricted to the apothecia; (2) two clades characterized by the production of evernic acid: the *breviuscula* and the *fastigiata* clade (the latter with one exception with sp. 1 which produces several ß-depsidones); (3) the *bourgeana* clade characterized by the production of bourgeanic acid, with the exception of *R. webbii*, which does not produce that metabolite; and (4) two supported clades with all species producing divaricatic acid: the *canariensis*-clade and the *huei*-clade.

Strongly supported clades, however, can have a diverse chemistry, such as the *decipiens* clade, endemic to the Canary Islands and the Madeira archipelago (including Porto Santo), which have species producing either ß-depsidones or depsides. Therefore, worldwide sampling is needed to further evaluate secondary chemistry to give support for deep node segregation, such as in other macrolichen genera (*Xanthoparmelia pulla*-group: De Paz et al. 2012; *Usnea cornuta*-group: Gerlach et al. 2019; *Cetrelia*: Mark et al. 2019). However, the model of chemical characters as support of evolutionary nodes is not universal as demonstrated by the *Bryoria fuscescens* group (Boluda et al. 2019) and *Thamnolia* (Onuț-Brännström et al. 2017).

**Figure 12.**
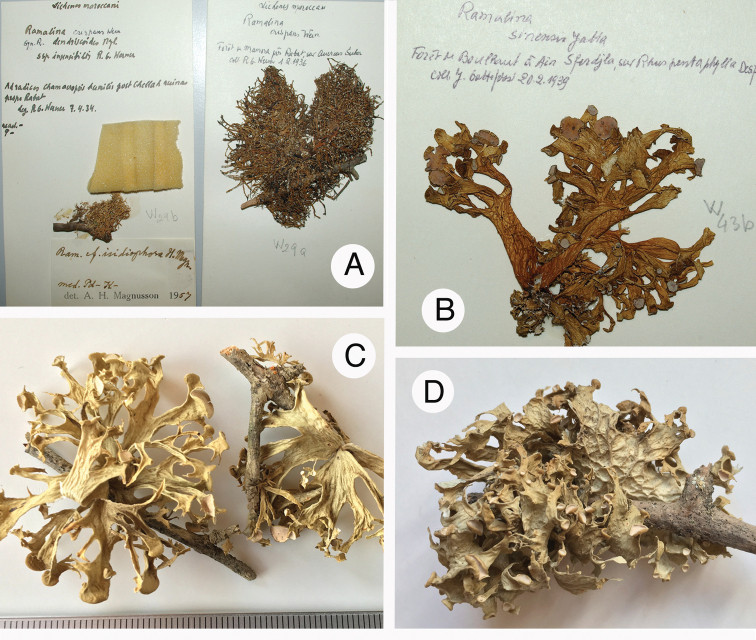
Species in *Ramalina***A***R. crispans* (holotype: left-hand specimen; other specimen from Morocco: right-hand) **B***R. lusitanica* (Morocco, leg. J. Gattefosse, BC) **C, D***R. lusitanica* (Italy/Sardinia, accession LG DNA 1525). Scale: 1 mm (**C**). Photographs by E. Sérusiaux.

Other interesting results include: (1) *R. requienii*, a rather common saxicolous species in the Mediterranean region and in the Canary Islands, from sea-level to montane regions, here resolved into two different species: one restricted to the Canary Islands and the Madeira archipelago and the other to the Mediterranean region; as the type was collected in France/Corsica (Krog and Østhagen 1980) and as no available epithet could be assigned to this species, the new *Ramalina krogiae* is described below (Fig. 11). (2) Confirmation of sibling species (Culberson 1986) with *R. inaequalis* and *R. tingitana*, here confirmed as two sympatric species (Fig. 2), sister to each other, thriving in the same habitat on rocky sea-shores in the western Mediterranean region. (3) The *R. farinacea* complex is here resolved into two clades: one includes the typically sorediate corticolous species *R. farinacea* and the saxicolous *R. subfarinacea*; the same ecomorphotype, however, is also resolved in the second clade, further comprising the non-sorediate and fertile *R. implectens*. Therefore, the classical taxonomy of this species complex (Aptroot and Schumm 2008: *R. implectens*, fertile; *R. farinacea*, sorediate and corticolous; *R. subfarinacea*, sorediate to isidiate and usually saxicolous) is not supported by molecular inferences. Further, both *subfarinacea* ecomorphotypes are sympatric along the northern coasts of Norway. These results are consistent with the library of DNA barcodes for Nordic countries (Marthinsen et al. 2019). (4) Two very variable groups, both producing evernic acid and not resolved as sister taxa: one includes the corticolous *R. fastigiata*, almost always fertile, usually forming densely tufted and richly branched, pulvinate thalli, but quite variable in size of thalli and lobes and especially in the spur-shaped lobe at the base of apothecia; and the saxicolous *R. breviuscula*, with fertile, densely tufted and branched, pulvinate thalli. Two morphotypes can be distinguished within the *breviuscula* lineage (Fig. 10): the first corresponds to material sampled at the type locality, widespread from sea-level outcrops to exposed ones at higher elevations (compact pulvinate thalli with young lobes typically dichotomously branched at their apices; lobes becoming tubular, remaining rather regular and not pitted); and the second one found only in Corsica at 930–940 m in a forest context (less compacted thalli with young lobes simple or not regularly branched and mature lobes irregular, remaining tubular, but many irregular holes) is here recognized as a separate species, so far unnamed. (5) Finally, accessions of fertile, epiphytic and divaricatic acid-producing populations from the western Mediterranean region are shown to be a well-delimited species, for which the epithet *lusitanica* is re-appropriated (Fig. 12).

As expected, detailed morphological and chemical studies supported by molecular inferences end up with taxonomical adjustments, descriptions of new taxa or resurrection of old epithets. An interesting case is the basal species *R. sinensis* which started its diversification c. 19 Myrs (Early Miocene) and produced four different lineages that might be worthy of recognition at species level: the earliest divergence isolated an accession from Western North America; the second (16–17 Myrs) isolated an accession from Taiwan (East Asia); the third one (9.5 Myrs) separated two accessions of the Caucasus region s.l. (Armenia and Iran) from two others from central Europe (Switzerland) and central Canada (Alberta).

## Conclusions

The Ramalinaceae include two strongly supported lineages of fruticose thalli: (1) *Ramalina* and *Namibialina* (gen. nov.) and (2) *Vermilacinia* and *Niebla*. Both form an unresolved clade with the crustose genus *Cliostomum*, excluding the well-known *C. griffithii* which is resolved in its own clade, sister to all others. The relationship of the monotypic *Cenozosia*, endemic to the Atacama Desert, remains to be determined.

Three of the fruticose genera are endemic to coastal fog deserts, *Namibialina* in SW Africa, *Vermilacinia* along the Pacific coasts in South America and North America, with a recent dispersal of a sorediate epiphytic species, *V. zebrina*, to Namibia and *Niebla* only in North America. All three genera are actively speciating and need further work to thoroughly address their taxonomy and diversification patterns.

The taxonomy of *Niebla* and *Vermilacinia* proposed by Spjut (1996) is largely confirmed, especially for the distinction between *Niebla* and *Vermilacinia* and the *a priori* assumption that all characters (secondary metabolites and morphology of thalli) can be employed for the delineation of species. An example is provided by *Niebla* as the deep nodes of its evolutionary structure are supported only by chemical characters. For *Niebla*, a larger sampling exercise, including all type localities, is needed to resolve its taxonomy at species level as the current species delimitation (Spjut 1996) is not supported by molecular data and inferences. Further, the model of micro-endemism of allopatric species cannot be ruled out at this stage. This model also finds strong support in the saxicolous species of *Vermilacinia*, whose phylogeny and species delimitation are fully resolved and includes four species here described as new for science. The corticolous species of *Vermilacinia* are partly resolved, the two most recent *V. corrugata*-clade and the “black-banded species”-clade need further work.

Our study confirms that the genus *Ramalina*, with a subcosmopolitan distribution and colonizing so many different habitats, is indeed a monophyletic group, based on 50 identified species (plus three without a specific epithet) represented for an estimated total of 230 species. The topology shown by the evolutionary tree confirms that all species that do not produce secondary metabolites (other than usnic acid produced in the cortex) are resolved at the base of the tree, but do not form a monophyletic group. Several clades supporting several species correspond to the production of peculiar secondary metabolites, but none of these constitutes an autapomorphy for a well-supported monophyletic clade, with the exception of bourgeanic acid which is unique to the *bourgeana*-clade. The data largely confirmed the present taxonomy with several corrections needed, the most interesting one being the diversity of the largely distributed basal species *R. sinensis* which started to diversify ca. 19 Myrs ago.

## Taxonomy and nomenclature

We here provide several notes on the taxonomy and nomenclature of a few taxa discussed in this paper, including re-assessment of three epithets and description of a new genus and five new species.

### 1 *Namibialina*: a new genus for “*Trichoramalina melanothrix*”

#### Namibialina

Taxon classificationFungiLecanoralesRamalinaceae

Spjut & Sérus. gen. nov.

297D8B2F-DCA3-599D-A1C0-BBBD215E2F6B

833602

= Ramalina melanothrix Laurer, Syn. Meth. Lich. 1(2): 290, tab. VIII, fig. 26, 1860
= Trichoramalina melanothrix (Laurer) Rundel and Bowler, The Bryologist 77(2): 194, 1974
= Niebla melanothrix (Laurer) Kistenich, Timdal, Bendiksby, S. Ekman, Taxon 67(5): 893, 2018

##### Type species.

*Namibialina melanothrix* (Laurer) Spjut & Sérus., comb. nov.

##### Description.

Thallus shrubby, usually arising from a single holdfast, stiff or flexuose, several cm in height when epiphytic or, saxicolous or developing terricolous shrubby cushions up to ca. 10–15 cm in diam. and 2–6 cm in height (fig. 2 in Wirth 2010b), typically pale green or yellowish-green. Branches usually dividing, regularly or not, dichotomous or not, apices usually with capillaceous or blackish terminal hairs; main branches terete-angular or flattened, distinctly canaliculate, because of well-developed longitudinal strands of cartilagineous tissue. Lateral branches and spinules sometimes present; typical isidia or soralia never observed. Medulla arachnoid, very thin. Cortex 2-layered, but not always typical. Apothecia present or absent, disciform, terminal or marginal and sometimes typically orientated perpendicular to the branch. Ascospores ellipsoid, straight or slightly bent, 1-septate, ca. 10–20 × 5–8 µm. Pycnidia sometimes abundant, with black ostioles. Secondary metabolites: usnic ac. in the cortex.

##### Remarks.

The type species of *Trichoramalina* (Rundel and Bowler 1974), characterized by marginal black hairs, is *T. crinita* (Tuck.) Rundel and Bowler, a species endemic to the coastal areas of California and Baja California (Fig. 3). Kistenich et al. (2018) could demonstrate that it belongs to *Ramalina* and our own accessions support this phylogenetical position. The other species with black hairs (“*Trichoramalina*” *melanothrix*) is shown to have an isolated phylogenetic position and forms together with *Ramalina angulosa* (sensu Wirth 2010b) the sister group to all other accessions of *Ramalina* s. str. Although there are no clear-cut morphological, anatomical or chemical autapomorphies, a new genus is here introduced for this lineage endemic to SW Africa (Namibia and South Africa). Indeed, it diverged from *Ramalina* c. 48 Myrs ago early in the Eocene, almost simultaneously with the emergence of the MRCA (Most Recent Common Ancestor) of the CVN clade comprising three genera, clearly different from one another on morphological and chemical characters. Further, the basal species of *Ramalina* s. str. is *R. sinensis*, an epiphytic species widespread throughout the Northern Hemisphere, a complete contrast with *Namibialina* and that also started to diversify at c. 20 Myrs.

Two described species are here assigned to the new genus *Namibialina*: *Ramalina melanothrix* recovered as a single taxon and “*Ramalina angulosa*” (Lalley and Viles 2005; Wirth 2010a, b), recovered as a paraphyletic assemblage of three species, with *R. melanothrix* nested amongst them. All accessions available for DNA extraction and analysis were collected in Namibia, along ca. 120 km of coastline North of Swakopmund. Therefore, we strongly suspect that a complex assemblage is hidden under these epithets, most probably much more diverse along the coastal deserts of SW Africa, from the southern part of Angola down to the Cape of Good Hope. It is currently under study.

Strands of cartilaginous tissue develop longitudinally under the cortex in all species of *Namibialina* and in several species of *Ramalina* resolved at the base of the tree, such as *R. sinensis* and *R. celastri*. In *Namibialina*, chrondroid stands are “attached to the cortex”, not isolated in the medulla, the medulla is arachnoid, very thin and supported by a 2-layered cortex, sometimes the external layer indistinct (Rundel and Bowler 1974, Bowler 1981). Another shared plesiomorphy is the lack of secondary metabolites (except for usnic acid in the cortex) in *Namibialina* and in several basal species in *Ramalina* (*R. celastri*, *R. fraxinea*, *R. hoehneliana*, *R. sinensis* and others). However, Wirth (2010b) mentioned several secondary compounds in “*Ramalina angulosa*”: ± bourgeanic, ± sekikaic, ± 4’-O-demethylsekikaic and ± salazinic acid.

The nomenclature of the above-mentioned epithets can be summarized as follows: Theodor Magnus Fries (Flora 44: 411, 1861) clearly recognized *Ramalina melanothrix* as different from what had been assigned to *R. angulosa*. He referred to an annotation of Johann Friederich Laurer using that epithet for a collection made by Johann Franz Drège “in Africa meridionali”. J.F. Drège is a very famous plant collector in the Cape area (Gunn and Codd 1981) and we can thus assume his specimen was collected in that area. However, in the same section, Th. Fries thoroughly described *Ramalina capensis*, with two varieties: “Formae duae, quas amplectitur, nominandae: α *angulosa* Laur. […] ramis apicalibus concoloribus et ß *melanothrix* Laur. […] apicibus thalli […] nigricantibus“.

Further, a collection assumed to be a type from the Royal Botanic Garden Edinburgh E is available at: https://plants.jstor.org/stable/viewer/10.5555/al.ap.specimen.e00465255.

There is no doubt that this collection belongs to the “*angulosa*” assemblage. It has a label indicating it was collected in Tahiti, but annotations by B.J. Coppins (Feb. 1976) reads as “This is a Drège specimen from South Africa”.

### 2 *Niebla*

**Type species of *Niebla***

There has been a lot of debate regarding the type species of this genus (Krog and Østhagen 1980; Sérusiaux et al. 2010). The genus name is a substitute name for *Desmazieria*, a homonym created by Camille Montagne in 1852 who recognized only one species in the genus (*D. homalea*). However, the name *Desmaziera*, although not spelled exactly the same, but nevertheless considered to be the same (homonym), had been given to a genus of grass (Poaceae) by Barthélemy Dumortier in 1822. The earliest name is the one that must be retained unless the later name is conserved, according to the ICN. Additionally, the type species name for *Niebla* is *Niebla homalea*, based on the name given to the one and only species that had been recognized by Montagne for the genus, not *Niebla ceruchis* as stated in Sérusiaux et al. (2010).

**Lectotypification for *Niebla procera***

The holotype of *Niebla procera* Rundel & Bowler at ASU is represented by two specimens shown in Bowler et al. (1994, fig. 4) in black and white and a mirror image is available in colour in the CNALH website (https://lichenportal.org). The CNALH image clearly shows that two specimens are involved. We identify *Vermilacinia procera* on the left and Vermilacinia cf. paleoderma on the right; therefore, we designate the left-hand specimen as the lectotype of “*Niebla procera*”.

The lichen metabolites reported for *N. procera* in Bowler et al. (1994) are “[-]-16α-hydroxykaurane, ± zeorin, ± salazinic acid, terpenes, fatty acids, ± usnic acid”. A fragment of the specimen on the right may have been removed for TLC (the upper part of a thallus branch appears scraped just below the tip). The morphological description given in Bowler et al. (1994) generally agrees with the specimen on the left. Both species have similar chemistry (Spjut 1996). Additionally, Bowler et al. (1994) cited specimens of “*Niebla procera*” mostly from islands, five of the Channel Islands, one from Isla Guadalupe (as *V. paleoderma* in Spjut 1996), one at the north end of Isla Cedros and from one location on the Vizcaíno Peninsula, 3.5 km W of Mexico Hwy 1 along the road to Punta Abreojos (generally *V. paleoderma* in Spjut 1996). They did not cite specimens from the NVD. They also recognized another species in the Channel Islands and adjacent coastal California (Ventura and Los Angeles counties), “*Niebla polymorpha*” [= *Vermilacinia polymorpha*, type from Santa Catalina Island, also shown present in the chaparral region of Baja California without reference to specimens (Bowler et al. 1994)]. We consider *V. procera* to occur mostly on the mainland, Baja California from near San Quintín to Marin County, California; Spjut (1996) also cited two specimens from the Channel Islands, one from Santa Catalina Island collected by Howe and one from Santa Cruz Island collected by C. Bratt. Saxicolous species of *Vermilacinia* south of Bahía de San Quintín, including Isla Guadalupe (*Palmer* s.n., US!), belong to the *V. paleoderma* group, which includes new species described in this paper; however, this group might also be present near Punta Escarpada in the NVD as suggested in Spjut (1996).

### 3 *Ramalina*

#### Ramalina crispans

Taxon classificationFungiLecanoralesRamalinaceae

R.G. Werner, Scientific Annals of the School of Agriculture and Forestry, Aristotelian University, Thessaloniki IH’-B: 1 (1977).

522F8563-1760-5E47-98B7-9460D28711EB

131128

Fig. 12A, right-hand specimen

##### Type.

Morocco – Original publication reads “ad corticem *Quercus suberus* L. in Mamora silva prope Rabat” (Werner 1977: 1); label reads as “Forêt de Mamora près Rabat, sur *Quercus suber*”, 01.02.1936 s.n. (BC! – holotype).

##### Description.

Thallus epiphytic, almost always on tiny branches, shrubby, usually rather small (less than 2–5 cm long), formed of densely intricate branches that are solid, slightly flattened and irregularly thickened; soralia conspicuous albeit quite small, granular, often with small fibrils; pseudocyphellae common, ellipsioid or linear; apothecia and pycnidia unknown.

##### Chemistry.

Acids in the sekikaic aggregate with sekikaic and homosekikaic acids as the main compounds detected; usnic acid.

##### Distribution and ecology.

Mediterranean area and Cabo Verde archipelago; assumed to be present in the Canary Islands, the Madeira archipelago and the Azores archipelago; on branches, including twigs, never found on trunks, in open shrubland.

##### Remarks.

This species was described as “spec. nova ad interim”, an unclear status that could be questioned under the ICN code art. 34.1 (b). Nevertheless, we adopt it pending further nomenclatural clarification. Following Aptroot and Schumm (2008), this species would key out as *R. peruviana* Ach. This epithet is used for any densely branched, sorediate *Ramalina* producing sekikaic acid throughout the world (Swinscow and Krog 1988 for tropical East Africa; Aptroot and Bungartz 2007 for the Galapagos Islands; Galloway 2007 for New Zealand; Aptroot and Schumm 2008 for North Atlantic Islands; Oh et al. 2014 for China). *Ramalina peruviana* Ach. is a validly published epithet and the original publication states “Habitat in Peruvia in confortito crefens …”. No material from Peru or surrounding countries that could match the original description was available for DNA analysis. Therefore, we choose to use the epithet introduced by R. G. Werner for Mediterranean material of corticolous *Ramalina farinacea* look-alikes and producing sekikaic acid. Our dataset shows that accession from Cape Verde and Greece are identical; we assume that reports from the archipelagoes of the Azores, Canary Islands and Madeira belong to the same species.

##### Additional specimens examined.

Cabo Verde archipelago – São Vicente, Monte Verde; assumed at 16°52.2'N, 024°56.0'W; alt. ca. 730 m; 04.2008; J. Lambinon 08/20 leg.; on shrubs (LG DNA 428); [DNA: GU726358 (LSU), GU827317 (ITS), MN757015 (RPB1), MN757230 (RPB2)]. Greece – Dodecanese, Karpathos Is., top of Mt Hagios Elias; 35°43.6'N, 027°10.5'E; alt. 710 m; 07.2007; H. Sipman & Th. Raus 56261 leg.; on *Erica* dwarf shrubs (B, LG DNA 1553); [DNA: MN811427 (ITS)]. Morocco – “Chellak ruinas prope Rabat”; 07.04.1934; R.G. Werner s.n.; “ad radices *Chamaropsis humilis*” (BC). TLC for both collections from Morocco (incl. type) by Amami N., Arroyo & Seriñá, annotation of May 2002.

#### Ramalina krogiae

Taxon classificationFungiLecanoralesRamalinaceae

Guissard & Sérus. sp. nov.

D747FDF6-05E3-52D7-931B-689B6F2ACD89

833605

Fig. 11A, B

##### Diagnosis.

*Ramalina krogiae* is recognized by its saxicolous habitat, ascending, 1–2 dichotomous branched, rigid lobes, producing abundant granules, but no genuine soralia, nor apothecia, producing divaricatic acid and endemic to the Canary Islands and Madeira archipelagoes

##### Type.

Spain – Canary Islands, La Gomera, W of Arure, N of Ermitago de Santo; 28°08.10'N, 017°19.23'W; alt. 825–830 m; 04.2009; E. Sérusiaux s.n. leg; subvertical outcrops in open matorrales; (LG DNA 666! – holotype; TFMC! – isotype) [TLC: divaricatic and usnic acid; DNA: MN811446 (LSU), MN811250 (ITS), MN757049 (RPB1), MN757257 (RPB2)]

##### Description.

Thallus saxicolous, ascending, usually rigid, when well-developed up to 3–4 cm high, usually with 1–2 dichotomous branching in the upper half of the branch, 3–4 mm large at most, usually less, branches contorted or irregularly twisted, small dissected and elongated lobes (small laciniae) usually present, together with tiny rounded or irregularly shaped granules, these granules sometimes abundant; cortex locally and usually irregularly broken off, but no typical production of soralia observed. Apothecia rare to abundant, lateral, disc usually strongly concave (“wide open” apothecia rarely seen) with a scrobiculate outer cortex, margins of the disc usually with cortex interruption and production of tiny granules. Ascospores straight or slightly concave, 1-septate, 9–13 × 4–5 µm. Pycnidia not found.

##### Chemistry.

Divaricatic and usnic acid, triterpenoids.

##### Distribution and ecology.

On exposed rocks at low elevation, in the Canary Islands and the Madeira archipelago.

##### Etymology.

Epithet chosen after our most distinguished colleague Prof. Hildur Krog (1922–2014), author, inter alia, of a remarkable and detailed revision of the genus *Ramalina* in the Canary Islands (Krog and Østhagen 1980).

##### Remarks.

Besides its distinct geographical range (Mediterranean region vs. Canary Islands and Madeira archipelago), the morphologically and chemically similar *R. requienii* can be distinguished by its usually larger lobes with sublinear pseudocyphellae and especially the lobes extremities rather typically labriform, with a lower surface with large patches of disrupted cortex and production of coarse soralia. In typical populations of *R. krogiae*, no such labriform and slightly, but distinctly, expanded lobes are formed and tiny granules produced on or around the rather large cortex interruptions at the lobe extremities are not observed. Therefore, the distinct phylogenetic relationships of *R. krogiae*, as well as its disjunct distribution, are complemented by morphological features, which albeit rather cryptic can be easily detected with some taxonomic expertise.

##### Additional specimens examined.

Portugal – Madeira, Ponta de São Lourenco; 32°44'N, 16°40'W; alt. 150 m; 05.04.2007; D. Ertz 10520 leg.; rock outcrop near the sea; (BR, LG DNA 431); [DNA: GU726360 (LSU), GU827319 (ITS), MN757017 (RPB1), MN757232 (RPB2)]. Portugal – Porto Santo, between Pico de Castelo and la Capela de Nossa Senhora da Graça; 33°04.41'N, 016°19.37'W; alt. 200–220 m; 04.2007; M. Dewald, A. Hambuckers & E. Sérusiaux leg.; outcrops in pastures; (LG DNA 459); [DNA: MN811437 (LSU), MN811241 (ITS), MN757040 (RPB1)].

#### Ramalina lusitanica

Taxon classificationFungiLecanoralesRamalinaceae

H. Magn., Bot. Notiser 109: 149 (1956)

5D6C6869-CE1E-59C6-8F43-4844C1BB4EF4

369922

Fig. 12B–D

##### Type.

Portugal – Estramadura, Serra da Arrabida, between Setubal and Torre de Outao; 01.05.1931; G. Degelius leg.; on trees (UPS L-78721 ! – holotype).

##### Description.

Thallus corticolous, usually on branchlets, erect or rarely partly pendulous, up to 4–5 cm in diam., with a fan-shaped appearance (with terminal apothecia) or a small-cushion one; lobes divided dichotomously or trichotomously, rather stiff, flat or slightly concave, up to 3–4 mm large just before the first division; upper surface slightly grooved or channelled, often longitudinally ridged; lower surface undulating, distinctly scrobiculate on well-developed lobes. Apothecia usually present and abundant, terminal or lateral on young lobes, up to 4–5 mm in diam., usually 2–3 mm, disc concave, with no spur or with the lobe margin that carry the apothecium developing into a ligulate to triangular spur. Ascospores straight or slightly concave, 10–14 × 3–5 µm. Pycnidia not found.

##### Chemistry.

Divaricatic and usnic acid, unknown fatty acid.

##### Distribution and ecology.

Corticolous on branchlets in forest or more open areas at low elevation in the western Mediterranean region, so far confirmed on DNA-basis from the islands of Corsica (France) and Sardinia (Italy); probably more widespread.

##### Remarks.

The type collection of *Ramalina lusitanica* has many small and brittle fragments, with an upper surface with verruciform ridges, reticulate lower surface and several apothecia. Its author considered it was close to “*Ramalina evernioides*” that represents the taxon now named *R. lacera*; he added that it “cannot be considered a variety of that species on account of absolute absence of sorediate parts and of distinct reticulation”. We were able to produce DNA sequences out of material collected in Italy/Sardinia and France/Corsica and therefore to stabilize this epithet erratically used, because of confusion with *R. canariensis* and *R. lacera*. Typical specimens are easily recognized (when young) by their fan-shaped, rather rigid lobes, some being slightly concave, usually longitudinally striate, without fenestrations, usually with abundant and terminal apothecia and production of divaricatic acid.

*Ramalina lusitanica* is resolved as a distinct species in a clade together with *R. huei* and all accessions of *R. requienii* from Macaronesia, here assigned to the newly described *R. krogiae*. However, *R. huei* (Fig. 4D) develops pendulous thalli, usually exuberant (5–20 cm long) and with convoluted lobes, lateral apothecia and pseudocyphellae; when these are lateral, they induce separation of cortex layers, thus exposing the medulla; such features are not encountered in *R. lusitanica*. *Ramalina huei* thrives in the Canary Islands, the Cabo Verde archipelago and southern Portugal (Krog and Østhagen 1980; Aptroot and Schumm 2008). Interestingly, *R. lusitanica* is not closely related to the mainly epiphytic *R. canariensis* and the saxicolous *R. requienii*, both species producing divaricatic acid and occurring abundantly in the Mediterranean region.

Without identification of its secondary metabolite (divaricatic acid), the general appearance of this species brings it close to forms of *R. fastigiata* (producing evernic acid) or *R. panizzei* and *R. elegans* (both producing acids in the sekikaic group). Further information about these species can be found in Arroyo and Seriñá (1995) and Groner and LaGreca (1997).

We considered *Ramalina latzelii* Zahlbr., a species producing divaricatic acid and abundant apothecia, as a putative synonym. This epithet was reduced into synonymy with *R. canariensis* by Poelt (1969) and examination of the type material (W) confirms that it is, indeed, a fertile rather than sorediate form of that species; divaricatic acid is detected by TLC.

##### Additional specimens examined.

France – Corsica, Terzanili; 41°25.21'N, 09°12.37'E; alt. 60 m; 10.2010; M. Guissard & E. Sérusiaux ; olive orchard (LG DNA 1702); [DNA : MN811471 (LSU), MN811275 (ITS), MN757073 (RPB1), MN757273 (RPB2)] Italy – Sardinia, E of Sanat Teresa, La Licciola; 41°13.33'N, 09°15.32'E; alt. 70 m; 10.2010; M. Guissard & E. Sérusiaux leg; on twigs in disused olive plantation; (LG DNA 1525); DNA : MN811462 (LSU), MN811266 (ITS), MN757064 (RPB1), MN757269 (RPB2)] Morocco – Oued “Rotbar, sur racines accidentellement découvertes de *Chamerops humilis*“, 01.06.1937, leg. J. Gattefosse leg. (BC). – Morocco, “forêt de Boulhaut à Aîn Sferdjla, sur *Rhus pentaphylla*“, 20.02.1939, J. Gattefosse leg. (BC). TLC for both collections from Morocco by Amami N., Arroyo & Seriñá, annotation of May 2002.

Type collection of *Ramalina latzelii* Zahlbr., Oesterr. Botan. Zeitschrift 60: 18 (1910): Croatia – “Dalmatien, Meleda, an *Pinus halep*. auf der Grabova”, ca. 200 m, 18.02.1908, leg. Dr. A. Latzel n° 22” (W! – holotype).

#### Ramalina rosacea

Taxon classificationFungiLecanoralesRamalinaceae

(Massal.) Hepp, Flechten Europ. n° 356 (1857)

46753D3D-D9A6-5995-AFC6-C9913F862C9B

403822

var. Bas.: Ramalina polymorpha var. rosacea Massal., Schedul. Critic., fasc. IX: 157 (1856).
= R. bourgeana auct. europ., non Mont. ex Nyl.

##### Type.

Corsica, Cavallo, Lich. Exs. Ital. 228 (BM! – isotype).

##### Description.

Thallus saxicolous, firmly attached to the substrate (rock), formed of rigid, rather large lobes (2–8 cm large and 1–14 cm long) almost all attached with a single holdfast, lobes surface strongly reticulate-wrinkled. Apothecia usually present, marginal, usually at lobe extremities, with an outer exciple strongly scrobiculate. Ascospores straight or slightly curved, 1-septate, 10–12 × 3–5 μm. Pycnidia not found.

##### Chemistry.

Bourgeanic, norstictic, stictic and cryptostictic acid, PCR-1 and triterpenes.

##### Distribution and ecology.

Very rare, found on rocky sea-shores at two localities in the western parts of the Mediterranean Sea (France/Corsica and Spain; see below).

##### Remarks.

The original material was collected on the island of Cavallo, a small islet south of Corsica (France) and distributed through the Lichenes Italici Exsiccati, n° 288. The original publication (Schedulae criticae in lichenes exsiccatos Italiae IX n° 286–323) can be accessed through the permanent link http://mdz-nbn-resolving.de/urn:nbn:de:bvb:12-bsb10229836-1. We could examine the material preserved in BM: an original handwritten annotation is “Cavallo”. It matches very well the recent collection made on the very same islet by Gonnet et al. (2017) which we could examine (including by TLC and ITS sequence). The secondary metabolites of that material were identified as usnic, bourgeanic and salazinic acids (Gonnet et al. 2017); we cannot confirm these results. Indeed, following the protocols designed by Culberson et al. (1981), we recognize the following as produced by the recent accession from Cavallo and two from Cabo de Gata (Spain): norstictic (+), stictic (++), cryptostictic (+++), and bourgeanic acid and triterpenes. Besides bourgeanic acid and the triterpenes, the main coumpound is the poorly known cryptostictic acid, easily confused in solvent G with salazinic acid (Suppl. material 8). We thus confirm the results of Krog and Østhagen (1980), who stated that this material contains depsidones in the stictic acid group. This confusion was the source of a nomenclatural imbroglio as to the identity of the material recently collected at Cavallo with *R. bourgeana* (Roux et al. 2019).

Quite interestingly, this easily recognized species (at least in the local Mediterranean context) is absent elsewhere in southern Corsica and northern Sardinia where two of us (MG and ES) looked carefully for it in several localities, including on other islets of the Lavezzi archipelago, the archipelago to which the island of Cavallo belongs. See for more at: http://www.afl-lichenologie.fr/Photos_AFL/Photos_AFL_R/Textes_R/Ramalina_bourgeana.htm.

The ITS barcode sequence of this material is strictly identical with that of a second population of that species found at the Cabo de Gata in SE Spain, a coastal locality most famous for its lichen flora (Egea and Llimona 1994) and which also is the type locality of *Ramalina clementeana* (Llimona and Werner 1975). Our results clearly demonstrate that it is a unique taxon, different from accessions from Macaronesia and referred to as *R. bourgeana* Mont. ex Nyl. (1870), following Krog and Østhagen (1980). All other reports of both species (*R. bourgeana* and *R. rosacea*) from the Mediterranean region are not confirmed and refer to other species, mostly *R. tingitana*.

##### Selected specimens examined.

France – Corsica, Cavallo Island; 41°22'N, 009°15'E; alt. 0–10 m; 2014; D. & O. Gonnet s.n. leg; rocky sea-shores (hb, LG DNA 4642); [DNA: MN788731 (ITS)]. Spain – Sierra del Cabo de Gata, path W of Torre de Vela Blanca to lighthouse; 36°43.82'N, 02°10.46'W; alt. 150 m; 2007; P. van den Boom 3835 leg; on exposed outcrops (hb van den Boom, LG DNA 426); [DNA: GU726357 (LSU), GU827316 (ITS), MN757014 (RPB1), MN757229 (RPB2)].

#### Ramalina sarahae

Taxon classificationFungiLecanoralesRamalinaceae

Knudsen et al.

652E0894-4DB4-528D-95D2-038FF24E5542

##### Remarks.

This species belongs to the *Ramalina lacera* group (Sérusiaux et al. 2010). It was recently described from the Channel Islands in California, USA (Knudsen et al. 2018). Its ITS sequence is available (Accession: NR_160636.1) and appears identical with all our accessions of *R. lacera* (sensu Nash et al. 2002) from Mexico/Baja California and Baja California Sur, one accession of *R. lacera* s.l. from the archipelago Cabo Verde in the Atlantic Ocean off the coast of Mauritania and several accessions from the coastal areas of Namibia that would all be referred to as *R. lacera* following Wirth (2010b). The discovery of *R. sarahae*, throughout most of the peninsula of Baja California, in the Namib desert and the Cabo Verde archipelago creates an interesting relationship between these regions and it is worth mentioning that *R. sarahae* cannot be detected in any of the three other archipelagoes forming Macaronesia: Canary Islands, Madeira and Azores.

##### Selected specimens examined.

Cape Verde archipelago – São Vicente, Monte Verde, just below the summit, NW slope; 16°52.2'N, 024°56.0'W; alt. 700 m; 2006; P. van den Boom 36603 & 36603b leg; on twigs (hb, LG DNA 1963 and 1964); [DNA 963: MN788729 (ITS); DNA 964: MN788730 (ITS)]; Namibia – East of the road Swakopmund-Henties Bay; 22°20.38'S, 014°26.44'E; alt. 20 m; 04.2016; E. Sérusiaux s.n leg.; desert dunes, on twigs in “lichen field” (LG DNA 5012); [DNA: MN788735 (ITS)] – Omaruru distr., Laguneberg, N of Mile 72; ca. 21°49.8'S, 014°04.6'E; alt. 70 m; 09.2007; V. Wirth 40683 & 40686 leg; on twigs (KR, LG DNA 563 and 564); [DNA 563: MN788727 (ITS); DNA 564: MN788728 (ITS)]; – N-E of Cape Cross, southern part of the Laguneberg Range; 21°39.31'S, 013°59.55'E; alt. 100–130 m; 04.2016; E. Sérusiaux leg; rocky outcrops in “lichen field”, on twigs (LG DNA 5021); [DNA: MN788735 (ITS)] Mexico – Baja California, laguna and peninsula of San Quintín; 30°29.74'N, 116°00.04'W; alt. 4 m; 02.2016; heavily disturbed chaparral over volcanic rocks; R. Spjut & E. Sérusiaux 17032 leg; on branches of *Aesculus parryi* (hb WBA, LG DNA 4676; [DNA: MN788731 (ITS)] – Baja California, El Rosario, Punta Baja; 29°58.26'N, 115°47.26'W; alt. 70 m, 02.2016; R. Spjut & E. Sérusiaux 17090 leg; on twigs and branches of small shrub (hb WBA, LG DNA 4728); [DNA: MN788732 (ITS)]; – Baja California Sur, SE of Bahía de Asunción, near the coast; 27°09.81'N, 114°14.75'W; alt. 20 m; 02.2016; R. Spjut & E. Sérusiaux 17136 leg; on twigs; (hb WBA, LG DNA 4756); [DNA: MN788733 (ITS)] – Baja California Sur; along the road from Bahía de Tortugas to Vizcaíno; 27°37.82'N, 113°25.19'W; alt. 70 m; 02.2016; R. Spjut 17233c & E. Sérusiaux leg; on twigs (hb WBA, LG DNA 4823); [DNA: MN788734 (ITS)].

### 4 *Vermilacinia*

Saxicolous species of *Vermilacinia* are often described to have cylindrical prismatic branches, cylindrical for their lengthwise three-dimensional shape that in x-section are ± round in outline but with short line segments to form a polygonal shape as opposed to teretiform being uniformly round in x-section. The lichen metabolites zeorin, T3 (terpene, UV+ orange), and [-]-16α-hydroxykaurane are usually present. Exceptions are T3 lacking in species with T1 & T2 (Rf class 2–3, Solvent G) and zeorin sometimes absent in *V. combeoides* and *V. rigida*.

#### Vermilacinia breviloba

Taxon classificationFungiLecanoralesRamalinaceae

Spjut & Sérus. sp. nov.

BF660F21-B81B-5A8A-ADD7-07804AAF74ED

833607

Fig. 13A, B

##### Diagnosis.

Similar to *V. robusta* by the inflated branches and to *V. polymorpha* by the relatively short length of branches; differs by the honeycomb-like cortex or by the contorted lobes.

**Figure 13.**
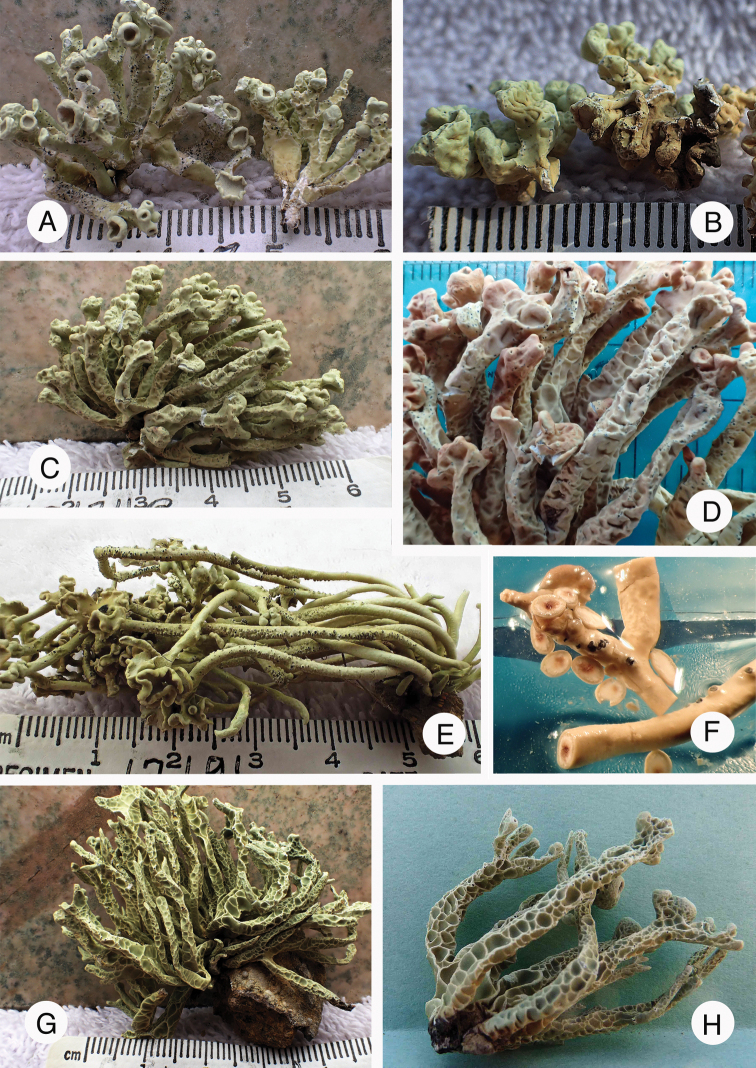
New species of saxicolous *Vermilacinia***A, B***V. breviloba* (holotype) **C, D***V. lacunosa* (holotype) **E, F***V. pustulata* (holotype) **G, H***V. reticulata* (holotype). Scale: 1 mm (**A, C**). Photographs by R. Spjut.

##### Type.

Mexico – Baja California, Pacific Coast ca. 100 km N of Guerrero Negro, just N of Punta San Rosalillita west of road to Punta Negra along track to Puerto San Andrés in a narrow arroyo leading to a tidal inlet (estuary); 28°42.62'N, 114°16.19'W, alt. 50 m, 26.01.2016, R. Spjut & E. Sérusiaux 17117 leg.; on steep north-facing rock ledges bordering south-side of tidal marsh, (LG ! – holotype; BCMEX !, US !, hb. Spjut at World Botanical Associates! – isotypes) [TLC : salazinic acid, triterpene 3, zeorin, [-]-16α-hydroxykaurane ; DNA : MN811491 (LSU), MN811295 (ITS), MN757090 (RPB1), MN757285 (RPB2), MN757407 (GDP), MN757544 (EF-1α)]

##### Description.

Thallus 1–1.5 (-2.5) cm high and 0.5–1 cm broad; basal branches 1–5 or rarely more, short cylindrical, teretiform or prismatic, 1–3 mm diam., loosely united at brownish base, ± erect, inflated, irregularly shriveled and contorted when dry, transversely segmented and ruptured when wet at ± regular intervals, terminally divided into short lobes with or without apothecia; terminal lobes often many and close together or fewer and spreading, 4–6 mm long, 2–4 mm diam. Cortex two-layered, 35–50 μm thick, outer thicker, melanized, externally pale olive green, with irregular reticulate cortical ridges, recessed-concave within ridges (honeycomb-like surface), occasionally plicate on inflated lobes. Medulla subfistulose, hyphae flexuous when wet, intertwining in a net arrangement, ± periclinal, frequently uniting into minute knots; Photobiont in small yellow green to green round colonies ± continuous around the perimeter of the medulla. Apothecia many, aggregate terminally on a primary branch, each subtended by a short stalk-like lobe partly deflated and constricted to junction with branch lobe, bowl-shaped when young, to 4 mm diam., lenticular with age; thalline margin thickened, incurved, entire or crenulate or incised, disc pale orange, concave; asci 8-spored; spores opaque, 1-septate, short ellipsoid, 6–7 × 4–5 μm. Pycnidia black, common on the upper half of branches in shallow concave depressions within cortical ridges, ostiole flush with cortical surface, immersed below; conidia not observed.

##### Chemistry.

Salazinic acid, triterpene 3, zeorin, [-]-16α-hydroxykaurane.

##### Distribution and ecology.

Mexico, Baja California, North Vizcaíno Desert, between Punta Santa Rosalillita and Punta Negra. Only known from that locality. On rock ledges of north-facing cliffs bordering estuary inland from the sea, occurring with species of *Niebla*, *Vermilacinia cedrosensis* and *V. paleoderma*, within a semicircular arc of volcanic coastal hills with steep ravines and narrow ridges trending in various directions, 200–400 m in altitude, extending approx. 20 km along the coast and to 7 km inland at midpoint near the Punta Negra Road (Google Earth 2019). Fog often lingers amongst the higher ridges and peaks during the day (Spjut 1996). A diversity of saxicolous *Vermilacinia* occurs here: *V. breviloba*, *V. cedrosensis*, *V. ligulata*, *V. paleoderma*, *V. pustulata*, *V. reptilioderma* and *V. rigida*. Vegetation on hills near type locality consists of spiny shrubs and succulents of *Pachycormus discolor*, *Stenocereus thurberi*, *Cylindropuntia* spp., *Fouquieria diguetii* and *Agave shawii*. The subshrub *Xylonagra arborea* was observed on rocks amongst the lichens and salt-scrub of *Atriplex julacea* and *Frankenia palmeri* bordered a salt marsh of aquatic species not studied.

##### Etymology.

Epithet *breviloba* refers to the short lobes.

##### Remarks.

*Vermilacinia breviloba* appears related morphologically to *V. polymorpha* and *V. robusta*, neither of which could be included in our phylogeny. *Vermilacinia polymorpha* was described by Bowler et al. (1994) from a specimen collected by Janet Marsh on Santa Catalina Island. *V. robusta*, a widespread species, differs by its much larger, terminally round inflated branches with a relatively smooth cortical surface. *Vermilacinia polymorpha* differs by its deflated-canaliculate branches near base. Both *V. polymorpha* and *V. robusta* occur in the USA/California and Mexico/BC Chaparral, mostly in the Channel Islands; the latter species is also on Isla Guadalupe.

##### Additional specimens examined.

Same locality as the type: R. Spjut & E. Sérusiaux 17121, 17126, 17128b, 17129b.

##### Conservation Status.

Prior to year 2000, Puerto San Andrés was accessible directly from San Andrés Ranch, which appeared occupied at the time and not far from where *V. breviloba* occurs (Spjut pers. obs.). In January 2016, the ranch was not seen while we observed a new earth road that circumvented the estuary by passing north over a saddle and down across a wide arroyo to the north end of Puerto San Andrés, ca. 7 km southeast of Punta Rocosa via a precipitous rocky coastline (Google Earth 2019). A mixed community of local fishermen and nomads appear to reside at Puerto San Andrés. The most disturbance to lichens – evident to Spjut – was on the volcanic hill along the earth road that passes between the estuary and the arroyo north of the pass and also at the north end of Puerto San Andrés. An example is a strongly inflated form of *N. podetiaforma* observed in May 1985 to be common on pebbles on the rain shadow side of the hill (Spjut 1996, coll. Spjut and Marin 9077, distributed to many herbaria). This species was not seen there during our Jan 2016 visit. Two other earth tracks from Punta Negra road towards the coast could not be found in January 2016, one that seemed to have been created sometime between May 1986 and March 1988 that led to Punta Rocosa through Krutsio Ranch and a much older track originating about midway between Puntas Negra and Santa Rosalillita that led into the hills along a narrow arroyo where Spjut, Marin and McCloud in May 1986 found closer foot access to the higher elevation ridges. This latter track, which was not evident from ground level in Jan 2016, is evident from Google Earth (2019), whereas the track to Krutsio Ranch appears to have weathered beyond recognition along with the ranch. Thus, much of this rocky coastal region between San Andrés and Punta Negra is protected by its isolation from being inaccessible by road. Additionally, the type locality is accessible only by foot in a direction opposite to where visitors travel to Puerto San Andrés.

#### Vermilacinia lacunosa

Taxon classificationFungiLecanoralesRamalinaceae

Spjut & Sérus. sp. nov.

0268505B-07C0-5CC7-B231-5D18E3027EAA

833608

Fig. 13C, D

##### Diagnosis.

Similar to *V. reptilioderma* morphologically by the cylindrical-prismatic branches and chemically by the triterpenes T1 and T2, but differing in chemistry by the additional lichen substance, methyl 3,5-dichlorolecanorate (tumidulin).

##### Type.

Mexico – Baja California Sur, Vizcaíno Peninsula, 2.5 km SE of Punta Eugenia, rock outcrops along coastal hills trending west-east separated by wide arroyo, just east of the coastal community of La Lobera; 27°49.701'N, 115°03.454'W; alt. 35–40 m; 29.01.2016, R. Spjut & E. Sérusiaux 17174 leg.; on calcareous rocks of north facing slope; (LG! – holotype; BCMEX!; US!; hb. Spjut at World Botanical Associates! – isotypes) [TLC: Triterpenes 1 & 2, zeorin, [-]-16α-hydroxykaurane, usnic acid, methyl 3,5-dichlorolecanorate (= tumidulin), two unknown triterpenes; DNA: MN811420 (ITS), MN757203 (RPB1), MN757370 (RPB2), MN757488 (GDP)]

##### Description.

Thallus divided into many subcylindrical branches from a basal reddish-brown to blackish holdfast, to 2.5 cm high and broad. Primary branches ascending to erect, ± ellipsoid-arcuate in x- section, simple or once dichotomously divided near mid region, terminating in aggregate of up to 8, commonly 5, apothecia or with single apothecium, or apothecia not fully developed on most branches, occasional branches without apothecia tapering to obtusely rounded apex; surface of branches commonly lacunose, deeply recessed within reticulate or circular cortical ridges when dry. Cortex pale yellow green, 50–125 μm thick, each of two layers equal in thickness, outer melanized, inner pale. Medulla hyphae flexuous when wet, intertwining in a net arrangement, ± periclinal, frequently uniting into short knots; photobiont in small yellow green clusters irregularly discontinuous around perimeter. Apothecia subsessile, differentiated from branch by constriction or very short stalk-like lobe, bowl-shaped, to 2 mm diam. Alternatively, wider with shallower disc in age, thalline margin not differentiated by thicker cortex, incurved, entire or crenulate with age, disc pale yellow green or yellowish with age, concave; asci 8-spored; spores not observed outside asci. Pycnidia black, common on upper branches and on apothecia, mostly along cortical ridges, immersed except for ostiole flush with surface, conidia straight, short, needle-like.

##### Chemistry.

Triterpenes 1 & 2, zeorin, [-]-16α-hydroxykaurane, usnic acid, methyl 3,5-dichlorolecanorate (tumidulin), unknown triterpenes just below and above T1 and T2, respectively (TLC solvent G).

##### Distribution and ecology.

Mexico, Baja California Sur, Vizcaíno Peninsula. Known only from a single collection on calcareous rocks on the north slope facing towards a wide arroyo just inland from the sea on the far western Vizcaíno Peninsula, occurring with *Vermilacinia paleoderma* and vascular plants *Fouquieria diguetii*, *Pachycormus discolor*, *Eriogonum pondii* and *Gossypium* sp. This region lies within the El Vizcaíno Biosphere Reserve, the largest reserve in Mexico. Additional details on the vegetation of the Vizcaíno Peninsula can be found in Peinado et al. (2005).

##### Etymology.

Epithet *lacunosa* refers to the cortical depressions or ‘holes’ in the branch.

##### Remarks.

*Vermilacinia lacunosa* is a distinct saxicolous species for containing the rare lichen metabolite methyl 3,5-dichlorolecanorate (tumidulin), identified by its high Rf on TLC plates in two specimens, previously known only from South American epiphytic species of *Vermilacinia* (Spjut 1996), such as *V. flaccescens* (Nyl.) Spjut and Hale. Sipman (2011) subsequently reported finding what he interpreted to be tumidulin in a new sorediate species he named *Niebla granulans*, here regarded as *Vermilacinia* [*V. granulans* (Sipman) Spjut and Sérusiaux (comb. nov.); bas. *Niebla granulans* Sipman, Bibliotheca Lichenologica 106: 300, 2011] found on twigs at Zapallar in the Valparaíso region of Chile. He distinguished it by apical punctiform soralia in contrast to lateral disciform soralia of *V. cephalota*; he further differentiated it from *V. cephalota* by the intricately branched habit; however, Spjut has collected much branched *V. cephalota* near Bahía de Asunción and distinguished *V. leonis* in the Magadlena Region of Baja California Sur for its larger much branched thalli (Spjut 1996). Additionally, Sipman (2011) reported four undetermined terpenoid compounds, one of which was likely a diagnostic *Vermilacinia* compound, [-]-16α-hydroxykaurane, based on his observation of “blooming of terpenoid crystals in herbarium specimens.” The species was noted to lack chondroid strands, pycnidia and apothecia.

In contrast, Sipman (2011) described *Niebla nashii*, another Chilean sorediate species from Coquimbo that he compared to *Ramalina lacera* (With.) J.R. Laundon. He distinguished it by possessing isolated medullary chrondroid strands, as well as tumidulin and bourgeanic acid, while he also noted that key [*Vermilacinia*] terpenoids, pycnidia and apothecia were absent. Judging from Sipman’s image of the type specimen, it lacks the characteristic cortical ridging of *Niebla*. Further, Sérusiaux et al. (2010) found bourgeanic acid associated with depsides in Mediterranean species of *Ramalina* – that had been treated in *Niebla* by Rundel and Bowler (1978) and Bowler and Marsh (2004) – to be nested within the genus *Ramalina*. Other collections identified *Niebla* spp. from Patagonia in Chile (National Park of Torres Del Paine) – that were reported to contain tumidulin – “exhibited significant inhibitory activity on spheroid formation in CRC cells and decreased the expression of CSC markers in CRC cells” (Yang et al. 2018). DNA extracts are needed to assess the phylogenetic relationships of the Chile specimens reported to contain tumidulin.

##### Conservation Status.

The type locality of *Vermilacinia lacunosa*, 2.5 km southeast of the fishing community of Punta Eugenia, is vulnerable to off-road travel. Spjut observed the Punta Eugenia community to have expanded considerably since his first visit there in 1986. Although the type locality lies within the El Vizcaíno Biosphere Reserve, this reserve is referred to as a wildlife refuge. However, to the northeast is the protected area of flora and fauna at Valle de los Cirios in the southern portion of Baja California. “The El Vizcaíno overlaps with smaller protected areas on land and in the waters, including the protected grey whale (*Eschrichtius robustus*) sanctuaries at Ojo de Liebre (Scammon’s Lagoon), Guerrero Negro, and San Ignacio. Protection is provided by international organizations including UNESCO, Ramsar and Western Hemisphere Shorebird Reserve Network (WHSRN). The area is not only important to plant and animal life, but more than 300 ancient rock painting sites have been discovered throughout the reserve” (CONANP-20: http://www.parkswatch.org/parkprofile.php?l=eng&country=mex&park=vibr&page=inf&p=mex; accessed 09.06.2019).

#### Vermilacinia pustulata

Taxon classificationFungiLecanoralesRamalinaceae

Spjut & Sérus. sp. nov.

FD38F7B0-A4AB-5B86-B48F-AACDD5A0C37E

833609

Fig. 13E, F

##### Diagnosis.

Similar to *V. cedrosensis* in the flexuous long cylindrical branches with a pale yellow-green cortex, but differs by the pustular cortical protrusions, in contrast to pitted and transversely reticulated cortex of *V. cedrosensis*.

##### Type.

Mexico – Baja California Sur, Vizcaíno Peninsula, ca. 11 km NW of Bahía Tortugas, 2.6 km NE of Rompiente along the west side of peninsular Coast, west to southwest along track off the Bahía Tortugas-Punta Eugenia Road; 27°44.969'N, 114°56.690'W; alt. 140–160 m; 30.01.2016, R. Spjut & E. Sérusiaux 17191 leg.; on white calcareous rock outcrops along coastal hills trending northwest, (LG! – holotype; BCMEX!; US!; hb. Spjut at World Botanical Associates! – isotypes); [TLC : Salazinic acid, triterpene 3, zeorin, [-]-16α-hydroxykaurane, unknown triterpene UV+ bright blue Rf just below T3, traces of several other unknown triterpenes; DNA : MN811556 (LSU), MN811360 (ITS), MN757151 (RPB1), MN757492 (GDP), MN757618 (EF-1α)]

##### Description.

Thallus divided into many long, uniformly narrow cylindrical-teretiform, flexuous branches from a pale brown to blackened base, to 7.0 cm long and 1 cm diam. at base. Primary branches fastigiate, ascending near base, flexuous above, simple or dichotomously divided, 1–2 mm diam., terminating in aggregate of few to several apothecia, occasional branches obtuse to a blunt apex; surface of branches densely pustular and blistered, conspicuously in a line along one side. Cortex pale yellow green or whitish-green, 40–60 μm thick. Medulla with strong orange pigmentation in central region within the lower half of branches, becoming pale yellow and then clear in upper half or medulla pale yellow or without noticeable pigmentation; photobiont in small yellowish-green colonies irregularly dispersed around the perimeter of medulla. Apothecia mostly terminal, occasionally subterminal well below apex, developing from a short terminal expansion and flattened lobe, bowl-shaped when young, to 5 mm diam.; thalline margin lip-like, incurved to disc, becoming deeply lobulate; disc pale yellow green to pale grey or white or yellowish with age, plane to slightly concave; asci 8-spored; mature spores not seen outside asci. Pycnidia black, conspicuous on conical protrusions, immersed except for ostiole flush with surface.

##### Chemistry.

Salazinic acid, triterpene 3, zeorin, [-]-16α-hydroxykaurane, unknown triterpene UV+ bright blue Rf just below T3, traces of several other unknown triterpenes, one above salazinic acid and another above T3 (TLC in Solvent G).

##### Etymology.

Epithet *pustulata* refers to the pustular outgrowths on the cortex.

##### Distribution and ecology.

Mexico, Baja California Sur, Vizcaíno Peninsula and Baja California, Punta Morro Santo Domingo and Puerto San Andrés.

##### Remarks.

*Vermilacinia pustulata* is closely related to *V. cedrosensis* from which it can be distinguished by its surface of branches densely pustular and blistered, conspicuously in a line along one side. This species is resolved as sister to *V. reptilioderma*, a species easily distinguished by its cylindrical-prismatic branches and production of the triterpenes T1 and T2. All species delimitation methods recognized the two as different, except for STACEY which merged them. This incongruence may require further investigation.

##### Conservation Status.

The species appears threatened at type locality by trash discarded in the open desert, observed to be increasing in density as one approaches within several km from the west side of Bahía Tortugas. Fortunately, the species occurs elsewhere.

##### Additional specimens examined.

Mexico – Baja California, west of Villa Jesus María along shoreline at Punta Morro Santo Domingo; elev. 10 m; 12.2016; S. Leavitt et al. 16–938 leg. (BRY!); ibid.; vicinity of Puerto San Andrés; 19.05.1986; R. Spjut 9893A1 leg. (hb. Spjut at World Botanical Associates).

#### Vermilacinia reticulata

Taxon classificationFungiLecanoralesRamalinaceae

Spjut & Sérus. sp. nov.

1DEA0EA5-1CD8-55A2-8453-3EE7A5DFF7DB

833610

Fig. 13G, H

##### Diagnosis.

Recognized by its thallus divided into subcyclindrical-prismatic branches, appearing quadrangular in x-section, with sharply raised ± wavy ridges in a reticulate pattern, the primary branches shortly 5-lobed near apex.

##### Type.

Mexico – Baja California Sur, Vizcaíno Peninsula, 2.5 km SE of Punta Eugenia, rock outcrops along coastal hills trending west-east separated by wide arroyo, just east of the coastal community of La Lobera; 27°49.701'N, 115°03.454'W; alt. 35–40 m; 29.01.2016, R. Spjut & E. Sérusiaux 17153 leg.; on calcareous rocks of north facing slope; (LG! – holotype; BCMEX!; US!; hb Spjut at World Botanical Associates! – isotypes)[TLC : Salazinic acid, triterpene T3, zeorin, [-]-16α-hydroxykaurane, unknown triterpenes ; DNA : MN811551 (LSU), MN811355 (ITS), MN757146 (RPB1), MN757332 (RPB2), MN757486 (GDP), MN757613 (EF-1α)]

##### Description.

Thallus divided into several or many subcylindrical-prismatic branches from a common basal attachment, up to 6 cm high and 5.5 cm broad. Primary branches ascending, once dichotomously divided near mid region, terminally shortly 5-lobed, with apothecia or, if without apothecia, obtusely rounded to bluntly pointed apex, compressed, 4-lobed or angled in x-section, occasional branches dilated and flattened to apex, sharply recessed along cortical ridges mostly in a reticulate pattern. Cortex pale yellow green or bluish green, smooth and deeply recessed within the ± reticulate or circular ridges along the face of the branch, 2-layered or a dense algal layer in outer medulla closely adhering as a third supportive layer, 75–150 μm thick. Medulla with a central dense grey area and outer white area. Apothecia on a short compressed lobe slightly constricted at junction with primary branch, bowl-shaped, to 2 mm diam. or up to 5 mm diam. with less concave disc or appearing to abort development, the branch terminally inflated and lobulate; thalline margin incurved, entire or crenulate with age, disc pale yellow green or yellowish with age or cream, concave; spores opaque, 1-septate, fusiform-curved, 8–10 μm. Pycnidia black, common to abundant on upper half of branches, immersed except ostiole flush with surface; conidia needle-like.

##### Chemistry.

Salazinic acid, triterpene T3, zeorin, [-]-16α-hydroxykaurane, unknown triterpenes UV + light blue without border, UV+ dark blue with border at Rf just above salazinic acid (TLC in solvent G).

##### Distribution and ecology.

Mexico, Baja California Sur, Vizcaíno Peninsula, known from a single location on conglomerate outcrops along north to northeast facing slopes near Punta Eugenia, occurring with *Vermilacinia paleoderma*.

##### Remarks.

*Vermilacinia reticulata* is distinguished from *V. paleoderma* by the sharply delineated cortical ridges and deeply recessed surface within, often appearing in a reticulate pattern. TLC revealed an unknown UV+ dark blue with a well-defined border under UV+ just above salazinic acid. Thalli with concentrated development of pycnidia on rugose cortical ridges resemble *Niebla rugosa*, which differs by the stepladder arrangement of the cortical ridges, in addition to the chemical and medulla character features that define the genus and species. *Vermilacinia reticulata* is sister to a clade comprising *V. paleoderma* and the newly described *V. breviloba*.

##### Etymology.

Epithet *reticulata* refers to the cortical ridges exhibiting a nice and obvious reticulate pattern.

##### Conservation Status.

The species may be threatened by off-road travel as discussed under *V. lacunosa*.

##### Additional specimens.

Same at the all type locality: R. Spjut & E. Sérusiaux 17175a [DNA: MN811502 (LSU), MN811306 (ITS), MN757420 (GDP), MN757557 (EF-1α)], 17179c [DNA: MN811504 (LSU), MN811308 (ITS), MN757101 (RPB1), MN757423 (GDP), MN757559 (EF-1α)], 17173D [DNA: MN811552 (LSU), MN811356 (ITS), MN757147 (RPB1), MN757487 (GDP, MN757614 (EF-1α)] (all: LG, hb Spjut at World Botanical Institutes).

### Key to saxicolous and terricolous species of *Vermilacinia*

Key based on Spjut (1996), specimens collected by Spjut and Sérusiaux (2016), specimens from BRY loaned to WBA: 400 specimens collected by Steve Leavitt et al. in Baja California, Dec 2016 and another ca. 20 specimens from Chile, March 2017. New species are marked with *.

**Table:** 

1	Thallus branches densely compacted into hemispherical moss-like cushions (Cladonia-Cladina habit), 0.5–2.5(–5.0) cm high, often broader or in irregularly shaped clumps with basal prostrate branches from which ascending to erect secondary branches arise; terminal branches long to short bifurcate or abruptly pointed to obtuse apex; apothecia absent or undeveloped on most branches or, if developed, strictly subterminal with extended spur-like branch	**2**
–	Thallus branches not tightly compacted into moss-like cushions, generally > 3.0 cm high, taller than wide; branches ribbon-like (flattened and contorted), blade-like (compressed-straight) or long tubular or cylindrical-prismatic, generally erect-fastigiate or spreading outward from one another, often branching in mid region, as well as near apex, not regularly bifurcate near apex; apothecia terminal or nearly so, often aggregate, rarely absent	**6**
2	Thallus terricolous; basal branches prostrate to ascending near tip, with occasional upright secondary branches or thallus of numerous capillary matted branches (< 1.0 mm diam.; Ramalina ceruchis var. tumidula (Tayl.) Howe, probably a distinct species); terminal branches long bifurcate and attenuate to apex; apothecia absent; Peru, Chile	***V. ceruchis***
–	Thallus terricolous or saxicolous, branches ascending to erect; mainly N America	**3**
3	Branches (0.5-) 1–2.0 (-5.0) mm diam., terminally swollen, then abruptly tapered to a pointed obtuse apex, simple to occasionally bifurcate or rarely trifurcate near apex, never isidiate; thallus similar to *V. combeoides* that differs by the development of terminal apothecia or by truncated apices on branches without apothecia.	***V. pumila***
–	Branches mostly ≤ 1.0. mm diam.; with short acicular terminal branchlets or isidiate	**4**
4	Branches not united by holdfast; terminal branches equally short bifurcate to apex; isidioid branchlets and/or isidia lacking	***V. ceruchoides***
–	Branches arising from a common base or holdfast, unequally very short bifurcate or trifurcate near apex, occasional to frequent isidioid branchlets below apex; isidia often present; rarely sorediate	**5**
5	Pycnidia absent or only at apex of terminal or isidioid branchlets; isidia often present; apothecia absent; California and Baja California Chaparral, Chile Atacama Desert	***V. acicularis***
–	Pycnidia common to near base of branches; apothecia present, with short spur branchlets; rare, California Chaparral – San Luis Obispo County, Morro Bay	***V. tuberculata***
6	Apothecia subterminal well below apex or lateral – facing perpendicular away from primary branch; Peru, Chile (*V. ceruchis* variants, Spjut 1996)	**V. cf. ceruchis**
–	Apothecia terminal or subterminal; N America	**7**
7	Pair of triterpenes present in R_f_ class 2–3 (TLC, Solvent G)	**8**
–	Triterpenes absent in R_f_ class 2–3	**12**
8	Methyl 3,5-dichlorolecanorate (tumidulin) present	***V. lacunosa*** *
–	Tumidulin absent	**9**
9	Branches irregularly shaped, neither blade-like nor cylindrical, expanded near apex; Islas San Roque and Cedros, western Vizcaíno Peninsula	***V. rosei***
–	Branches ± regular in shape, compressed or teretiform, sublinear to ± oblong	**10**
10	Branches cylindrical-prismatic	***V. reptilioderma***
–	Branches compressed, ribbon-like (flattened, contorted) or bladelike	**11**
11	Branches twisted (ribbon-like)	***V. ligulata***
–	Branches straight to recurved (blade-like)	***V. johncassadyi***
12	Branches more blade-like (compressed) than cylindrical	**13**
–	Branches generally cylindrical-round or cylindrical-prismatic or tubular-inflated	**14**
13	Basal branches 0.5–2 cm long, closely fastigiate; rare, N Vizcaíno Desert – coastal ridge south of Punta Negra N of Punta Santa Rosalillita	***V. rigida***
–	Basal branches mostly 3–6 cm long; spreading apart from base towards apex; California and Baja California Chaparral	***V. laevigata***
14	Primary branches tubular inflated, loosely united at base	**15**
–	Primary branches not tubular inflated, generally cylindrical, recessed within reticulate or round cortical ridges, closely united at base	**18**
15	Primary branches irregularly shaped, expanded near apex; S Vizcaíno Desert – Isla San Roque, Chile – Atacama Desert [BRY specimen]	***V. varicosa***
–	Primary branches ± regular in shape, cylindrical, teretiform or prismatic	**16**
16	Basal branches cylindrical prismatic, as least in part, near apex distinctly lobed or with aggregate apothecia; N Vizcaíno Desert – San Andrés Cañon	V. breviloba*
–	Basal branches tubular inflated, simple or irregularly shortly lobed near apex	**17**
17	Branches broadly rounded to apex; California and Baja California Chaparral, mostly islands	***V. robusta***
–	Branches abruptly tapered to pointed apex; Chile – Atacama Desert	**V. aff. robusta**
18	Basal branches mostly simple, closely fastigiate; apothecia terminal and solitary; zeorin often absent; California and Baja California Chaparral, mainland and islands	***V. combeoides***
–	Basal branches spreading outwards above base; apothecia often terminally aggregate, sessile or on short branches or solitary and subterminal; zeorin present	**19**
19	Branches with bladderlike swellings; pycnidia prominent on elevated rugose cortical ridges; rare, N Vizcaíno Desert – between Punta Canoas and Puerto Catarina	***V. vesiculosa***
–	Bladder-like swellings absent; pycnidia on various cortical angular ridges or at base of cortical depressions or at apex of pustular protrusions	**20**
20	Branches cylindrical-teretiform, often flexuous; cortex with shallow depressions or pits and often with transverse fissural cracks	**21**
–	Branches cylindrical-prismatic; cortex not cracked transversely	**23**
21	Cortex dark green, persistent, blackened irregularly from base to apex, notably where in contact with substrate; California and Baja California Chaparral	***V. procera***
–	Cortex relatively thin, yellowish-green, often eroding towards apex, the branches appearing white due to exposed medulla; Vizcaíno Deserts	**22**
22	Pycnidia mostly in shallow cortical depressions or pits; cortex mostly pitted; apothecia rim entire to slightly lobed	***V. cedrosensis***
–	Pycnidia mostly on conical tubercles; cortex also pustular; apothecia lobulate	***V. pustulata*** *
23	Branch surface honeycomb-like, with deep, ± angular depressions	***V. reticulata*** *
–	Branch surface with various shallow depressions, crater-like or with pastry-like creases or relatively smooth and uneven	**24**
24	Thallus much branched; pycnidia only at apex of branches; apothecia absent; terminal branches shortly bifurcate to sharp pointed apex; branch surface regularly recessed at ‘branch nodes’, sharply angled along ridges; rare, N Vizcaíno Desert – Punta Morro Santo Domingo, Atacama Desert	**V. aff. paleoderma**
–	Primary branches sparingly divided; pycnidia below apex; apothecia on some branches; surface of branches variable	**25**
25	Branches ± oblong (< 10× longer than wide), deflated in part, canaliculate, especially near base, rounded along margins, usually with smooth concave depressions; California and Baja California Chaparral	***V. polymorpha***
–	Branches ± linear to broad linear (> 10× longer than wide), cylindrical prismatic; cortex irregularly creased, reticulately ridged, angularly recessed, plicate or the surface smooth and uneven; Vizcaíno Deserts	***V. paleoderma***

## Data accessibility

Alignments and trees have been deposited in TreeBASE (Accession No: S25433).

## Supplementary Material

XML Treatment for Namibialina

XML Treatment for Ramalina crispans

XML Treatment for Ramalina krogiae

XML Treatment for Ramalina lusitanica

XML Treatment for Ramalina rosacea

XML Treatment for Ramalina sarahae

XML Treatment for Vermilacinia breviloba

XML Treatment for Vermilacinia lacunosa

XML Treatment for Vermilacinia pustulata

XML Treatment for Vermilacinia reticulata

## Supplementary materials

Supplementary material 1 Three Major Ecogeographic Areas of Evolution in Fruticose Ramalinaceae Data type occurrence Explanation note Three geographical areas play a special role in the evolutionary history and present range of the fruticose genera of the Ramalinaceae: (1) the coasts of California/USA and Baja California/Mexico; (2) the Atacama and Sechura deserts along the western coasts of South America and (3) the coasts of Namibia and South-West of South Africa. These areas are briefly presented in this section, focusing on their biodiversity, especially for lichenized fungi, and their recent climatic history. Chapter (1) further includes updates on the ecogeographical data and evolutionary interpretation for the genera Niebla and Vermilacinia in Baja California. https://binary.pensoft.net/file/451998This dataset is made available under the Open Database License (http://opendatacommons.org/licenses/odbl/1.0/). The Open Database License (ODbL) is a license agreement intended to allow users to freely share, modify, and use this Dataset while maintaining this same freedom for others, provided that the original source and author(s) are credited.Richard Spjut, Antoine Simon, Martin Guissard, Nicolas Magain, Emmanuël Sérusiaux

Supplementary material 2 Table S2. PCR conditions and primers for each locus Data type molecular Explanation note PCR conditions and primers for each locus: ITS, LSU, RPB1, RPB2, GDP and EF-1α. https://binary.pensoft.net/file/451999This dataset is made available under the Open Database License (http://opendatacommons.org/licenses/odbl/1.0/). The Open Database License (ODbL) is a license agreement intended to allow users to freely share, modify, and use this Dataset while maintaining this same freedom for others, provided that the original source and author(s) are credited.Richard Spjut, Antoine Simon, Martin Guissard, Nicolas Magain, Emmanuël Sérusiaux

Supplementary material 3 Table S3. Table of accessions of the lichen collections studied Data type species data Explanation note Table of accessions of the lichen collections studied, with the following data: name; locality and year of collection; collector(s) and herbarium references; reference accession number in DNA data bank at Uliège (LG herbarium); secondary chemical compounds (as detected by TLC) and GenBank accessions numbers of each locus examined (ITS, LSU, *RPB1*, *RPB2*, *GDP* and *EF-1*α). The first column points to the accessions that were included in the time-calibrated phylogeny for the four genera of fruticose Ramalinaceae studied (*Namibialina*, *Niebla*, *Ramalina* and *Vermilacinia*) and several accessions of their crustose sister genus *Cliostomum*, including the type species *C. corrugatum*. The last five columns (M to Q) refer to the results of species delimitations methods run on *Niebla* and *Vermilacinia* (ABGD; PTP; BPP and STACEY analyses). https://binary.pensoft.net/file/452000This dataset is made available under the Open Database License (http://opendatacommons.org/licenses/odbl/1.0/). The Open Database License (ODbL) is a license agreement intended to allow users to freely share, modify, and use this Dataset while maintaining this same freedom for others, provided that the original source and author(s) are credited.Richard Spjut, Antoine Simon, Martin Guissard, Nicolas Magain, Emmanuël Sérusiaux

Supplementary material 4 Table S4. Time calibration Data type species data Explanation note Time calibration based on a fossil of *Phyllopsora* and constraint on the Ramalinaceae node conducted on the evolutionary tree. https://binary.pensoft.net/file/452001This dataset is made available under the Open Database License (http://opendatacommons.org/licenses/odbl/1.0/). The Open Database License (ODbL) is a license agreement intended to allow users to freely share, modify, and use this Dataset while maintaining this same freedom for others, provided that the original source and author(s) are credited.Richard Spjut, Antoine Simon, Martin Guissard, Nicolas Magain, Emmanuël Sérusiaux

Supplementary material 5 Table S5. Comparison of the identification of the *Niebla* collections Data type species data Explanation note Comparison of the identification of the *Niebla* collections following Spjut (1996) and the number of accessions in the 6-loci analysis and the species as delimited by the BPP and STACEY analysis. https://binary.pensoft.net/file/452002This dataset is made available under the Open Database License (http://opendatacommons.org/licenses/odbl/1.0/). The Open Database License (ODbL) is a license agreement intended to allow users to freely share, modify, and use this Dataset while maintaining this same freedom for others, provided that the original source and author(s) are credited.Richard Spjut, Antoine Simon, Martin Guissard, Nicolas Magain, Emmanuël Sérusiaux

Supplementary material 6 Table S6. Data for the *Niebla* collections studied Data type species data Explanation note Data for the Niebla collections studied: number of localities for each species recognized by BPP and number of species for each locality sampled. https://binary.pensoft.net/file/452003This dataset is made available under the Open Database License (http://opendatacommons.org/licenses/odbl/1.0/). The Open Database License (ODbL) is a license agreement intended to allow users to freely share, modify, and use this Dataset while maintaining this same freedom for others, provided that the original source and author(s) are credited.Richard Spjut, Antoine Simon, Martin Guissard, Nicolas Magain, Emmanuël Sérusiaux

Supplementary material 7 Table S7. Data for the *Niebla* collections studied Data type species data Explanation note Data for the Niebla collections studied: number of localities for each species recognized by STACEY and number of species for each locality sampled. https://binary.pensoft.net/file/452004This dataset is made available under the Open Database License (http://opendatacommons.org/licenses/odbl/1.0/). The Open Database License (ODbL) is a license agreement intended to allow users to freely share, modify, and use this Dataset while maintaining this same freedom for others, provided that the original source and author(s) are credited.Richard Spjut, Antoine Simon, Martin Guissard, Nicolas Magain, Emmanuël Sérusiaux

Supplementary material 8 Identification of the chemistry of *Ramalina rosacea* Data type species data Explanation note Identification of the chemistry of Ramalina rosacea, a coastal saxicolous species of the R. bourgeana-group, endemic to the Western Mediterranean sea-shores (2 localities known). https://binary.pensoft.net/file/452005This dataset is made available under the Open Database License (http://opendatacommons.org/licenses/odbl/1.0/). The Open Database License (ODbL) is a license agreement intended to allow users to freely share, modify, and use this Dataset while maintaining this same freedom for others, provided that the original source and author(s) are credited.Richard Spjut, Antoine Simon, Martin Guissard, Nicolas Magain, Emmanuël Sérusiaux
