# Corrigendum: Spjut R, Simon A, Guissard M, Magain N, Sérusiaux E (2020) The fruticose genera in the Ramalinaceae (Ascomycota, Lecanoromycetes): their diversity and evolutionary history. MycoKeys 73: 1–68. https://doi.org/10.3897/mycokeys.73.47287

**DOI:** 10.3897/mycokeys.74.59175

**Published:** 2020-10-30

**Authors:** Richard Spjut, Antoine Simon, Martin Guissard, Nicolas Magain, Emmanuël Sérusiaux

**Affiliations:** 1 World Botanical Associates, PO Box 81145, Bakersfield, California 93380, USA World Botanical Associates Barkersfield United States of America; 2 Evolution and Conservation Biology Unit, Sart Tilman B22, Quartier Vallée 1, chemin de la vallée 4, B-4000, Liège, Belgium Evolution and Conserva­tion Biology Unit Liège Belgium

## Abstract

None

Recently, in a revision of the phylogeny and generic classification of fruticose Ramalinaceae (Spjut et al. 2020), two new combinations and a lectotypification were invalidly published by omission of the identifier. This note validates the names and the lectotypification.

***Namibialina melanothrix* (Laurer) Spjut & Sérus., comb. nov.**

MycoBank No: 837518

= *Ramalina melanothrix* Laurer, Syn. Meth. Lich. 1(2): 290, tab. VIII, fig. 26, 1860, MB 403773

= *Trichoramalina melanothrix* (Laurer) Rundel & Bowler, Bryologist 77: 194, 1974, MB 343799

= *Niebla melanothrix* (Laurer) Kistenich, Timdal, Bendiksby, S. Ekman, Taxon 67: 893, 2018, MB 824407

***Vermilacinia granulans* (Sipman) Spjut & Sérus., comb. nov.**

MycoBank No: 837536

= *Niebla granulans* Sipman, Bibliotheca Lichenologica 106: 300, 2011, MB 569717

***Vermilacinia procera* (Rundel & Bowler) Spjut, Sida, Bot. Misc. 14: 168, 199.**

MycoBank No: 416069

= *Niebla procera* Rundel & Bowler, Phytologia 77: 34, 1994, MB 363506.

**Type.** USA, California, San Luis Obispo Co., found on rock outcrops in high marsh at Morro Bay State Park, Riefner 87–100, left hand specimen (ASU – lectotype, herewith selected).
