# Supplementary material for: The fruticose genera in the Ramalinaceae (Ascomycota, Lecanoromycetes): their diversity and evolutionary history
Source: MycoKeys. 2020 Sep 11;73:1–68. doi: 10.3897/mycokeys.73.47287 (PMC7501315; doi:10.3897/mycokeys.73.47287)
Supplement: Supplementary material 1 — Three Major Ecogeographic Areas of Evolution in Fruticose Ramalinaceae [file mycokeys-73-001-s001.pdf]

Supplementary Data S1

Three Major Ecogeographic Areas of Evolution in Fruticose Ramalinaceae

Three geographical areas appear significant to the evolutionary history of lichens in arid regions. We focus on three fruticose genera of the Ramalinaceae: *Nambialina* (gen. nov.), *Niebla*, and *Vermilacinia*. They have adapted to obtaining moisture from fog in the following coastal regions of our study:

(I) California-Baja California coastal chaparral and Vizcaíno deserts
(II) South America Atacama and Sechura deserts
(III) South Africa coastal fynbos and Namib Desert

The botanical significance of each of these will be briefly discussed. Chapter (I) further includes updates on the ecogeographical data and evolutionary interpretation for the genera *Niebla* and *Vermilacinia* in Baja California.

(I) California and Baja California Coastal Chaparral, Chaparral-Desert Transition and Vizcaíno Deserts

1. California and Baja California Coastal Chaparral

The lichen flora of the Baja California peninsula (Mexico, Baja California and Baja California Sur) is pretty well known as a result of the many splendid contributions by lichenologists to the « Lichen Flora of the Greater Sonoran Desert Region » (Nash et al. 2002, 2004, 2007). The « Greater » portion extends the lichen flora to chaparral, oak woodlands, and conifer forests in southern California, northern Baja California and southern Nevada and all of Arizona; to the deserts of Mojave, Arizona, Vizcaíno, and Magdalena; and to the subtropical vegetation in Baja California Sur and Sonora, Mexico that include lowland mixed deciduous-succulent bushlands, open deciduous montane woodlands/forests, and the evergreen pine-oak woodlands/forests.

California, a botanically rich and diverse region with many endemic species (Raven and Axelrod 1978 ; Calsbeek et al. 2003 ; Lancaster and Kay 2013 ; Baldwin et al. 2017), has undergone major changes in geology, climate and flora since the Cretaceous (Millar 2012). The Farallon Plate subducting under the North America Plate, which began long before the Tertiary, impacted the uplift of the Cascades, Sierra Nevada, Peninsular Ranges and other ranges (Millar 2012 ; Barak et al. 2015). Contact between the Pacific Plate–East Pacific Rise and the North American Plate— from early Oligocene to Middle Miocene (c. 30–15 Myrs)—led to the development of the San Andreas Fault System in which subduction changed to lateral shear—moving the Pacific Plate slowly northwest (Atwater 1970 ; Millar 2012 ; Blakey and Ranney 2018), except for the Transverse Ranges that broke off North America c. 20–16 Myrs and rotated 80–110° clockwise (Kamerling and Luyendyk 1985). Islands along the Pacific Coast, besides those already known, likely existed during that time (Axelrod 1967 ; Millar 1996 ; Blakey and Ranney 2018 ; https://deeptimemaps.com/southwest-north-america-

thumbnails/); some were oceanic islands transported by the Farralon Plate or its fragments (Brusca 2019), while others were seamounts (Paduan et al. 2009; Davis et al. 2010). In northwestern Mexico, oblique rifting along the East Pacific Rise from 12.3–6 Myrs and subsequent spreading to c. 1 Myrs gave rise to the Baja California microplate that coupled to the Pacific Plate (Atwater 1989 ; Paulssen and Vos 2017); the Baja California microplate, which the northern tip currently reaches San Gorgonio Pass, near Palm Springs, California (Brusca 2019), has since moved northwest c. 345 km from its original southernmost position near Puerto Vallarta ("continental" Mexico, Jalisco state) (Busby 2004 ; Fletcher et al. 2007).

During the early to mid Eocene (c. 56–48 Myrs), a warm humid tropical climate prevailed in the Pacific NW (Millar 2012). Onset of glaciation in both the Antarctic and Arctic, starting mid Eocene c. 49–45 Myrs (Moran et al. 2006, Polyak et. al. 2010, Vahlenkampa et al. 2018), was followed by rapid decline of global deep-sea temperatures near the end of the Eocene, c. 34 Myrs (Zachos et al. 2008). In early Miocene, global cooling strengthened the westerlies (Pierrehumbert 2002) that increased upwelling of cool Pacific Ocean water off the Californian coast along with the development of winter precipitation and summer drought by mid-Miocene, c. 15 Myrs (White et al. 1992; Jacobs et al. 2004), a Mediterranean type of climate (Rundel et al. 2016) that intensified in late Miocene, c. 10 Myrs (Minnich 2007). Many of our present-day angiosperm genera in the California chaparral and Sonoran Desert had evolved (Axelrod 1979; Millar and Woolfenden 2016). Notable among these is *Lyonothamnus*, a genus of three extinct and one extant species in the Rosaceae, the genus with distinct foliage, easily identified among Tertiary plant fossil assemblages from California, Nevada and Oregon, now surviving only in the Channel Islands (Wolfe 1964; Erwin and Shorn 2000). During this time Baja California was still part of the mainland of Mexico. The availability of island coastal habitat, the change in latitude of the Pacific Plate, uplift of Coast Ranges during the Pliocene (Miller and Graham 2018), and the oscillating glacial and interglacial climates during the Pleistocene accounts for speciation and migration of *Niebla* and *Vermilacinia* along the Pacific Coast of western North America.

In California, the coastal chaparral is divided into Northwest, Central West, and Southwest Geographical Subdivisions (Baldwin et al. 2017). The central and southern divisions have coastal sage (*Artemisia californica*) scrub that reaches its northernmost distribution at Point Reyes (Mooney 1977; Calflora 2019) and southernmost distribution near San Vicente in Baja California where it merges with the chaparral-desert ecotone (Spjut 1996 ; Rebman and Roberts 2012 ; Delgadillo 2013). It continues south as an inland region terminating at the Sierra San Miguel (Shreve 1936; Rebman and Roberts 2012).

The coastal chaparral is also part of the California Floristic Province (CA-FP) that includes conifer forests. The CA-FP is ecogeographically separated from deserts (Great Basin, Mojave, Sonoran) by the rain-shadow of north-south trending mountain ranges (Cascade Ranges, Sierra Nevada, Peninsular Ranges), but along the coast from California to Baja California, the change from chaparral to desert vegetation is gradual as precipitation gradually decreases southward along the coast. A chaparral-desert ecotone (transition) is recognized between San Vicente and Campo Nuevo just south of Punta San Fernando, by open low stature of shrub vegetation in contrast to dense ground cover of larger shrubs and trees in the coastal chaparral region (Spjut 1996; Vanderplank 2011). This transition is also included in the CA-FP (Burge et al. 2016). Further south, c. 12 km southeast of El Rosario and along the Pacific Coast

to near Punta San Fernando, tall columnar cacti and *Fouquieria* species appear within the Northern Vizcaíno Desert (Shreve 1936; Spjut 1996; Vanderplank 2011).

The Southwest Region of California Coastal Chaparral was regarded by Spjut (1996) as a center of morphological diversity for *Vermilacinia*, especially the Channel Islands where he also recognized two endemic species of *Niebla* on San Nicolas Island.

In Baja California, *Niebla* and *Vermilacinia*, and other lichens are limited to exposed large rock outcrops, often of localized occurrence within the coastal fog belt. Examples of localities where *Niebla* species have been previously collected in the Baja California chaparral region are Rosarito and La Misión just south of Tijuana, Punta Banda, and Punta Santo Tomás (Spjut 1996).

An alternative ecogeographical distinction would be to recognize the Baja California Floristic Province (BC-FP) based in part on phytogeographical data in Ratay et al. (2014), and define it by geological boundaries, the San Andreas Fault at its eastern boundary, the Transverse Ranges at its northern boundary, and the Channel Islands to Isla Guadalupe as a western boundary. The BC-FP include monotypic and oligotypic chaparral and succulent genera: *Adolphia* (Rhamnaceae, *A. californica*), *Bergerocactus* (Cactaceae), *Cneoridium* (Cneoraceae), *Comarostaphylis* (Ericaceae, *C. diversifolia*), *Ornithostaphylos* (Ericaceae), *Romneya* (Papaveraceae), *Ptelea* (Rutaceae, *P. aptera*), *Tetracoccus* (Picrodendraceae, *T. dioicus*), *Xylococcus* (Ericaceae), and endemic species in other genera of succulents such as *Dudleya* (Crassulaceae) and cacti *Cylindropuntia*, *Echinocereus*, *Ferrocactus*, *Mammillaria*, and *Opuntia* (Burge et al. 2016, “Baja CFP”).

Zoo-phylogeographical regions have been classified according to the Great Pacific Fracture Zones (Gottscho 2016).

2. The Chaparral-Desert Ecotone

The flora and vegetation of the Sonoran Desert and the Baja California coastal chaparral, sage and succulent scrub types have been described by Shreve and Wiggins (1964), Wiggins (1980), Turner and Brown (1982), Turner et al. (1995), Peinado et al. (2005, 2011), González-Abraham et al. (2010), Rebman and Roberts 2012; Rebman et al. 2016. A synthesis of the literature on the ecoregions is also provided by González-Abraham et al. (2010), or included within a larger regional classification by Peinado et al. (2008, 2012). Taxonomy and nomenclature of vascular plants names cited herein are according to Rebman et al. (2016), available online, a dedicated website (http://bajaflora.org/).

Our collections in Baja California were largely from coastal areas within three ecoregional subdivisions: (1) 150 from the chaparral-desert transition (Shreve 1936 ; Spjut 1996 ; Riemann and Exequiel 2007 ; Vanderplank 2011 ; Delgadillo 2013 mentioned the “Matorral Costero Suculento de transición”), (2) 113 from the Northern Vizcaíno Desert (Shreve and Wiggins 1964 ; Spjut 1996, or “Central Desert” in Rebman and Roberts 2012), and (3) 165 from the Southern Vizcaíno Desert (Spjut 1996 ; or Vizcaíno Desert in Peinado et al. 1995, Riemann and Exequiel 2007 ; Rebman and Roberts 2012). The nomenclature for these ecoregions in recent classifications may have other names to perhaps differentiate them from other coastal vegetation types.

Among nine hotspots of vascular plants recognized on the Baja peninsula by Riemann and Exequiel (2007), two lie along the western coasts where *Niebla* and *Vermilacinia* communities thrive: (1) the so-called mediterranean corridor located along the northwest coast between 29°45’ and 32°00’ latitude with coastal scrub and chaparral and (2) the coastal areas between

27°20' and 28°55' N of the Vizcaíno peninsula and including the region of Santa Rosallilita. A third recognized by Spjut (1996), the Northern Vizcaíno Desert, which lies between the two, is perhaps the most significant as explained below.

3. The Vizcaíno Deserts

Perhaps the most botanically unusual desert in North America is the Vizcaíno Desert as defined by Shreve and Wiggins (1964), and further divided by Spjut (1996) into Northern (Baja California) and Southern Regions (Baja California Sur); the northern with endemic genera related to those on the Mexican mainland and the southern with endemic species related to those in California. Both *Niebla* and *Vermilacinia* have chemotypes endemic to the Vizcaíno Desert region as a whole while each region has its endemic species of these genera. The Vizcaíno deserts of Spjut (1996) corresponds closely to the Central and Vizcaíno deserts of Rebman and Roberts (2012) who further distinguish a Coastal Sage Scrub from the California Chaparral of Spjut (1996) and refer to the Chaparral Desert Transition of low scrub vegetation (Shreve 1936; Rundel 1978; Peinado et al. 1995; Spjut 1996; Delgadillo 2013) as Coastal Succulent Scrub; this can be further divided into coastal succulent strand scrub and coastal succulent inland scrub as seen discontinuously along the coastal highway from near Tijuana to Ensenada. The Vizcaíno Desert sensu Shreve and Wiggins (1964) also encompasses the entire range of the ancestral lineage of *Ramalina menziesii* (Sork and Werth 2014), and the endemic chemotypes of *Niebla* (ß depsidones) and *Vermilacinia* (triterpenes T1 + T2). Here we recognize the Northern Vizcaíno Desert (NVD) more narrowly than outlined by Shreve and Wiggins (1964) and Spjut (1996) as that which lies West of the mid peninsular range, along the western margin of the Alisitos island arc, a late Cretaceous continental-margin (Busby 2004; Alslebena et al. 2007).

The North Vizcaíno Desert (NVD) has not been separately recognized because it seems that the coastal area has not been studied as much as that inland. This is also because the Pacific coast of Baja California is not easily accessible by vehicle south of Campo Nuevo near Punta San Fernando (Alcaraz and Delgadillo 2010; see map in Spjut 1996, Fig. 2a), except for three main unimproved earth roads that lead west from the paved Highway 1 to where each bifurcates and then terminate at the coast. North-south travel along the coast is limited between Punta San Carlos and Punta Cono. Old tracks that follow the coast, that may still exist, are difficult to navigate as to where they lead. Spjut has collected nearly along the entire coastal NVD, where he travelled by vehicle in a zigzag manner from Punta San Antonio to Punta Santa Rosalillita (from 1985 to 1996). This afforded him the opportunity to observe zonal vegetation patterns along the NVD coast to be closely associated with angiosperm genera endemic to the region. Additionally, portions of the coastal areas were only accessible by foot such as between Punta Canoas and Punta San Carlos where he discovered an unusual alliance of *Mobergia calculiformis* in a lava field of dark basalt just above Punta Escarpada (Fig. 1A); the species had been reported only from Isla Guadalupe and one location in the Sierra de la Giganta of Baja California Sur (Mayrhofer et al. 1992; Spjut 1995). Other unusual lichens discovered within this coastal region are *Niebla tessellata* (Fig. 1B) and *Vermilacinia vesiculosa* on coastal steep rock faces of Mesa Camacho northeast of Punta Canoas; the former species also found on Mesa San Carlos.

The flora of the NVD is remarkable for its monotypic and oliogotypic endemic or near endemic genera of angiosperm coastal shrubs—*Acanthogilia* (Polemoniaceae), *Harfordia* (Polygonaceae, *H. macroptera* var. *galioides*, NVD; var. *fruticosa*, Is. Cedros; var. *macroptera*,

Bahía Magdalena), *Prosopidastrum* (Caesalpinioideae), and *Xylonagra* (Onagraceae, two subspecies, ssp. *wigginsii*, NVD from near Punta Canoas to near Punta Santa Rosalillita with isolated disjunct occurrence near Sierra San Francisco and Punta Eugenia), SVD; ssp. *arborea*, Is. Cedros). As indicated above, their species contribute to zonal vegetation types related to the moderating effects of the Pacific Ocean on the desert climate (Spjut 1996); for example, *Harfordia*, *Prosopidastrum*, and *Xylonagra* were most often observed within 10 km of the coast, and *Acanthogilia* from 10–20 km from the coast. Similar patterns have been reported further north for "phytosociological species associations" in the transitional "coastal succulent scrub region" (Alcaraz and Delgadillo 2010), while it may be noted that lichen zonal patterns have also been recognized along the entire length of the Baja peninsula (Nash et al. 1979; Spjut 1996). The NVD region also includes more widely distributed peninsular endemic or near endemic species of large shrubs, trees, and columnar cacti, exemplified by *Fouquieria columnaris (*Fouquieriaceae), *Pachycorums discolor* var. *pubescens* (Anacardiaceae), *Viscainoa geniculata* (Zygophyllaceae), *Pachycereus pringlei* (Cactaceae), *Myrtillocactus cochal* and *Stenocereus gummosus*—that are most common in the Central Desert Floristic Region (Turner et al 1995; Rebman and Roberts 2012; SEINet, accessed 2019).
The NVD genera that are either endemic, or near endemic, or with disjunct geographical occurrences elsewhere, are phylogenetically basal, or nearly so, to related genera in their respective families or subfamilies. Examples are:

- *Acanthogilia*, resolved within a basal clade of three genera, subfamily Cobaeoideae, sister to the remaining clade of Polemoniaceae (Prather et al. 2000), or unresolved polytomy at the base of the Polemoniaceae (Johnson et al. 2013);
- *Harfordia* (Polygonaceae), in basal lineage of two genera, tribe Pterostegieae, (Kempton 2012), dated 25 Myrs (Schuster et al. 2013), which may be nested within a larger clade (Sanchez et al. 2009);
- *Xylonagra*, monophyletic in basal polytomy, or sister to the tribe Onagreae (Levin et al. 2004), dated 15 Myrs (Sytsma et al. 2004);
- *Fouquieria columnaris*, monotypic in subgenus *Idria*, basal to lineages of 7 species in subgenus *Fouquieria* within a clade sister to subgenus *Brunonia* of three species (De-Nova et al. 2018), the Fouquieriaceae are endemic to the North American deserts, centered in Mexico (Schultheis and Baldwin 1999); its lineage with *Acanthogilia* split 75.4 Myrs, followed by relatively recent diversification for two subgenera 11.2 Myrs (Rose et al. 2018) or 12.72 Myrs (De Nova et al. 2018) ;
- *Viscainoa*, in a basal lineage of three genera in two clades, tribe Morkillioideae, centered in desert regions of Mexico (Sheahan and Chase 2000), the Zygophyllaceae having diversified in other semi-arid regions of the world (Beier et al. 2004);
- and finally *Prosopidastrum*, one species in NVD and 4 species in Argentina (phylogeny unresolved in Caesalpinioideae: Azani et al. 2017). Molecular dating of 30 species in non-monophyletic *Prosopis* from all sections, the genus distributed in arid and semi-arid regions worldwide—using *Prosopidastrum angusticarpum*, an Argentine species as an outgroup, indicated that divergence among the oldest Sections, *Strombocarpa*, *Algarobia* and *Monilicarpa*, occurred in the Oligocene (Burgardt and Espert 2007; Catalano et al. 2008). *Prosopidastrum mexicanum* was also shown to be sister to *P. angusticarpum*, nested in *Prosopis*, with estimated Oligocene age of 33–26.2 Myrs (Catalano et al. 2008).

Of 17 genera of angiosperms recognized strictly endemic to Baja California, five (*Acanthogilia*, *Cochemiea*, *Harfordia*, *Pachycormus*, and *Xylonagra*) occur in the NVD; two additional, ×*Myrtgerocactus* and ×*Pacherocactus*, are known only from the vicinity of El Rosario, which

lies at the northern fringe of the NVD, and *Hesperelaea*, known only from Isla Guadalupe, considered extinct. This type of paleoendemism concentrated within a relatively small area (NVD) seems unusual and requires attention. Lineages that date from 12 to 25 Myrs or more, would seem to indicate that the NVD was isolated from the mainland, perhaps as an island during the late Eocene to Oligocene. Although we did not collect in the Magdalena Region, this region also has paleoendemic genera represented by *Gongylocarpus* with *G. fruticulosus*, ssp. *fruticulosus* (Onagraceae) endemic to Is. Santa Margarita, and ssp. *glaber* endemic to Is. Magadalena. A single *Gongylocarpus* lineage, sister to a clade with *Xylonagra* and several other genera, is shown to be c. 40 Myrs (Berger et al. 2016). One other species in the genus is herbaceous and occurs on mainland Mexico.

A similar anomaly of paleoendemism in the central Chile flora has been described by Rundel et al. (2016). The flora is reported to be relatively small with c. 2,900 species. Numerous herbaceous lineages known elsewhere in Chile are absent as also the case for the paucity of *Eriogonum* species in the NVD mentioned below under the SVD. Moreover, many woody clades predate the establishment of the MTC [Mediterranean-type Ecosystems] in the middle Miocene. Gondwanan lineages with Australasian linkages that split very early can be seen in *Lomatia*, the monotypic *Gevuina* (Proteaceae) (Barker et al. 2007) and the monotypic *Peumus* (Monimiaceae) (Renner et al. 2010). Other groups of Gondwanan origin include *Caldcluvia* (Cunoniaceae) (Bradford and Barnes 2001), *Eucryphia* (Eucryphiaceae) (Taylor and Hill 1996), *Fuscospora* (Nothofagaceae) (Sauquet et al. 2012), and the monotypic Gomortegaceae and Aextoxicaceae. Indeed, Tertiary fossils of *Lomatia* have been reported from Patagonia in Eocene (*L. occidentalis* and *L. preferruginea*), and Late Eocene-Early Oligocene deposits (*L. patagonica*) where the species probably arrived via the Australia-Antarctica-South America connection (Gonzalez et al. 2007). *Eucryphia* fossils of three species are reported from Australian Paleocene and Eocene deposits (Hill 1991). *Fuscospora*, a segregate genus of the well-known *Nothofagus*, with a rich fossil record from Australia, New Zealand, Antarctica, and South America, extends into the late Cretaceous (Hill 2001).

Vicariance patterns in the peninsular biota of Baja California have been suggested to be the result of relatively recent mid-peninsula seaways; for example, Hafner and Riddle (2011) show that Baja California peninsula as one of five islands 7–5 Myrs; the others are the Vizcaíno Peninsula, Magdalena Island, and the Cape Region that in turn was separated from the main peninsula of Baja California Sur; however, during that time the peninsula was scarcely separated from the mainland. Thus, the late Miocene putative islands would appear too recent and short-lived to account for the NVD ancestral endemic genera, while they could possibly account for phylogeny within *Niebla* since we suggest that the genus is undergoing relatively rapid speciation compared to that of *Ramalina* and *Vermilacinia*. Additionally, submergence of low elevated areas in the western Vizcaíno Peninsula would lead to sea transgression, resulting in isolation of the elevated areas as islands, especially during interglacial periods, and before the Pleistocene. Sea surface temperatures off the California coast were still several degrees warmer than today during the early Pliocene warm period, beginning 4.6 Myrs and ending c. 3 Myrs with the onset of northern hemisphere glaciations (Dekens et al. 2007).

The Southern Vizcaino Desert

The Southern Vizcaíno Desert (SVD) has mixed desert and chaparral-coastal sage vegetation in the mountainous areas of the western Vizcaíno Peninsula (Spjut 1996). SVD vegetation associations described by Peinado et al. (2005) are of the widely distributed desert and alkali scrub species. The SVD endemic species, on the other hand, are related to species thriving in California (Spjut 1996). Examples are *Rhus lentii*, *Salvia cedrosensis*, *Eriogonum encelioides*, *E. preclarum*, *E. repens*, *E. scalare*, *Encelia asperifolia* (Is. Cedros), *E. ventorum* and *Penstemon vizcainensis*. The latter *Encelia* notably differs from other brittle-bush *Encelia* spp. by its arborescent habit and deeply dissected leaves. The California coastal *Rhus integrifolia* extends south to near El Rosario. The genus *Eriogonum* recognized to have 53 taxa in Baja California, 29 of which are endemic (Rebman et al. 2016), is virtually absent from the NVD. This void emphasizes the distinction of the SVD as well that of the NVD. Examples of disjunct species between the California chaparral/Sonoran Desert and SVD are *Petalonyx linearisi* (Turner et al. 1995; SEINet) and *Ziziphus parryi*, the California species var. *parryi* occurs along the western edge of the Sonoran Desert to near the Mexican border, and var. *microphylla*, reported from Isla Cedros, and near El Rosario (Rebman et al. 2016), has also been discovered by Spjut on Mesa Camacho in the NVD, Apr 1994 (Spjut & Marin 13085, US; image of specimen also www.worldbotanical.com.) where occurring just below the crest in a narrow rocky ravine among a diverse *Niebla* flora.

(3) *Niebla* and *Vermilacinia* Communities of Baja California

Spjut (1996) classified *Niebla* communities by their substrate such as gravel, sand, pebble, boulder, and mixed. The communities also have ecogeographic and-morphological-chemical relationships as further summarized below:

- *Niebla* growing on gravel form terricolous communities in the chaparral-desert ecotone where *Niebla* growth initiates from soil between gravel (Plate 2B, Spjut 1996). More than 80% of the ground cover between shrubs spaced at c. 1 m intervals is terricolous *Niebla* (see also Rundel et al. 1972). Thalli are predominantly mats—to more than 1 m in diam (Spjut 1996, Plate 4C-E), and usually contain salazinic acid. Rare chemotypes are protocetraric acid, known from San Antonio del Mar and Bahía de San Quintín; divaricatic acid rare at San Antonio del Mar, and sekikaic acid infrequent on rolling hills southwest of El Rosario and mesa above San Antonio del Mar, and Isla Coronado. Also, terricolous thalli occur on San Nicolas Island where represented by two endemic species, one prostrate with divaricatic acid (*N. ramosissima*), the other erect with sekikaic acid (*N. dactylifera*).
- *Niebla* growing on sandy soil (Plate2C, Spjut 1996) are generally more hemispherical and less wind-swept in appearance than gravel *Niebla*'s, are often encountered around tidal flats, mud flats, clay-pans, and sandy margins of arroyos just inland from the sea or bays, from near San Antonio del Mar to northern shore of the Vizcaíno Peninsula. The thalli usually contain salazinic acid. Rare chemotypes for sand substrates are sekikaic acid thalli collected near volcanic cones on the peninsula west of San Quintín, and hypoprotocetraric acid thalli near Punta Canoas and vicinity of Morro Santo Domingo in the central and southern NVD.
- *Niebla* growing on pebbles generally develop thalli of tufted basal branches not more than 6 cm high—and confined to the stone upon which they grow (Plate 2D, Spjut 1996); they occur over extensive areas where fog develops along the Pacific Coast in the NVD. At their northern range near Campo Nuevo just south of Punta San Fernando, their occurrence is associated with the change in vegetation from the chaparral-desert ecotone

to the NVD. Not all stones have *Niebla*, which may be conspicuously absent on relatively large rocks. Both divaricatic acid and salazinic acid thalli can be equally present with occasional sekikaic-acid thalli, or one chemotype may dominate. On the Vizcaíno Peninsula a close association exists between salazinic acid and hypoprotocetraric acid thalli on small conglomerate and limestone rocks, with rare occurrences of neither acid present (acid deficient).

- "Boulder" *Niebla* grow on irregularly shaped large rocks, often volcanic, in contrast to the small polished stones seen on beaches for pebble-*Niebla*. Thalli vary from small tufts to bush-like in habit (Plate 2E-F, Spjut 1996). Small rocks in areas with boulders are usually devoid of *Niebla*. Thalli with divaricatic, sekikaic, and salazinic acids may equally occur, or one may dominate, especially near the northern and southern range of the genus. From general observations, sekikaic acid occurs more frequently inland or on the drier side of rocks, especially volcanic rocks (see also Rundel et al. 1972), and also more commonly collected in the Channel Islands (Spjut 1996, *N. dactylifera*, *N. disrupta*, *N. dissecta*, *N. fimbriata*, *N. siphonoloba*).
- Mixed *Niebla* communities occur on sand, small rock and large boulders, often where habitat is diverse. Mixed communities also include *Vermilacinia* thalli growing with *Niebla*, which is usually dominant, or they may share the same holdfast, e.g. *Niebla homaleoides* (acid deficient) with *Vermilacinia paleoderma* (Spjut 1996, Photo 17.2).

Vermilacinia communities include epiphytic species on various shrubs along the coasts of southern California and Baja California; for example, *V. leopardina* may cover 100% of all branches of shrubs, such as *Euphorbia misera*, *Fouquieria diguetii* (Rundel et al. 1972; Enzien and Margulis 1988, and *Lycium* spp. (Alcarez and Delgadillo 2010). In the Magdalena Region, corticolous *Vermilacinia* often grows mixed with *Ramalina* spp. (Spjut 1996, Plate 11F), or closely entangle as photographed for *R. crinita* (see Fig. 1 below). *Vermilacinia* can dominate rocks near ocean mist and on coastal ridges near the sea. Saxicolous *Vermilacinia* communities are particularly common between Punta Negra and Punta Santa Rosalillita, and on the Vizcaíno Peninsula near Punta Eugenia.

A detailed assessment of lichen species cover among the overall vegetation was made by Rundel et al. (1972). They reported percentages for ground cover of lichen species on a peninsula near San Quintín, focusing on the genus *Niebla*, referred to at that time by the illegitimate name "*Desmazieria*". They determined, for example, that *Niebla josecuervoi* (salazinic acid) and *Niebla* depside species together covered 10–75% of the "rocky substrate," in which the density increased with increase in elevation on Colima del Sudoeste. At lower elevation, 10 m from the base of the volcanic cone, they reported sekikaic acid and salazinic acid thalli collectively covered 25% of the rock substrate, compared to 60% at 50–70 m further up slope where divaricatic acid thalli replaced sekikaic acid thalli in association with salazinic acid thalli (*N. josecuervoi*).

Nearby—at the base of the cone on sand, among scattered shrubs—Rundel et al. (1972) reported terricolous thalli with sekikaic acid (*N. palmeri* sensu Spjut) were <2% in ground cover, whereas collectively with salazinic acid thalli (*N. arenaria* sensu Spjut) cover was 25% on a sandy substrate at another site. Additionally, "*Desmazieria pulchribarbara*" Rundel & Bowler (type with protocetraric acid), was distinguished morphologically as a new species by its terricolous habit and by absence of apothecia, in contrast to their new saxicolous

"*Desmazieria josecuervoi*" Rundel & Bowler producing apothecia. Terricolous *N. pulchribarbara* was inferred to be the primary ground cover growing "abundantly on sandy flatlands surrounding the false bay of Bahía de San Quintín," the type locality for the species and also for Spjut's (1996) *Niebla arenaria*; however, Spjut distinguished *N. arenaria* from *N. pulchribarbara* by having salazinic acid instead of protocetraric acid. *Niebla* containing only protocetraric acid is extremely rare; two additional collections were later reported from Bahía de San Quintín, and another two from a mesa above San Antonio del Mar (Spjut 1996) where subsequently recollected by Spjut and Sérusiaux (17015, Jan 2016), its phylogeny unresolved. Both *N. arenaria* and *N. pulchribarbara* (sensu Spjut 1996) are treated as synonyms of *N. josecuervoi* by Bowler and Marsh (in Nash et al. 2004). It should be noted that the description of *N. homalea* in the Greater Flora of the Sonoran Desert by Bowler and Marsh (Nash et al. 2004)—reporting to contain protocetraric acid—is incorrect.

Alcaraz and Delgadillo (2010), in their study of coastal vegetation in the region of "Valle de los Cirios," two sites in the Chaparral-Desert-Ecotone and three in the NVD, they mentioned *Vermilacinia leopardina*, specifically, in their description of "*Ferocacto fordii–Euphorbietum miserae* association," to totally cover the branches of the shrubs, Frankenia *palmeri* and *Euphorbia misera*, supported by photo in their Fig. 7. Among the halophytic ground vegetation, they note the abundance of terricolous *Niebla arenaria* in their Fig. 14, and in a photo of a pebble lichen community, an association of *Niebla podetiaforma* and *Vermilacinia paleoderma* was presented [citing Spjut (1996) and his web pages on the species and genera of *Niebla* and *Vermilacinia* (2003) and the "*Niebla* and *Vermilacinia* Communities" (2005)].

Epiphytic *Niebla* "*ceruchis*" (= *Vermilacinia leopardina*), occurring with other lichen species such as *Dendrographa leucophaea*, have been reported by many authors to cover shrubs, "in a mass thick enough to mask the identity of the substrate species (Rundel et al. 1972)." Peinado et al. (2005) also mentioned: « *Niebla* » species(= *Vermilacinia*) hung densely from stems of *Lycium* […]».

(II) The Atacama and Sechura deserts

The contiguous Atacama and Sechura deserts of South America represents one of the harshest and driest environments on earth (Arroyo et al 1988; Schulz et al 2012) and harbors an impressive list of endemic plant and animal species (Rundel et al 1991 ; Vidal et al 2009). This desert extends for >3,500 km from 5°S near the Peruvian–Ecuadorean border to 30°S in northern Chile. In the Atacama region, arid climates (precipitation of ≤50 mm/y) extend from coastal regions from 5°S to 30°S up to 5,000 m (8); hyperarid climates (≤5 mm/y) extend from 13°S to 25°S, from coastal areas to 3,000 m (Garreaud et al 2009). Whereas the onset of semiarid conditions (<250 mm/y) in the Atacama– Sechura region is detected in the late Jurassic (Hartley et al 2005), arid conditions (<50 mm/y) have prevailed in this region since the early Oligocene (33 Myrs) (Dunai et al 2005); the minimum age of onset of hyperarid climate in the region is around 8 Myrs, in the late Miocene (Schlunegger et al 2010), although some evidence suggests that pulses of hyperaridity could be older (Rech et al 2006).

These deserts owe their severe aridity to a climatic regime dominated by a constant temperature inversion generated, in large part, by the cool, north-flowing Humboldt Current.

Also important is the influence of strong atmospheric subsidence associated with a positionally stable, subtropical anticyclone. The result is a mild, uniform coastal climate with the regular formation of thick stratus cloud banks below 1000 m during the winter months. Where coastal topography is low and flat, this stratus layer dissipates inward over broad areas with little biological impact. However, where steep coastal slopes are present, this stratus layer forms a fog-zone concentrated against the hillsides." These fog oases, also known as « Loma » or « Nebeloase » "are the key to the extent and diversity of vegetation in the Atacama and Peruvian Deserts" (Rundel et al. 1991). Redón and Lange (1983) recognized two main lichen communities whose species were distributed among five elevation zones, the *Oropogonetum loxensis* growing on shrubs and trees in the zone of persistent fog, and the *Ramalinetum cactacearum* on shrubs below the fog belt, particularly in zones 2 and 3, 200–300 m elev. *Ramalina cactacearum* was regarded by Spjut (1996) as a possible synonym of *Vermilacinia flaccescens*, differing in the lack of terpenes that are usually found with tumidulin in that species. In zone 5, other *Vermilacinia* species identified *R. ceruchis* and *R. tumidula* are typically terricolous; their reference to growing on shrubs suggest possibly *V. tigrina*.

Guerrero et al (2013) selected three plant genera (*Chaetanthera*, Asteraceae; *Malesherbia*, Passifloraceae; *Nolana*, Solanaceae) and one animal genus (*Liolaemus*, Liolaemidae) and tested the date of their local radiation in the Atacama and Sechura deserts, based on molecular phylogenies. Interestingly they demonstrated that three of them invaded arid regions of the Atacama–Sechura Desert in the last 10 million years, some 20 million years after the initial onset of aridity in the region. Further, the most diverse plant clade in these habitats (*Nolana*) only colonized them ∼2 Myrs, except *N. onoana*, 3.8 Myrs. This pattern is coherent with other emblematic groups of the New World such as the Cactaceae as this iconic family originated shortly after the Eocene–Oligocene global drop in CO_2, and radiation of its richest genera began in the late Miocene (Hernández-Hernández et al 2014). The Zygophyllaceae further provides support for this pattern in the cumulative evidence that indicates a rise of dryland floras across all continents closely-synchronized with the mid-late Miocene (Wu et al 2018). Nevertheless, paleoendemic genera also persisted in the Atacama Desert; their ancestral relationships date back to the Cretaceous as decribed earlier for the NVD in Baja California with reference to Rundel et al. (2016).

The lichen flora of the Atacama-Sechura deserts is known from descriptions of several new genera and species, from local check-lists, and from ecological assessements, especially the so-called fog oasis, areas affected by orographic accumulation of fog. Examples are Follmann (1967, 1997, 2008); Follmann and Peine (1999) ; Peine and Werner (1995) ; Sipman (1995) ; and Santiago et al (2018). Follmann and Redón (1972) reported 184 taxa belonging to 73 genera from such habitats; Follmann (1994) after « various collecting trips to the Province of Iquique » in search of « Darwin Oases, 1960-1990» reportedly «residscovered» the « Darwin lichen oasis » near Iquique (Chile).

Examples of fog lichen genera found among the fog deserts of Atacama, North America, and Namibia were published by Rundel (1978). Relationships based on molecular data have since been provided for some. Examples are *Gymnographopsis chileana* in the Atacama in northern Chile and *G. latispora* in South Africa/Cape area (Lücking et al. 2013). *Combea mollusca*,

endemic to the Namib and sister to the monotypic *Dolichocarpus chilensis*, endemic to the Atacama desert (Ertz and Tehler 2011).

Niebla sensu Spjut (1996) does not occur in the Atamaca Desert including two corticolous species described by Sipman (2001) in *Niebla* from there. One, *N. granulans*, is similar in habit to *Vermilacinia zebrina* but differs by the apical granular punctiform soralia and by containing the depside methyl 3,5 dichlorolecanorate (tumidulin) along with four terpenoids associated with a «bloom in herbarium specimens » that may be attributed to the diterpene [-]-16 α-hydroxykaurane, known also as ceruchdiol (Bendz et al. 1965) or ceruchinol (Follmann 1966). Clearly, *N. granulans* belongs to *Vermilacinia* as also recognized by Sipman (2011); thus, we propose the new combination *Vermilacinia granulans* (Sipman) Spjut & Sérusiaux. The other, « *Niebla nashii* », also reported with tumidulin and two associated terpenes but without « bloom , » differs by its somewhat foliose habit, similar to *Ramalina lacera* in appearance except for its much smaller size, and by the absence of both pycnidia and apothecia, and by its relatively thin cortex, absent on the lower surface on apical lobes where ± sorediate; it may belong to an undescribed genus along with other related tumidulin containing specimens mentioned in Spjut (1996) under *V. flaccescens*. An unresolved group of corticolous species (*V. howei, V. leopardina, V. nylanderi*)— similar to *V. tigrina* described by Follmann (1966, *Ramalina tigrina*) from Cerro Moreno in Chile—occur in the California regions, and also reported from both American regions are *V. cerebra* and *V. leonis* described from Baja California and Baja California Sur. Similar, or the same, saxicolous *Vermilacinia* species shared among the American deserts include *V. acicularis*, *V.* aff. *ceruchoides*, *V.* aff. *procera*, *V.* aff. *robusta*, and *V.* aff. *varicosa* as determined from review of lichen specimens collected from the Atacama Desert (loan from BYU to WBA, 2016) and Spjut (1996). Additionally, «variants » of the endemic *V. ceruchis* described and shown in Spjut (1996) appear endemic to the Atacama-Sechura deserts.

Several other genera are assumed to be endemic to the area, mostly in the Roccellaceae (sensu Follmann 2001, 2002), as 11 genera and 51 species belonging to that family are reported and all species and many genera are endemic to the area. The family is indeed very diverse (Ertz and Tehler 2011; Ertz et al 2014, 2015; Tehler et al. 2013) but many genera considered as endemic to the Atacama-Sechura deserts have not yet been examined with molecular inferences in a phylogenetic context.

Finally, the fruticose genus *Santessonia* (Caliciaceae sensu Wedin et al 2000), well-adapated to rapidly capture moisture from fog through its lacunose cortex (Sérusiaux and Wessels 1984), was first recognized to have three species endemic to the Namib desert; later three species in the Atacama Desert were added (Follmann 2006). However, molecular inferences optimized in a phylogenetic concept are needed in view of possible homoplasy (Larraín-Barrios et al 2018).

(III) The Coasts of Namibia and South-West of South Africa

The post-Gondwana (i.e. after the super-continent broke apart) record of the Namib was reviewed by Ward and Corbett (1990) and a revised model for its history was divided into five major events. The last two identify the steps that concern the implementation of persistent coastal fogs: a « Pedogenic phase » in the Middle Miocene (14-11 Ma), heralding the onset of

arid conditions attendant on the full establishment of the cold-water upwelling system of the Benguela Current in the Late Miocene (10-7 Ma); and the « Namib Desert phase » which has persisted from the Late Miocene (10-7 Ma) to the present day. The Benguela Current is well-known (Heinrich et al. 2011; Rommerskirchen et al. 2011; Jung et al. 2014).

The Greater Cape Floristic Region (GCFR; Born et al., 2007) is exceptional in terms of its floristic endemism and species richness (Rafiqpoor et al 2005; Linder 2014) with two very species-rich elements: the Cape flora in the southwest and the Succulent Karoo in the west. The flora of the Cape, with c. 9000 species within an area of 90000 km^2 is extraordinarily speciose and its strict endemism is almost 70%; this richness is the result of recruiting diverse lineages over the entire Cenozoic (Linder 2005), or at least since the early Oligocene (Schnitzler et al. 2011), while 11 radiations also account for many endemic species since the Miocene (Linder 2003). By applying palynological and biogeochemical methods to marine material off the mouth of Orange river on the Atlantic coast of SW Africa, Dupont et al (2011) unambiguously linked the floral development on the continent to the oceanic development of the BUS (Benguela Upwelling System) and the climate changes associated with it. According to the detailed study by Rommerskirchen et al (2011), the BUS was established at c. 10 Myrs; however, it may be noted that a prototype Benguela was already present by early Oligocene (Kelly et al. 2011). The same applies for the Succulent Karoo, a small area along the south-western coast of southern Africa and much renowned for its extraordinary radiation of the Aizoaceae (Cowling et al.; 1999; Mucina et al. 2006; Klak et al 2004). As far as our review of literature could tell, no Pliocene or Quaternary interruption of the BUS has been recorded, therefore, no interruption of the coastal fog zone. However, Rommerskirchen et al (2011) established that: “During the Messinian Salinity Crisis, between 7 and 5 Myrs, surface and subsurface temperature estimates became similar, likely because of a strong reduction in Atlantic overturning circulation, while high total organic carbon contents suggest a “biogenic bloom ».

Although many authors have studied the lichen flora along the Namib coasts, the total flora is not impressive, probably c. 100 species (Wirth 2010b). Many are endemic, however, if one includes the coasts of Namibia southwards down to the Cape area. Their taxonomy is better known (ex.: Egea et al 1997; Kärnefelt 1988 ; Wirth and Elix 2006). The flora includes two monotypic endemic genera: *Combea*, a monotypic genus resolved into the Opegraphaceae (Arthoniomycetes), following molecular inferences (Ertz and Tehler 2011) and *Coronoplectrum*, of uncertain phylogenetic affinities but clearly belonging to the Lecanoromycetes (Brusse 1987). Further, very characteristic subfoliose species are easily detected by their lobes exhibiting a unique morphology amongst all lichens (*Stellarangia elegantissima* and *S. namibensis*; Kärnefelt 1988 ; Arup et al 2013) and spectacular and conspicuous foliose species assigned to the world distributed genus *Xanthoparmelia* : *X. hottentota* with a single holdfast and large, maculate and ciliate lobes that open like a flower during periods of fog and close when the air becomes dry and warm again ; and the vagrant *X. convoluta* with enrolled cigarette-like thallus. The phylogenetic affinities within the genus are extremely complex and still unresolved (Leavitt et al 2018). Also of interest are the different adaptations to soil conditions in the desert with the so-called window lichen and inverted one (Vogel 1955; Büdel and Schultz 2003; Green et al 2018).

The lichen communities in the Namib desert occur locally and are well-known (Schieferstein and Loris 1992; Lalley and Viles 2005; Lalley et al 2006; Wirth 2010a). Advanced photogrammetry techniques conducted in central Namibia identified percentages of lichen susbtrate cover for “an orange *Stellarangia* spp. assemblage” of 22.8% and a “black *Xanthoparmelia* spp. assemblage” of 0.6% (Hinchliffe et al. 2017).

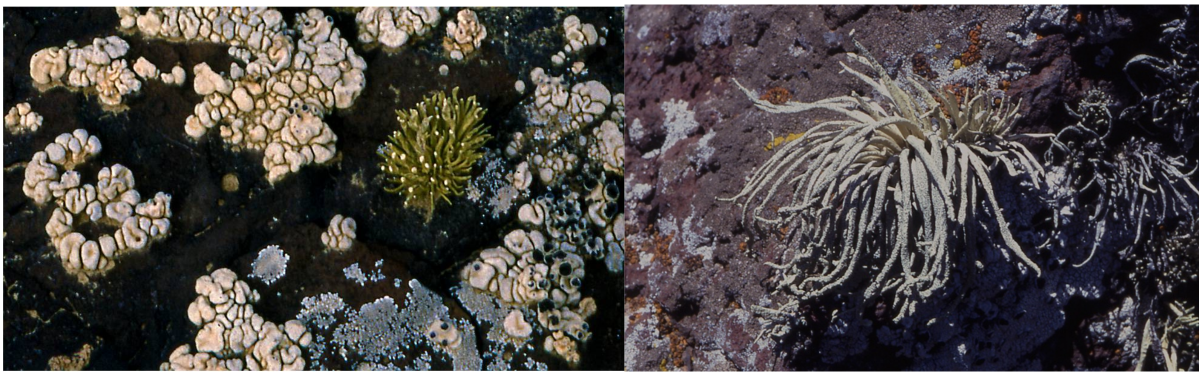

Fig. 1. Rare lichens in the Northern Vizcaíno Desert, between Punta Canoas and Punta San Carlos: A (left) *Mobergia calculiformis* just above Punta Escarpada; B (right) *Niebla tessellata*. (Photographs R. Spjut)

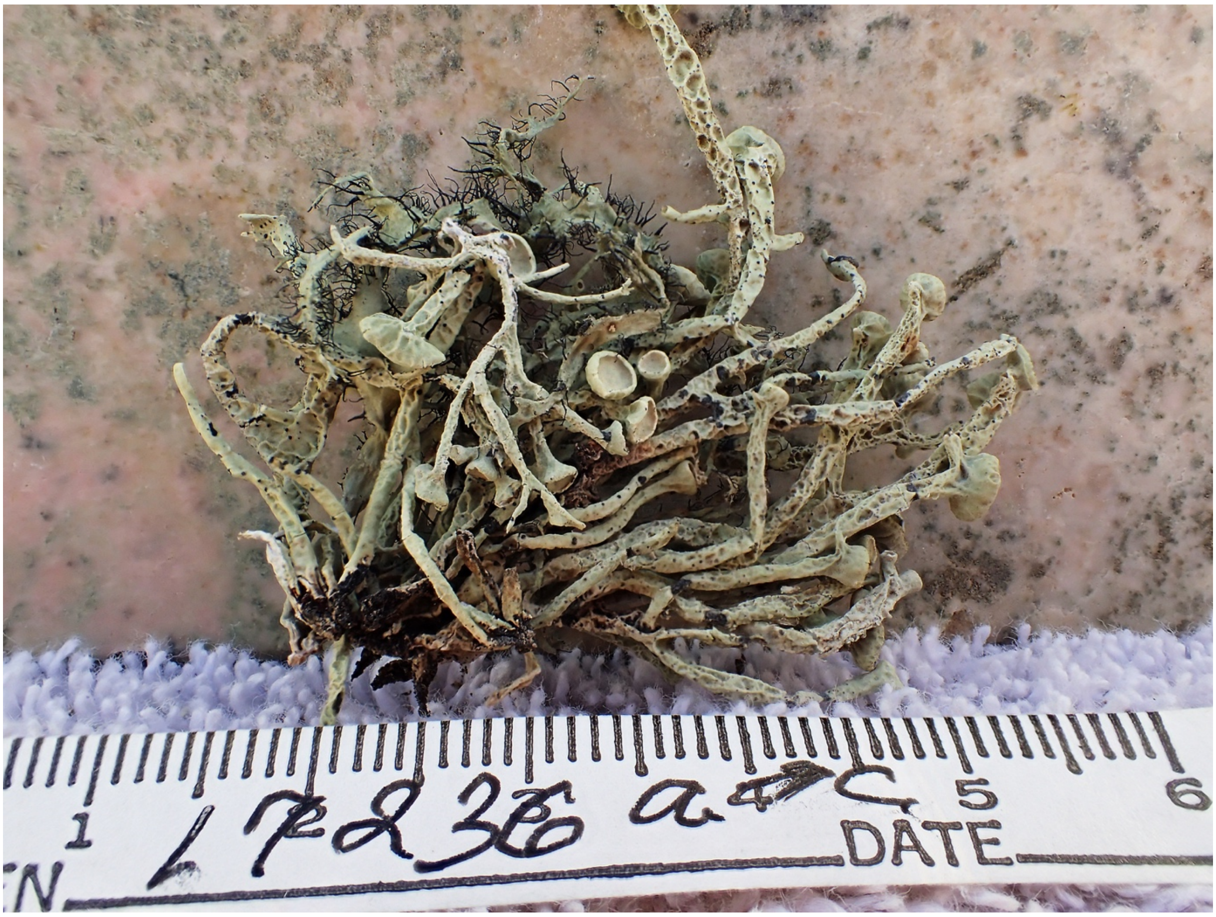

Fig. 1: Entangling of *Ramalina crinita* and *Vermilacinia* sp. (Spjut & Sérusiaux 17236B).

Literature

- Alcaraz F, Delgadillo J (2010) Matorrales succulentos y desérticos del desierto central de Baja California. Braun-Blanquetia 46: 85-89.
- Alslebena H, Schmidt K, Wetmoe PH, Peterson SR (2008) Complex deformation during arc–continent collision: Quantifying finite strain in the accreted Alisitos arc, Peninsular Ranges batholith, Baja California. Journal of Structural Geology 30(2):220-236.
- Arroyo MTK, Squeo FA, Armesto JJ, Villagrán C (1988) Effects of aridity on plant diversity in the northern Chilean Andes: Results of a natural experiment. Annals Missouri Botanical Garden 75: 55–78.
- Arup U, Søchting U, Frödén P (2013) A new taxonomy of the family Teloschistaceae. Nordic Journal of Botany 31(1): 16-83.
- Atwater T (1970). Implications of Plate Tectonics for the Cenozoic tectonic evolution of western North America. Geological Society of America Bulletin 81(12): 3513-3536.
- Atwater T (1989) Plate tectonics history of the northeast Pacific and western North America. Chapter 4. In Decker RW, Hussong DM, Winterer EL (Eds) The Geology of North America. The eastern Pacific Ocean and Hawaii, Geologic Society of North America, 21–72.
- Axelrod DI (1967) Geologic history of the California insular flora. In: Philbrick RN (Eds) Proceedings of the symposium on the biology of the California Islands. Santa Barbara Botanical Gardens, Santa Barbara, California, 267–316.
- Axelrod, D. I. 1979. Age and origin of the Sonoran Desert. California Academy of Sciences, Occasional Papers No. 132, 1-74.
- Azani N, Babineau M, Bailey CD, Banks H, Barbosa AR, Pinto RB, [...], Candido E « The Legume Phylogeny Working Group (LPWG) » (2017) A new subfamily classification of the Leguminosae based on a taxonomically comprehensive phylogeny. Taxon 66(1) : 44-77.
- Barak S, Klemperer SL, Lawrence JF (2015) San Andreas Fault dip, Peninsular Ranges mafic lower crust and partial melt in the Salton Trough, Southern California, from ambient-noise tomography. Geochemistry, Geophysics, Geosystems 16(11): 3946-3972.
- Barker NP, Weston PH, Rutschmann F, Sauquet H (2007). Molecular dating of the 'Gondwanan' plant family Proteaceae is only partially congruent with the timing of the break-up of Gondwana. Journal of Biogeography 34: 2012–27.
- Baldwin BG, Thornhill AH, Freyman WA, Ackerly DD, Kling MM, Morueta-Holme N, Mishler BD (2017) Species richness and endemism in the native flora of California. American Journal of Botany 104(3): 487-501.
- Beier BA, Nylander JAA, Chase MW, Thulin M (2004) Phylogenetic relationships and biogeography of the desert plant genus Fagonia (Zygophyllaceae), inferred by parsimony and Bayesian model averaging. Molecular Phylogeznetics and Evolution 33(1): 91-108.
- Bendz GJ, Santesson CW, Wachtmeister (1965) Studies on the chemistry of lichens. 20. The chemistry of the Ramalina ceruchis group. Acta Chemica Scandinavica 19:1185-1187.
- Berger BA, Kriebel R, Spalink D, Sytsma, KJ (2016) Divergence times, historical biogeography, and shifts in speciation rates of Myrtales. Molecular Phylogenetics and Evolution 95: 116–136.
- Blakey RC, Ranney W (2018) Ancient Landscapes of Western North America: A geologic history with paleogeographic maps. Springer, 1-228.
- Born J, Linder HP, Desmet P (2007) The Greater Cape Floristic Region. Journal of Biogeography 34 : 147–162.
- Bowler PA, Marsh JE (2004) Niebla. In: Nash III TH, Ryan B, Diederich P, Gries C, Bungartz F (eds), Lichen Flora of the Greater Sonoran Desert Region. Lichens Unlimited, vol. 2. Arizona State University, Tempe, Arizona, 368-380.
- Bradford JC, Barnes RW (2001) Phylogenetics and classification of Cunoniaceae (Oxalidales) using chloroplast DNA sequences and morphology. Systematic Botany 26: 354–85.

- Brusca RC (2019) A brief geologic history of northwestern Mexico. Vers. 7. August 2019, http://www.rickbrusca.com/http/www.rickbrusca.com_index.html/Papers_files/Geology%20of%20NW%20Mexico.pdf.
- Brusse FA (1987) Coronoplectrum, a new lichen genus from the Namib Desert, South West Africa/Namibia. Mycotaxon 28: 131-135.
- Büdel B, Schultz M (2003) A way to cope with high irradiance and drought: inverted morphology of a new cyanobacterial lichen, Peltula inversa sp. nov., from the Nama Karoo, Namibia. Bibliotheca Lichenologica 86 :225-232.
- Burge DO, Thorne JH, Harrison SP, O'Brien BC, Rebman JP, Shevock JR, […], Barry T (2016) Plant diversity and endemism in the California Floristic Province. Madroño 63(2): 3-206.
- Burghardt AD, Espert SM (2007) Phylogeny of *Prosopis* (Leguminosae) as shown by morphological and biochemical evidence. Australian Systematic Botany 20: 332–339.
- Busby C (2004) Continental growth at convergent margins facing large ocean basins: a case study from Mesozoic convergent-margin basins of Baja California, Mexico. Tectonophysics 392: 241 – 277.
- Calflora (2019). *Artemisia californica*. Information on California plants for education, research and conservation, with data contributed by public and private institutions and individuals, including the Consortium of California Herbaria. Berkeley, California: The Calflora Database, https://www.calflora.org/ (Accessed: Aug 12, 2019).
- Calsbeek R, Thompson JN, Richardson JE (2003) Patterns of molecular evolution and diversification in a biodiversity hotspot: the California Floristic Province. Molecular Ecology 12(4): 1021-1029.
- Cowling RM, Esler KJ, Rundel PW (1999) Namaqualand, South Africa – an overview of a unique winter-rainfall desert ecosystem. Plant Ecology 142: 3–21.
- Catalano SA, Vilardi JC, Tosto D, Saidman BO (2008) Molecular phylogeny and diversification history of *Prosopis* (Fabaceae: Mimosoideae). The Linnean Society of London, Biological Journal of the Linnean Society 93: 621–640.
- Davis AS, Clague DA, Paduan JB, Cousens BL, Huard J (2010) Origin of volcanic seamounts at the continental margin of California related to changes in plate margins. Geochemistry, Geophysics, Geosystems 11(5): 1-28.
- Delgadillo Rodríguez J (2013). Flora y Vegetación Manual de Practica de Campo. Ver. 8. Universidad Autonoma de Baja California, Facultad de Ciencias.
- Dekens PS, Ravelo AC, McCarthy MD (2007) Warm upwelling regions in the Pliocene warm period. Paleoceanography 22:PA3211.
- De-Nova JA, Sánchez-Reyes LL, Eguiarte LE, Magallón S (2018) Recent radiation and dispersal of an ancient lineage: The case of Fouquieria (Fouquiericeae, Ericales) in North American deserts. Molecular phylogenetics and evolution 126 : 92-104.
- Dunai TJ, González López GA, Juez-Larré J (2005) Oligocene/Miocene age of aridity in the Atacama Desert revealed by exposure dating of erosion sensitive landforms. Geology 33: 321–324.
- Egea JM, Sérusiaux E, Torrente P, Wessels D (1997) Three new species of Opegraphaceae (lichens) from the Namib Desert. Mycotaxon 61: 455-466.
- Enzien M, Margulis K. (1988) ''Niebla ceruchis'' from Laguna Figueroa: dimorphic spore morphology and secondary compounds localized in pycnidia and apothecia. Microbios 55:75–83.
- Ertz D, Tehler A (2011) The phylogeny of Arthoniales (Pezizomycotina) inferred from nucLSU and RPB2 sequences. Fungal Diversity 49(1): 47-71.
- Ertz D, Tehler A, Fischer E, Killmann D, Razafindrahaja T, Sérusiaux E (2014) Isalonactis, a new genus of Roccellaceae (Arthoniales), from southern Madagascar. The Lichenologist 46(2): 159-167.
- Ertz D, Tehler A, Irestedt M, Frisch A, Thor G, van den Boom P (2015). A large-scale phylogenetic revision of Roccellaceae (Arthoniales) reveals eight new genera. Fungal Diversity 70(1) : 31-53.
- Erwin DM, Schorn HE (2000) Revision of Lyonothamnus A. Gray (Rosaceae) from the Neogene of Western North America. International Journal of Plant Sciience 161(1):179-193;

- Fletcher JM, Grove M, Kimbrough D, Lovera O, Gehrels, GE (2007) Ridge-trench interactions and the Neogene tectonic evolution of the Magdalena shelf and southern Gulf of California: Insights from detrital zircon U-Pb ages from the Magdalena fan and adjacent areas. Geological Society of America Bulletin 119: 1313–1336.
- Follmann G (1966) Eine neue Ramalina-Art uas der Ceruches-Gruppe. Willdenowia 4: 225–233.
- Follmann G (1967) Die Flechtenflora der nordchilenischen Nebeloase Cerro Moreno. Nova Hedwigia 14: 215-281.
- Follmann G (1994) Darwin's "lichen oasis" above Iquique, Atacama Desert rediscovered. International Lichenological Newsletter 27(2): 23-24.
- Follmann G (1997) Hubbsia langei, a new Roccellaceae from the Atacama Desert, north Chile, and the identity of two Reinkella species. In: Kappen L (Ed.) New Species and Novel Aspects in Ecology and Physiology of Lichens. In Honour of O. L. Lange. Bibliotheca Lichenologica, J. Cramer, Berlin, Stuttgart, 11-24.
- Follmann G (2001) An integrated key to, and a critical survey of the South American representatives of the lichen family Roccellaceae (Arthoniales). Journal of the Hattori Botanical Laboratory 90: 251-267.
- Follmann G (2002) South America as diversity centre of the lichen family Roccellaceae (Arthoniales). Mitteilungen aus dem Institut für Allgemeine Botanik Hamburg 30-32 : 61-67.
- Follmann G (2006) Two new roccelloid Physciaceae from pacific South America and a synopsis of the genus Santessonia. The Journal of the Hattori Botanical Laboratory 100: 651-670.
- Follmann G (2008) Two new crustaceous soil lichens (Arthoniales) from the Chilean Atacama Desert, South America. Herzogia 21: 25-39.
- Follmann G, Peine J (1999) Camanchaca, a new genus of Roccellaceae (Arthoniales, Loculoascomycetidae) from the Atacama desert, north Chile, South America. Journal of the Hattori Botanical Laboratory 87: 259-276.
- Follmann G, Redón J (1972) Ergänzungen zur Flechtenflora der nordchilenischen Nebeloasen Fray Jorge und Talinay. Willdenowia 6: 431-460.
- Garreaud RD, Vuille M, Campagnucci R, Marengo J (2009) Present-day South American climate. Palaeogeography Palaeoclimatology Palaeoecology 281: 180–195.
- Gonzalez CC, Gandolfo MA, Zamaloa MC, Cúneo NR, Wilf P, Kirk J (2007). Revision of the Proteaceae macrofossil record from Patagonia, Argentina. The Botanical Review 73 (3): 235–66.
- González-Abraham CE, Garcillán PP, Ezcurra E, de Ecorregiones Grupo GDT (2010) Ecoregions of Baja California peninsula: A synthesis. Botanical Sciences 87: 69-82.
- Gottscho, AD (2016) Zoogeography of the San Andreas Fault system: Great Pacific Fracture Zones correspond with spatially concordant phylogeographic boundaries in western North America. Biological Reviews 91(1): 235-254.
- Green TA, Pintado A, Raggio J, Sancho LG (2018). The lifestyle of lichens in soil crusts. The Lichenologist 50(3): 397-410.
- Guerrero PC, Rosas M, Arroyo MT, Wiens JJ (2013). Evolutionary lag times and recent origin of the biota of an ancient desert (Atacama–Sechura). Proceedings of the National Academy of Sciences 110(28): 11469-11474.
- Hafner DJ, Riddle BR (2011) Boundaries and barriers of North American warm deserts: an evolutionary perspective. In Upchurch P, McGowan AJ, Slater CSC (Eds) Palaeogeography and palaeobiogeography: biodiversity in space and time. The Systematics Association Special Volume Series 77, CRC Press, 75-114.
- Hartley AJ, Chong G, Houston J, Mather AE (2005) 150 million years of climate stability: Evidence from the Atacama Desert, northern Chile. Journal of the Geological Society, London 162: 421–441.
- Heinrich S, Zonneveld KA, Bickert T, Willems H (2011) The Benguela upwelling related to the Miocene cooling events and the development of the Antarctic circumpolar current: evidence from calcareous dinoflagellate cysts. Paleoceanography 26(3), PA3209.

- Hernández-Hernández T, Brown JW, Schlumpberger BO, Eguiarte LE, Magallón S (2014) Beyond aridification: multiple explanations for the elevated diversification of cacti in the New World Succulent Biome. New Phytologist 202(4): 1382-1397.
- Hill RS (1991) Leaves of *Eucryphia* (Eucryphiaceae) from tertiary sediments in south-eastern Australia. Australian Systematic Botany 4(3): 481– 497.
- Hill RS (2001). Biogeography, evolution and palaeoecology of *Nothofagus* (Nothofagaceae): The contribution of the fossil record. Australian Journal of Botany 49(3): 321-332.
- Hinchliffe G, Bollard-Breen B, Cowan DA, Doshi A, Gillman LN, Maggs-Kolling G, [...] Pointing SB (2017). Advanced photogrammetry to assess lichen colonization in the hyper-arid Namib Desert. Frontiers in microbiology 8, 2083.
- Jacobs DK, Haney TA, Louie KD (2004) Genes, diversity, and geologic process on the Pacific coast. Annual Review of Earth and Planetary Sciences 32: 601–52.
- Johnson LA, Gowen D, Jensen AB (2013) Cryptic speciation: distinguishing serpentine affiliated sister species Navarretia paradoxiclara and N. paradoxinota from N. intertexta (Polemoniaceae). Phytotaxa 91(2) : 27-38.
- Jung G, Prange M, Schulz M (2014) Uplift of Africa as a potential cause for Neogene intensification of the Benguela upwelling system. Nature Geoscience 7 : 741-747.
- Kamerling MJ, Luyendyk BP (1985) Paleomagnetism and Neogene tectonics of the northern Channel Islands, California: Journal of Geophysical Research 90: 12485-12502.
- Kärnefelt I (1988) Two closely related species of Caloplaca (Teloschistaceae, Lichenes) from Namib Desert. Bothalia 18: 51-56.
- Kelly DC, Norris RD, Zachos JC (2003) Deciphering the paleoceanographic significance of Early Oligocene Braarudosphaera chalks in the South Atlantic. Marine Micropaleontology 49: 49-63
- Kempton EA (2012) Systematics of Eriogonoideae ss (Polygonaceae). Systematic Botany 37(3): 723-737.
- Kistenich S, Timdal E, Bendiksby M, Ekman S (2018). Molecular systematics and character evolution in the lichen family Ramalinaceae (Ascomycota: Lecanorales). Taxon 67: 891-904.
- Klak C, Reeves G, Hedderson T A (2004) Unmatched tempo of evolution in Southern African semi-desert ice plants. Nature 427: 63–65.
- Lalley JS, Viles HA (2005) Terricolous lichens in the northern Namib Desert of Namibia: distribution and community composition. The Lichenologist 37(1): 77-91.
- Lalley JS, Viles HA, Copeman N, Crowley C (2006) The influence of multi-scale environmental variables on the distribution of terricolous lichens in a fog desert. Journal of Vegetation Science 17: 831-838.
- Lancaster LT, Kay KM (2013). Origin and diversification of the California flora: re-examining classic hypotheses with molecular phylogenies. Evolution: International Journal of Organic Evolution 67(4): 1041-1054.
- Larraín-Barrios B, Faúndez-Yancas L, Búrquez A (2018) Plant functional trait structure in two fog deserts of America. Flora 243: 1-10.
- Leavitt SD, Kirika PM, De Paz GA, Huang JP, Hur J-S, Elix JA, Grewe JA, Divakar PK, Lumbsch HT (2018). Assessing phylogeny and historical biogeography of the largest genus of lichen-forming fungi, Xanthoparmelia (Parmeliaceae, Ascomycota). The Lichenologist 50(3): 299-312.
- Levin RA, Wagner WL, Hoch P C, Hahn WJ, Rodriguez A, Baum DA, [...], Sytsma KJ (2004). Paraphyly in tribe Onagreae: insights into phylogenetic relationships of Onagraceae based on nuclear and chloroplast sequence data. Systematic Botany 29(1): 147-164.
- Linder HP (2003) The radiation of the Cape flora, southern Africa. Biological Reviews 78(4): 597-638.
- Linder HP (2005) Evolution of diversity: the Cape flora. Trends in Plant Science 10 (11): 536-541
- Linder HP (2014) The evolution of African plant diversity. Frontiers in Ecology and Evolution 2, 38.

- Lucking R, Tehler A, Bungartz F, Rivas Plata E, Lumbsch HT (2013) Journey from the West: Did tropical Graphidaceae (lichenized Ascomycota: Ostropales) evolve from a saxicolous ancestor along the American Pacific coast? American Journal of Botany 100(5): 844-856.
- Mayrhofer H, Sheard JW, Matzer M (1992) Mobergia (Physciaceae, lichenized Ascomycetes), a new genus endemic to western North America. The Bryologist 95(4): 436-442.
- Millar CI (1996) Tertiary vegetation history. Sierra Nevada Ecosystem Project, Final report to Congress, Volume II, Assessments and Scientific Basis for Management Options, Centers for water and Wildland Resources, Report No. 37. University of California, Davis California.
- Millar CI (2012) Geologic, climatic, and vegetation history of California. In: Baldwin BG, Goldman DH, Keil DJ, Patterson R, Rosatti TJ, Wilken DH (Eds) The Jepson Manual: Vascular plants of California. 2nd Edition. University of California Press, Berkeley, 49–87.
- Millar CI and W. B. Woolfenden (2016) Ecosystems past: prehistory of California vegetation. In: Zavaleta E, Mooney, H. (Eds). Berkeley, University of California Press, Ecosystems of California, Chapter 8, 131-154.
- Miller DD, Graham SA (2018). Late Cenozoic uplift and shortening in the central Coast Ranges and development of the San Joaquin Basin foreland. In: Ingeresoll RV, Lawton TF, Graham SA (Eds.), Tectonics, Sedimentary Basins, and Provenance: A Celebration of William R. Dicinson's Career. Geolgoical Society of American Special Paper 540, 425–440.
- Minnich RA (2007) Climate, Paleoclimate, and Paleovegetation. In: Barbour MG, Keeler-Wolf T, Schoenherr AA (Eds.) Terestrial vegetation of California, 3rd edition. University of California Press, Londo, 41-70.
- Mooney HA (1977) Southern coastal scrub. In: Barbour MG, Major J (Eds). Terrestrial vegetation of California. John Wiley and Sons, New York, 471-487.
- Moran K, Backman J, Brinkhuis H, Clemens SC, Cronin T, Dickens GR, […], Kristoffersen Y (2006) The Cenozoic palaeoenvironment of the Arctic Ocean. Nature 441(7093):601-605.
- Mucina L, Juergens N, Le Roux A, Rutherford MC, Schmiedel U, Esler KJ, Powrie LW, Desmet PG, Milton SJ (2006) Succulent Karoo biome. In: Mucina L, Rutherford MC (Eds) The vegetation of South Africa, Lesotho and Swaziland. South African National Biodiversity Institute, Pretoria, 220-239.
- Nash TH III, Nebeker GT, Moser TJ, Reeves, T (1979) Lichen vegetational gradients in relations to the Pacific Coast of Baja California: The maritime influence. Madroño 26:149-163.
- Nash TH III, Ryan D, Bungartz F (2007) Lichen flora of the greater Sonoran desert region. Vol. 3 Lichens Unlimited, Arizona State University, 1-567.
- Nash TH III, Ryan D, Diederich P, Gries C, Bungartz F (2004) Lichen flora of the greater Sonoran desert region. Vol. 2 Lichens Unlimited, Arizona State University, 1-742.
- Nash TH III, Ryan D, Gries C, Bungartz F (2002) Lichen flora of the greater Sonoran desert region. Vol. 1 Lichens Unlimited, Arizona State University, 1-532.
- Paduan JB, Clague DA, Davis AS (2009) Evidence that three seamounts off southern California were ancient islands, Marine Geology 265: 146-156.
- Paulssen H, Vos D (2017) Slab remnants beneath the Baja California peninsula: Seismic constraints and tectonic implications. Tectonophysics 719: 27–36.
- Peinado M, Alcaraz F, Aguirre JL, Delgadillo J, Aguado I (1995) Shrubland formations and associations in Mediterranean-desert transitional zones of northwestern Baja California. Vegetatio 117(2) : 165-179.
- Peinado M, Delgadillo J, Aguirre JL (2005) Plant associations of El Vizcaíno biosphere reserve, Baja California Sur, Mexico. The Southwestern Naturalist 50(2): 129-150.
- Peinado M, Aguirre JL, Delgadillo J, Macías MÁ (2008) A phytosociological and phytogeographical survey of the coastal vegetation of western North America. Part I: plant communities of Baja California, Mexico. Plant Ecology 196(1), 27.
- Peinado M, Macías MÁ, Ocaña-Peinado FM, Aguirre JL, Delgadillo J (2011) Bioclimates and vegetation along the Pacific basin of Northwestern Mexico. Plant ecology 212(2) : 263-281.

- Peinado M, Macías MÁ, Ocaña-Peinado FM, Aguirre JL, Delgadillo J (2012) Bioclimates and vegetation along the Pacific basin of Northwestern Mexico. Plant Ecology 212(2): 263-281.
- Peine J, Werner B (1995) Follmanniella scutellata gen. et sp. nov., a new genus and species of Roccellaceae (Opegraphales) from the Atacama Desert, north Chile, South America. In: Daniëls FJA, Schulz M, Peine J (Eds.) Flechten Follmann. Contributions to lichenology in Honour of Gerhard Follmann. Geobotanical and Phytotaxonomical Study Group, Botanical Institute, University of Cologne, Cologne, 287-299.
- Pierrehumbert RT (2002) The hydrologic cycle in deep-time climate problems. Nature 419: 191–198.
- Polyak L, Alley RB, Andrews JT. Brigham-Grette J, Cronin TM. Darby DM, Dyke AS, Fitzpatrick JJ, Funder S, Holland M. Jennings AE, Miller GH, Regan MO, Savelle J, Serreze M, St. John K, White. JWC, Wolff E (2010) History of sea ice in the Arctic. Quaternary Science Reviews 29(15-16) 1757-1778.
- Prather LA, Ferguson CJ, Jansen RK (2000) Polemoniaceae phylogeny and classification: implications of sequence data from the chloroplast gene ndhF. American Journal of Botany 87(9): 1300-1308.
- Rafiqpoor D, Kier G, Kreft H (2005) Global centers of vascular plant diversity. Nova Acta Leopoldina NF, 92(342): 61-83.
- Ratay SE, Vanderplank SE, Wilder BT (2014). Island specialists: shared flora of the Alta and Baja California Pacific Islands. Monographs of the Western North American Naturalist 7(1): 161-221.
- Raven PH, Axelrod DI (1978) Origin and relationships of the California flora. Berkeley. University of California Press, 1-134.
- Rebman JP, Roberts N (2012) Baja California Plant field guide. Sunbelt Publications, San Diego, 1-480.
- Rebman JP, Gibson J, Rich K (2016) Annotated checklist of the vascular plants of Baja California, Mexico. San Diego, full text available online.
- Rech JA, Currie BS, Michalski G, Cowan AM (2006) Neogene climate change and uplift in the Atacama Desert, Chile. Geology 34: 761–764.
- Redón J, Lange OL (1983). Epiphytische Flechten im Bereich einer chilenischen „Nebeloase "(Fray Jorge) I. Vegetationskundliche Gliederung und Standortsbedingungen. Flora 174(3-4): 213-243.
- Renner SS, Strijk JS, Strasberg D, Thébaud C (2010). Biogeography of the Monimiaceae (Laurales): a role for East Gondwana and long distance dispersal, but not West Gondwana. Journal Biogeography 37: 1227–1238.
- Riemann H, Exequiel E (2007) Endemic regions of the vascular flora of the peninsula of Baja California, Mexico. Journal of Vegetation Science 18(3): 327-336.
- Rommerskirchen F, Condon T, Mollenhauer G, Dupont L, Schefuß E (2011) Miocene to Pliocene development of surface and subsurface temperatures in the Benguela Current system. Paleoceanography 26: 1-15.
- Rose JP, Kleist TJ, Löfstrand SD, Drew BT, Schönenberger J, Sytsma KJ (2018) Phylogeny, historical biogeography, and diversification of angiosperm order Ericales suggest ancient Neotropical and East Asian connections. Molecular Phylogenetics and Evolution 122: 59-79.
- Rundel PW, Bowler PA (1978) Niebla, a new generic name for the lichen genus Desmazieria (Ramalinaceae). Mycotaxon 6: 497-499.
- Rundel PW, Bowler PA, Mulroy TW (1972) A fog induced lichen community in northwestern Baja California, with two new species of Desmazieria. The Bryologist 75: 501-508.
- Rundel, PW (1978) Ecological relationships of desert fog zone lichens. The Bryologist 81: 277-293.
- Rundel PW, Arroyo MTK, Cowling RM, Keeley JE, Lamont BB, Vargas P (2016) Mediterranean biomes: Evolution of their vegetation, floras, and climate. Annual Review of Ecology, Evolution, and Systematics 47: 383–407.
- Sanchez A, Schuster TM, Kron KA (2009) A large-scale phylogeny of Polygonaceae based on molecular data. International Journal of Plant Sciences 170 (8): 1044-1055.

- Santiago IF, Gonçalves VN, Gómez-Silva B, Galetovic A, Rosa LH (2018) Fungal diversity in the Atacama Desert. Antonie van Leeuwenhoek, 1-16.
- Sauquet H, Ho SY, Gandolfo MA, Jordan GJ, Wilf P, et al. 2012. Testing the impact of calibration on molecular divergence times using a fossil-rich group: the case of *Nothofagus* (Fagales). Systematic Biology 61:289–313.
- Schieferstein B, Loris K (1992) Ecological investigations on lichen fields of the Central Namib. Vegetatio 98: 113-128.
- Schlunegger F, Kober F, Zeilinger G, von Rotz R (2010) Sedimentology-based reconstructions of paleoclimate changes in the Central Andes in response to the uplift of the Andes, Arica region between 19 and 21°S latitude, northern Chile. International journal of earth sciences 99(Suppl 1): S123–S137.
- Schnitzler J, Barraclough TG, Boatwright JS, Goldblatt P, Manning JC, Powell MP, Rebelo T, Savolainen V (2011) Causes of plant diversification in the Cape Biodiversity Hotspot of South Africa, Systematic Biology: 60 (3): 343–357.
- Schultheis LM, Baldwin BG (1999) Molecular phylogenetics of Fouquieriaceae: evidence from nuclear rDNA ITS studies. American Journal of Botany 86(4) : 578-589.
- Schulz N, Boisier JP, Aceituno P (2012) Climate change along the arid coast of northern Chile. International Journal of Climatology 32: 1803–1814.
- Schuster TM, Setaro SD, Kron KA (2013) Age estimates for the buckwheat family Polygonaceae based on sequence data calibrated by fossils and with a focus on the Amphi-Pacific Muehlenbeckia. PLoS One, 8(4), e61261.
- SEINet, Arizona and New Mexico Chapter (2019) Accessed for *Petalonyx linearis*, Interactive Map and other species where cited. http://swbiodiversity.org/seinet/taxa/index.php?taxon=1611 , accessed 03 Sep 2019.
- Sérusiaux E, Wessels D (1984) Santessonia (Lecanorales, Buelliaceae) in the Namib Desert (South West Africa). Mycotaxon 19: 479-502.
- Sheahan MC, Chase MW (2000) Phylogenetic relationships within Zygophyllaceae based on DNA sequences of three plastid regions, with special emphasis on Zygophylloideae. Systematic Botany 25(2) : 371-385.
- Shreve, F (1936) The transition from desert to chaparral in Baja California. Madroño 3: 257-264.
- Shreve F, Wiggins (1964). Vegetation and flora of the Sonoran Desert (Vol. 591). Stanford University Press.
- Sipman HJM (1995) Heterodermia pinnata sp. nov. and Heterodermia follmannii sp. nov. (Physciaceae, Lecanorales), two new lichen species from north Chile, South America. In Daniëls FJA, Schulz M, Peine J (eds.): Flechten Follmann. Contributions to lichenology in Honour of Gerhard Follmann. Geobotanical and Phytotaxonomical Study Group, Botanical Institute, University of Cologne, Cologne, 329-336.
- Sork VL, Werth S (2014) Phylogeography of Ramalina menziesii, a widely distributed lichen-forming fungus in western North America. Molecular Ecology 23(9): 2326-2339.
- Spjut RW (1995) Occurrence of Mobergia calculiformis (Physciaceae, Lecanorales) in the northern Vizcaino Desert of Baja California, Mexico. In: Daniëls FJA, Schulz M, Peine J (eds.): Flechten Follmann. Contributions to lichenology in Honour of Gerhard Follmann. Geobotanical and Phytotaxonomical Study Group, Botanical Institute, University of Cologne, Cologne,475-482.
- Spjut RW (1996) Niebla and Vermilacinia (Ramalinaceae) from California and Baja California. Sida, Botanical Miscellany 14: 1-208.
- Tehler A, Irestedt M, Wedin M, Ertz D (2009) Origin, evolution and taxonomy of American Roccella (Roccellaceae, Ascomycetes). Systematics and Biodiversity 7(3): 307-317.
- Sytsma KJ, Litt AM, Zjhra L, Pires JC, Nepokroeff M, Conti E, Walker J, Wilson P G (2004) Clades, clocks, and continents: historical and biogeographical analysis of Myrtaceae, Vochysiaceae, and relatives in the southern hemisphere. International Journal of Plant Science 165 (4 Supplement): S85–S105.

- Taylor F, Hill RS (1996) A phylogenetic analysis of the Eucryphiaceae. Australian Systematic Botany 9: 735–48.
- Turner RM, Bowers JE, Burgess TL (1995) Sonoran Desert Plants: An Ecological Atlas. University of Arizona Press, 1–501.
- Turner RM, Brown DE (1982) Sonoran desertscrub. Available at https://repository.arizona.edu/bitstream/handle/10150/550979/dp_04_01-04-181-221.pdf?sequence=1
- Vahlenkampa M, Niezgodzkib I, De Vleeschouwera D, Lohmannb G, Bickerta T, Pälike H (2018) Ocean and climate response to North Atlantic seaway changes at the onset of long-term Eocene cooling. Earth and Planetary Science Letters 498: 185–195.
- Vanderplank SE (2011) The flora of greater San Quintín, Baja California, Mexico (2005–2010). Aliso 29(2): 65–103.
- Vidal MA, Soto ER, Veloso A (2009) Biogeography of Chilean herpetofauna: Distributional patterns of species richness and endemism. Amphib-reptil 30: 151–171.
- Vogel S (1955) Niedere "Fensterpflanzen" in der südafrikanischen Wüste. Eine ökologische Schilderung. Beitrage zur Biologie der Pflanzen 31: 45–135.
- Ward JD, Corbett I (1990) Towards an age for the Namib. In Seely MK (Ed) Namib ecology : 25 years of Namib research. Transvaal Museum Monograph 7, Transvaal Museum, Pretoria, 17-26.
- Wedin M, Döring H, Nordin A, Tibell L (2000). Small subunit rDNA phylogeny shows the lichen families Caliciaceae and Physciaceae (Lecanorales, Ascomycotina) to form a monophyletic group. Canadian Journal of Botany 78(2): 246-254.
- White LD, Garrison RE, Barron JA (1992) Miocene intensification of upwelling along the California margin as recorded in siliceous facies of the Monterey Formation and offshore DSDP sites. Geological Society, London, Special Publications 64:429–442.
- Wiggins IL (1980) Flora of Baja California. Stanford University Press, 1025 pp.
- Wirth V (2010a) Flechtengesellschaften der Namibwüste. Carolinea 68: 49-60.
- Wirth V (2010b) Lichens of the Namib Desert: A guide to their identification. Klaus Hess Verlag, Göttingen, 1-96.
- Wirth V, Elix JA (2006) Pertusaria pseudomelanospora sp. nova, a new saxicolous lichen species from the Namib desert. Carolinea 63: 95-96.
- Wolfe J. A (1964) Fingerrock Wash Southwestern Nevada. Geological Survey Professional Paper 454, N1-N32. Index, 12 plages.
- Wu SD, Zhang LJ, Lin L, Yu SX, Chen ZD, Wang W (2018) Insights into the historical assembly of global dryland floras: The diversification of Zygophyllaceae. BMC evolutionary biology 18(1), 166.
- Zachos JC, Dickens GR, Zeebe RE (2008) An early Cenozoic perspective on greenhouse warming and carbon-cycle dynamics. Nature 451 :279–283.
