# Supplementary material for: The fruticose genera in the Ramalinaceae (Ascomycota, Lecanoromycetes): their diversity and evolutionary history
Source: MycoKeys. 2020 Sep 11;73:1–68. doi: 10.3897/mycokeys.73.47287 (PMC7501315; doi:10.3897/mycokeys.73.47287)
Supplement: Supplementary material 2 — Table S2. PCR conditions and primers for each locus [file mycokeys-73-001-s002.pdf]

Locus	Primer name	Primer sequence (5’-3’)	Annealing temperature (◦C)	Reference
EF-1a	EF1F	RGACAAGRCTCACATCAACGTSGT	52	Johannesson et al. 2000
	EF1R	CCAGTRATCATGTTCTTGATGAART		Johannesson et al. 2000
GAPDH	GPDF	YGGTGTCTTCACCACCACYGASAA	51	Johannesson et al. 2000
	GPDR	RTANCCCCAYTCRTTRTCRTACCA		Johannesson et al. 2000
ITS	ITS1F	CTTGGTCATTTAGAGGAAGTAA	52	Gardes and Bruns 1993
	ITS4	TCCTCCGCTTATTGATATGC		White et al. 1990
LSU	LR7	TACTACCACCAAGATCT	54	Vilgalys and Hester 1990
	LR0R	ACCCGCTGAACTTAAGC		Rehner and Samuels 1994
LSU	LRramF	TACACACTCCTTARCGGATTC	52	Designed for his study
	LRramR	GASGAAAAGAAACCAACMG		Designed for this study
RPB1	RPB1-VHAFasc	ADTGYCCYGGYCATTTYGGT	52	Hofstetter et al. 2007
	RPB1-VH6R	ATGACCCATCATRGAYTCCTTRTG		Hofstetter et al. 2007
RPB2	fRPB2-5F	GAYGAYMGWGATCAYTTYGG	52	Liu et al. 1999
	fRPB2-7CR	CCCATRGCTTGYTTRCCCAT		Liu et al. 1999
