# Supplementary material for: The fruticose genera in the Ramalinaceae (Ascomycota, Lecanoromycetes): their diversity and evolutionary history
Source: MycoKeys. 2020 Sep 11;73:1–68. doi: 10.3897/mycokeys.73.47287 (PMC7501315; doi:10.3897/mycokeys.73.47287)
Supplement: Supplementary material 4 — Table S4. Time calibration [file mycokeys-73-001-s004.pdf]

Clade name	Mean	95% CI
Niebla **crown**	**13.14**	7.05 – 21.05
Vermilacinia **crown**	**22.47**	13.44 – 32.26
Niebla* + *Vermilacinia	**30.05**	17.27 – 43.11
Niebla* + *Vermilacinia* + 3 spp. *Cliostomum	**48.94**	28.23 – 62.61
Namibialina crown	**19.71**	8.47 – 32.75
Ramalina **crown**	**43.31**	30.22 – 56.26
Namibialina* + *Ramalina	**48.45**	35.13 – 63,66
Fruticose **Ramalinaceae +** ***Cliostomum*** **s. str.**	**55.53**	40.23 – 72.29
**Fruticose Ramalinaceae + all** ***Cliostomum***	**67.72**	48.81 – 88.12
