# Supplementary material for: The fruticose genera in the Ramalinaceae (Ascomycota, Lecanoromycetes): their diversity and evolutionary history
Source: MycoKeys. 2020 Sep 11;73:1–68. doi: 10.3897/mycokeys.73.47287 (PMC7501315; doi:10.3897/mycokeys.73.47287)
Supplement: Supplementary material 5 — Table S5. Comparison of the identification of the Niebla collections [file mycokeys-73-001-s005.pdf]

Species number	Epithet used for *Niebla* identification following Spjut (1996)	Number of accessions identified under each epithet and included in the 6-loci analysis	Number of species as delimited by BPP included under that epithet	Number of species as delimited by STACEY Included under that epithet
1	*N. arenaria*	2	2	2
2	*N. brachyura*	4	3	2
3	*N. contorta*	4	2	2
4	*N. dilatata*	3	2	2
5	*N. eburnea*	4	3	3
6	*N. effusa* (incl. « aff. »)	5	3	3
7	*N. fimbriata* (incl. « aff. »)	4	2	2
8	*N. flabellata*	7	4	3
9	*N. flagelliforma*	3	4	4
10	*N. homalea*	10	3	2
11	*N. josecuervoi*	5	4	2
12	*N. juncosa*	2	1	1
13	*N. juncosa* var. *spinulifera*	3	3	3
14	*N. limicola*	2	2	2
15	*N. lobulata* (incl. « aff. »)	6	4	4
16	*N. marinii*	2	1	1
17	*N. palmeri* (incl. « aff. »)	4	2	2
18	*N. podetiaforma*	4	4	1
19	*N. podetiaforma/caespitosa*	1	1	1
20	*N. pulchribarbara*	1	1	1
21	*N. rugosa*	1	1	1
22	*N. siphonoloba*	1	1	1
23	*N. sorocarpia*	2	2	1
24	*N. spatulata*	4	1	1
25	*N. suffnessii*	2	1	1
26	*N. testudinaria*	3	1	1
27	*N. turgida*	2	1	1
28	*N. undulata*	4	3	2
29	*N.* sp.	2	2	3

Methodology :

- Each accession used for the 6-loci analysis has been identified following Spjut (1996) using morphological and chemical characters. Each epithet appears one to ten times in the phylogenetic tree : these data are presented in the third column.
- The species delimitation method bP&P assigned each accession to a species using the molecular data. The second column points to the number of different species delimited by bP&P that were assigned to each epithet used following Spjut (1996).
- The same applies for the last column, except that the species delimitation method is here STACEY.
