# Supplementary material for: The fruticose genera in the Ramalinaceae (Ascomycota, Lecanoromycetes): their diversity and evolutionary history
Source: MycoKeys. 2020 Sep 11;73:1–68. doi: 10.3897/mycokeys.73.47287 (PMC7501315; doi:10.3897/mycokeys.73.47287)
Supplement: Supplementary material 6 — Table S6. Data for the Niebla collections studied [file mycokeys-73-001-s006.pdf]

Species number (as delimited by BPP)	San Antonio BC Mexico	San Quintin BC Mexico	Punta Baja BC Mexico	San Rosallilita BC Mexico	Santo Domingo BC Mexico	Punta Rosallilita BC Mexico	Rosario BC Mexico	Guerrero Negro BCS Mexico	Bahia Ascunsion BCS Mexico	El Elephante BCS Mexico	Bahia Tortugas BCS Mexico	California USA	TOTAL (localities per species)
1	X	—	X	X	—	X	X	—	—	—	—	—	5
2	—	—	—	—	X	—	—	—	—	—	—	—	1
3	—	—	X	—	—	—	—	—	—	—	—	—	1
4	—	—	—	—	X	—	—	—	—	—	—	—	1
5	—	—	—	X	—	—	—	—	—	—	—	—	1
6	—	—	—	—	—	X	—	—	—	—	—	—	1
7	—	—	—	—	—	X	—	—	—	—	—	—	1
8	—	—	—	—	—	—	—	—	X	—	X	—	2
9	—	—	—	—	—	—	—	—	—	—	X	—	1
10	—	X	—	—	—	—	X	—	—	—	—	X	3
11	—	X	—	—	—	—	X	—	—	—	—	X	3
12	—	—	—	—	—	—	—	—	—	—	—	X	1
13	X	—	—	—	—	—	—	—	—	—	—	—	1
14	X	X	—	—	—	—	X	—	—	—	—	—	3
15	—	—	—	—	—	—	—	—	—	—	X	—	1
16	—	—	—	—	X	—	—	—	—	—	X	—	2
17	X	—	X	—	—	—	X	—	—	X	—	—	4
18	—	—	—	—	—	—	—	—	—	X	—	—	1

19	—	—	—	—	—	—	—	—	—	X	—	—	1
20	—	X	—	—	—	—	X	—	—	—	—	—	2
21	—	X	—	—	—	—	—	—	—	—	—	—	1
22	—	—	—	—	X	X	—	—	—	—	—	—	2
23	—	—	—	—	—	—	—	—	—	X	—	—	1
24	—	—	—	X	X	X	—	—	—	—	—	—	3
25	X	X	X	—	—	—	—	—	—	—	—	—	3
26	—	X	X	—	—	—	—	—	—	—	—	—	2
27	—	—	X	—	—	—	—	X	—	—	—	—	2
28	—	X	—	—	X	—	—	—	—	—	—	—	2
29	—	—	—	—	—	X	—	—	—	—	—	—	1
30	X	—	—	—	X	—	—	—	—	—	—	—	2
31	—	—	—	—	—	—	—	X	—	—	—	—	2
32	—	—	—	—	—	—	—	—	—	—	X	—	1
33	—	—	—	—	—	—	—	—	—	—	X	—	1
TOTAL (species per locality)	6	8	6	3	7	6	6	2	1	4	6	3	
Species total for each three "states"	22							12				3	
