# Supplementary material for: The fruticose genera in the Ramalinaceae (Ascomycota, Lecanoromycetes): their diversity and evolutionary history
Source: MycoKeys. 2020 Sep 11;73:1–68. doi: 10.3897/mycokeys.73.47287 (PMC7501315; doi:10.3897/mycokeys.73.47287)
Supplement: Supplementary material 8 — Identification of the chemistry of Ramalina rosacea [file mycokeys-73-001-s008.pdf]

Supplementary File S8

Identification of the secondary metabolites produced by *Ramalina rosacea*, a species found on rock sea shores in the Western Mediterranean area, and confined to two localities: France, Corsica, Cavallo islet in the Lavezzi archipelago and Spain, Alméria, Cabo de Gata.

Extraction of the secondary metabolites was made in cold acetone, and TLC were performed in solvent C and G (Culberson et al. 1981; Orange et al. 2010). Both plates were dried and sprayed with a 10% H_2SO_4 solution and eventually heated at c. 120°C in oven.

Samples for comparison were [all specimens preserved in LG]: *Pleurosticta acetabulum* (Belgium, Namur, 1973, coll. E. Sérusiaux 555); *Parmelia saxatilis* (Belgium, Liège, 2020, coll. E. Sérusiaux s.n.); *Parmotrema perlatum* (France, Pas-de-Calais, 1960, coll. J. Lambinon 1013); *Parmotrema crinitum* (France, Pyrénées-Atlantiques, 1989, coll. E. Sérusiaux 10584). Accessions of *Ramalina rosacea*: France, Cavallo, D. & O. Gonnet s.n. (hb, LG DNA 4642); Spain, Alméria, Cabo de Gata, P. van den Boom 3835 (hb van den Boom, LG DNA 425 and 426).

Plates are reproduced below. Plate developed in solvent G includes identification of accessions (below) and references to ß-depsidones of the stictic ac. group; nomenclature follows Culberson et al. (1981).

References:

- Culberson CF, Culberson WL, Johnson A (1981) A standardized TLC analysis of β-orcinol depsidones. *Bryologist* 84: 16-29.
- Gonnet D, Gonnet O, Gardiennet A, Roux C (2017) Les lichens et champignons lichénicoles de l'île de Cavallo (archipel des Lavezzi, Corse). Ecologia Mediterranea 43 : 171-175.
- Orange A, James PW, White FJ (2010) Microchemical methods for the identification of lichens. British Lichen Society, London

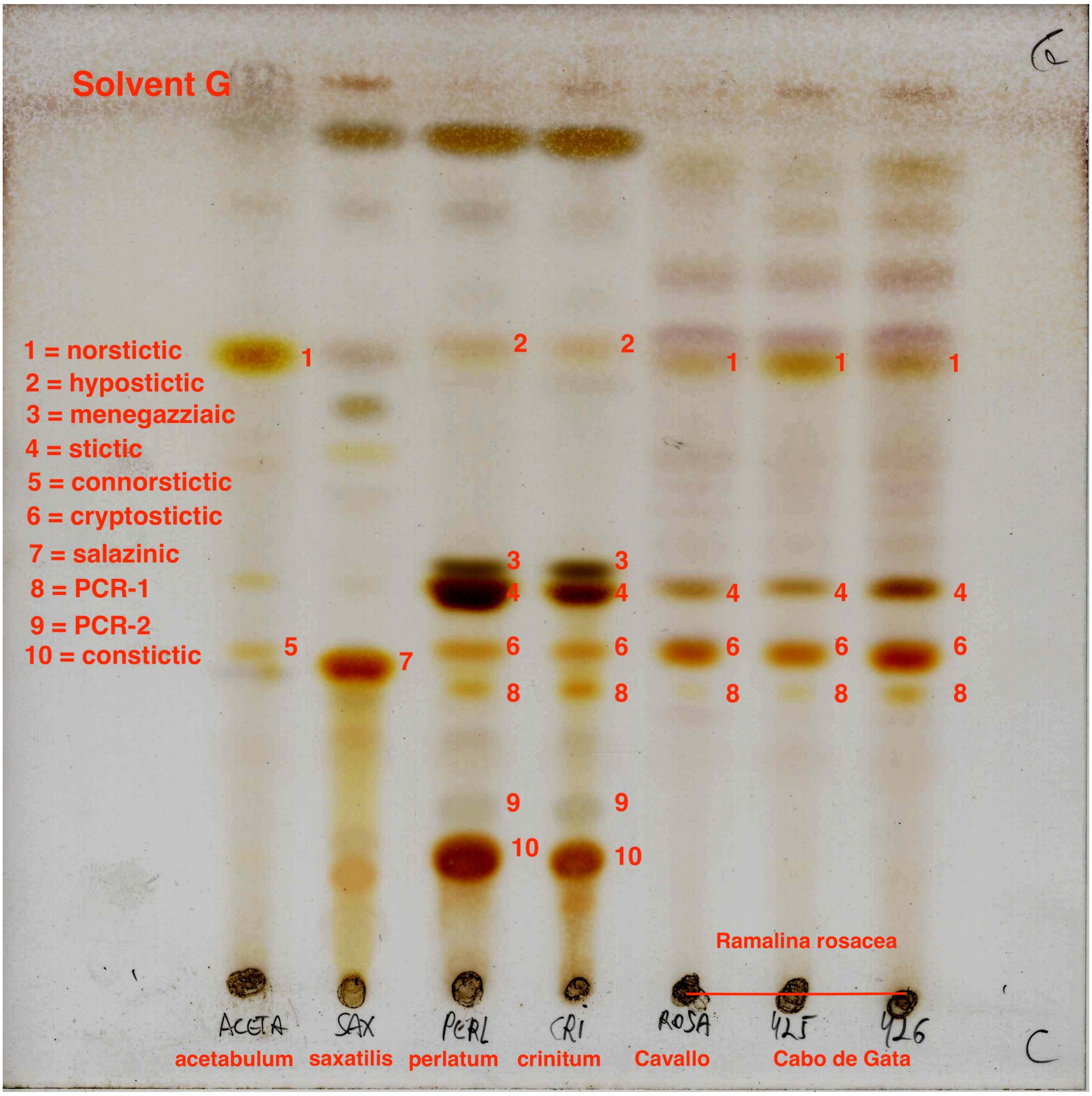

Solvent G
1 = norstictic
2 = hypostictic
3 = menegazziaic
4 = stictic
5 = connorstictic
6 = cryptostictic
7 = salazinic
8 = PCR-1
9 = PCR-2
10 = constictic
Ramalina rosacea
ACETA
SAX
PERL
CRI
ROSA
425
426
acetabulum
saxatilis
perlatum
crinitum
Cavallo
Cabo de Gata

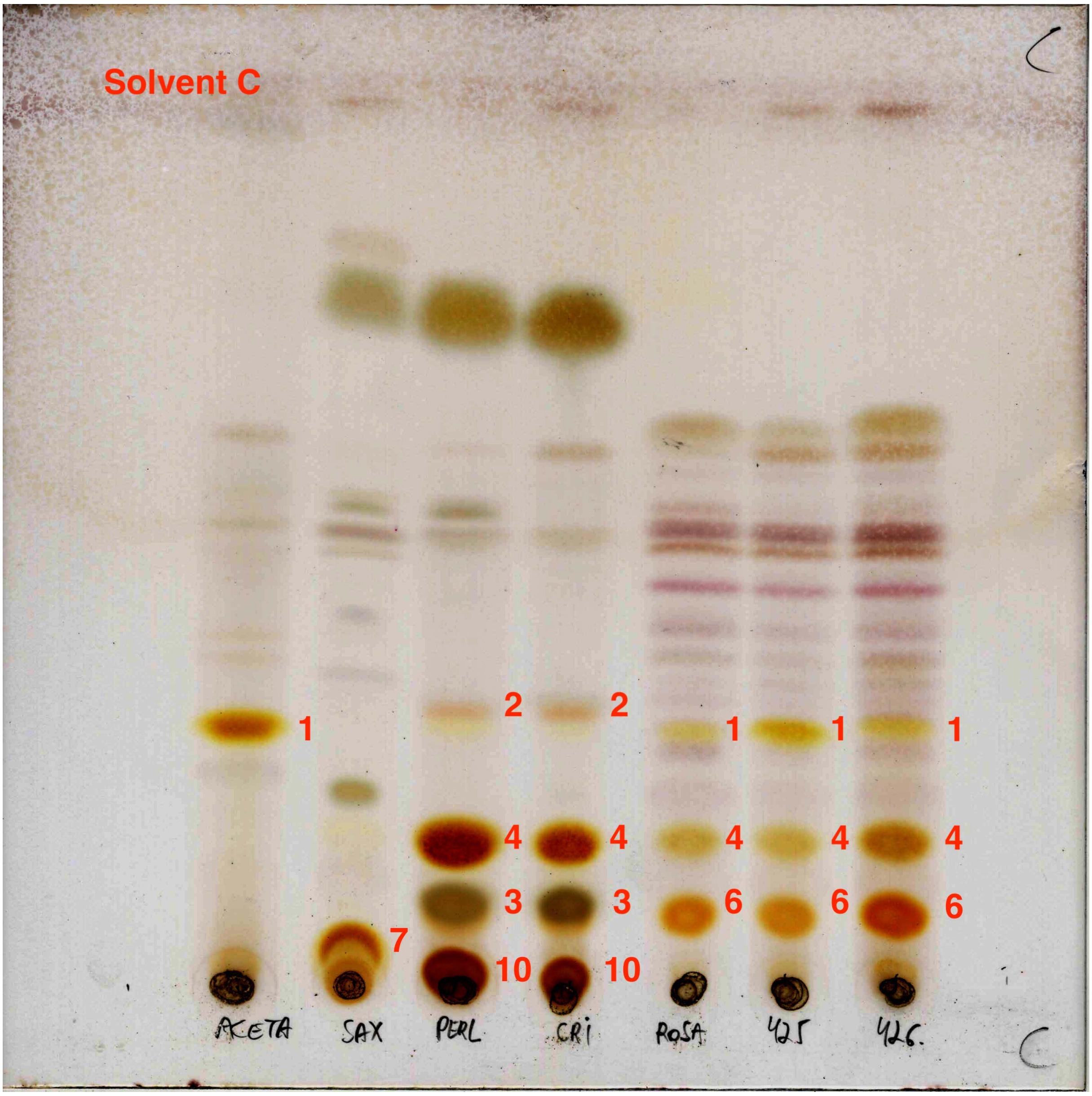
Solvent C
1
2
2
1
1
1
4
4
4
4
4
3
3
6
6
6
7
10
10
ACETA
SAX
PERL
CRI
ROSA
425
426.
